# Non-Palladium-Catalyzed Approach to the Synthesis of (*E*)-3-(1,3-Diarylallylidene)Oxindoles

**DOI:** 10.3390/molecules27165304

**Published:** 2022-08-19

**Authors:** Jahyun Koo, Minsu Kim, Kye Jung Shin, Jae Hong Seo

**Affiliations:** Integrated Research Institute of Pharmaceutical Sciences, College of Pharmacy, The Catholic University of Korea, Bucheon-si 420-743, Korea

**Keywords:** 3-(1,3-diarylallylidene)oxindole, knoevenagel condensation, allylic oxidation, wittig reaction, aldol reaction, non-palladium-catalyzed

## Abstract

Two novel synthetic approaches for synthesizing (*E*)-3-(1,3-diarylallylidene)oxindoles from oxindole were developed. All previously reported methods for synthesizing 3-(1,3-diarylallylidene)oxindoles utilized palladium-catalyzed reactions as a key step to form this unique skeleton. Despite high efficiency, palladium-catalyzed reactions have limitations in terms of substrate scope. Especially, an iodoaryl moiety cannot be introduced by the previous methods due to its high reactivity toward the palladium catalyst. Our Knoevenagel/allylic oxidation/Wittig and Knoevenagel/aldol/dehydration strategies complement each other and show broad substrate scope, including substrates with iodoaryl groups. The current methods utilized acetophenones, benzylidene phosphonium ylides, and benzaldehydes that are commercially available or easily accessible. Thus, the current synthetic approaches to (*E*)-3-(1,3-diarylallyldiene)oxindoles are readily amendable for variety of oxindole derivatives.

## 1. Introduction

**Scheme 1:**
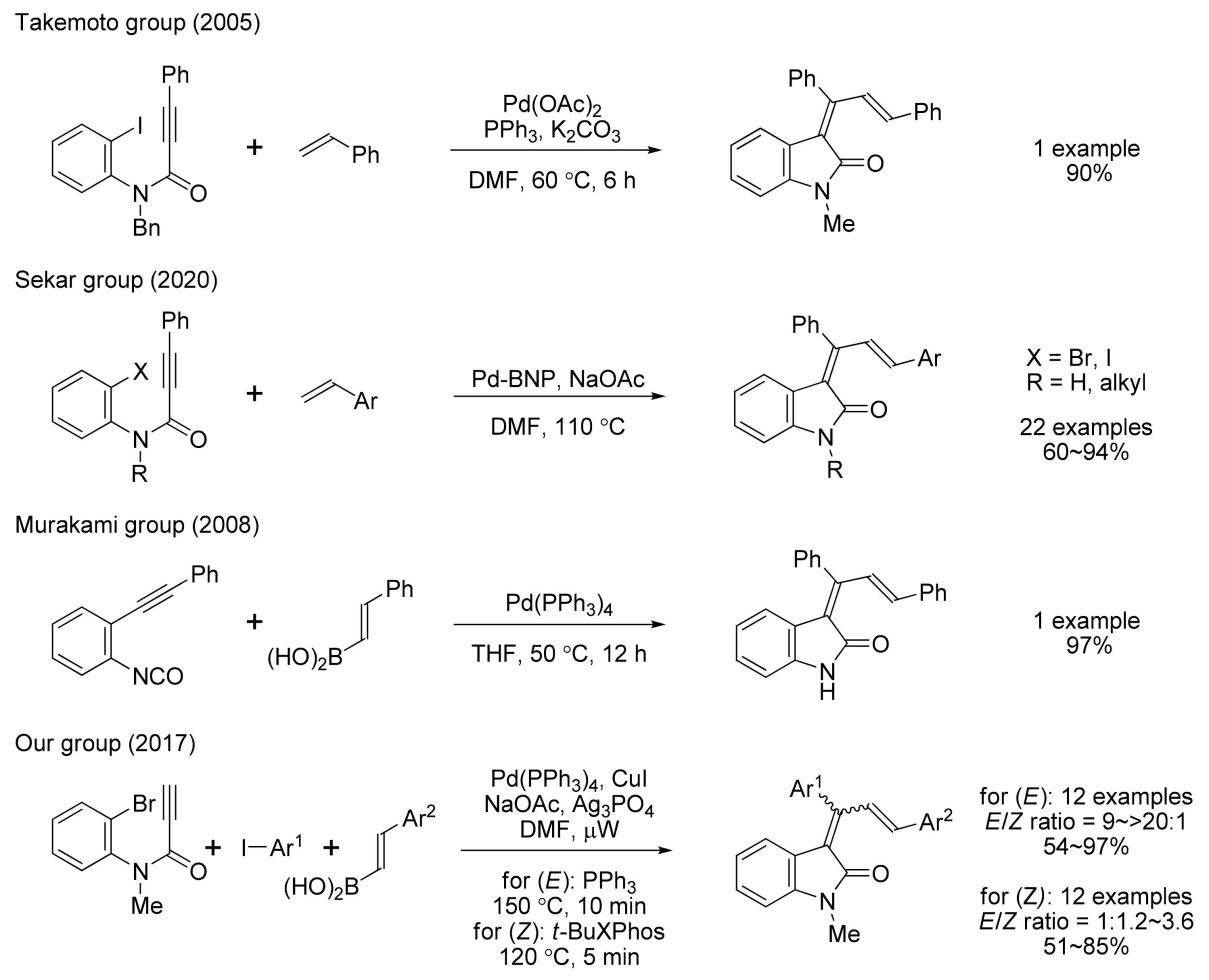
Previous synthetic approaches to 3-(1,3-diarylallylidene)oxindoles.

3-(Diarylmethylene)oxindoles belong to a major oxindole family that has recently been reported to have novel biological activities, such as AMPK activation [1] and estrogen receptor-related anticancer activity against breast cancer [2]. As valuable derivatives of 3-(diarylmethylene)oxindoles in the field of medicinal chemistry, 3-(1,3-diarylallylidene)oxindoles, which have a vinyl linker at the 3-methylene position, have attracted considerable attention from synthetic chemists, and several synthetic methods have been reported (Scheme 1) [3,4,5,6]. In 2005, a 3-(1,3-diarylallylidene)oxindole was first synthesized by Takemoto et al., utilizing double Heck reactions [3]. Recently, Sekar et al., improved this approach using palladium binaphthyl nanoparticles (Pd-BNPs) as a catalyst to broaden the substrate scope and allow easy separation [4]. In 2008, Murakami et al., developed another synthetic method featuring palladium-catalyzed oxidative cyclization of 2-(alkynyl)isocyanate, followed by the Suzuki-Miyaura reaction with styrylboronic acid [5]. As part of our ongoing efforts to identify novel synthetic methods for 3-methyleneoxindole derivatives [6,7,8,9,10], we recently reported a palladium-catalyzed multicomponent tandem reaction, which allowed a stereoselective approach to (*E*)- and (*Z*)-isomers of 3-(1,3-diarylallylidene)oxindoles by changing phosphine ligands, reaction temperature, and time [6]. Although several synthetic methods for 3-(1,3-diarylallylidene)oxindoles have already been developed, as described above, the narrow substrate scope and/or limited accessibility of reagents when using these methods necessitate the development of a more general approach to this unique skeleton. In all previous methods palladium-catalyzed reactions were the key reactions, which greatly limited the range of products to which these procedures could be applied. A few non-palladium-catalyzed approaches to 3-allylideneoxindoles have been reported, but they cannot be applied to the synthesis of 3-(1,3-diarylallylidene)oxindoles [11,12]. Therefore, we attempted to develop a novel synthetic method for 3-(1,3-diarylallylidene)oxindoles with a wide substrate scope using commercially available or easily accessible reagents, and not involving palladium-catalyzed reactions.

## 2. Results and Discussion

### 2.1. Unsuccessful Direct Knoevenagel Approach

**Scheme 2:**
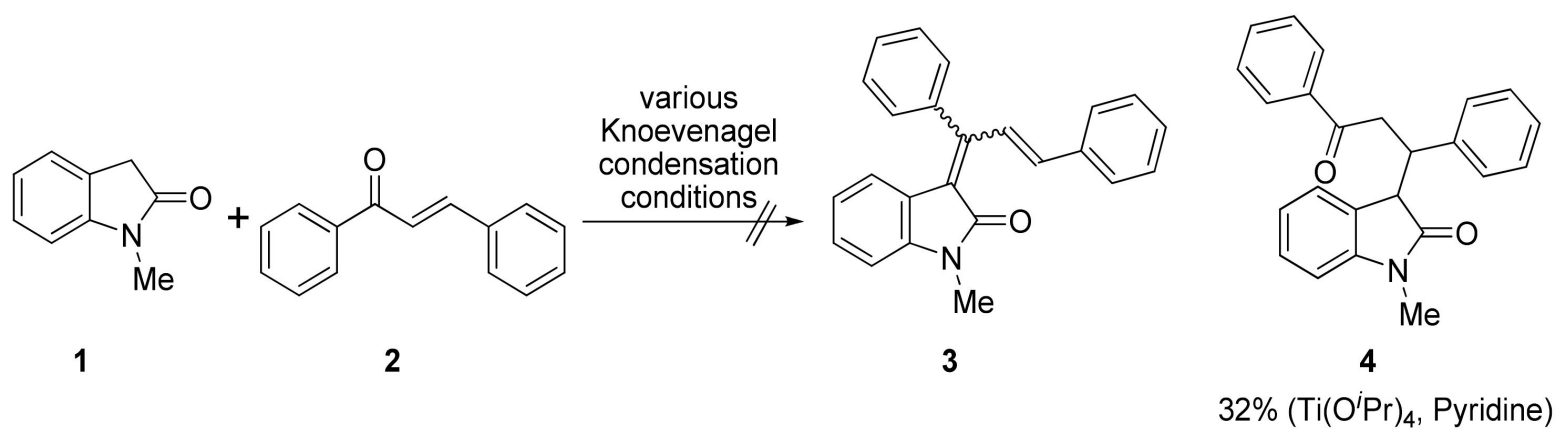
Knoevenagel condensation of oxindole **1** with chalcone (**2**).

To develop a novel and efficient approach for synthesizing 3-(1,3-diarylallylidene)oxindoles, we first examined the feasibility of direct Knoevenagel condensation between oxindole **1** and chalcone (**2**) (Scheme 2). Several Knoevenagel condensations of oxindoles with α,β-unsaturated aldehydes have been reported [13,14,15,16,17], but there is no precedent for using α,β-unsaturated ketones, such as chalcone. Despite intensive efforts, the desired 3-(1,3-diphenylallylidene)oxindole **3** could not be formed, as in previous studies. Only a small amount of 1,4-addition adduct **4** was obtained under Ti(O*^i^*Pr)_4_/pyridine-mediated conditions [14,18].

### 2.2. Stepwise Approach 1 (Knoevenagel/Allylic Oxidation/Wittig)

**Scheme 3:**
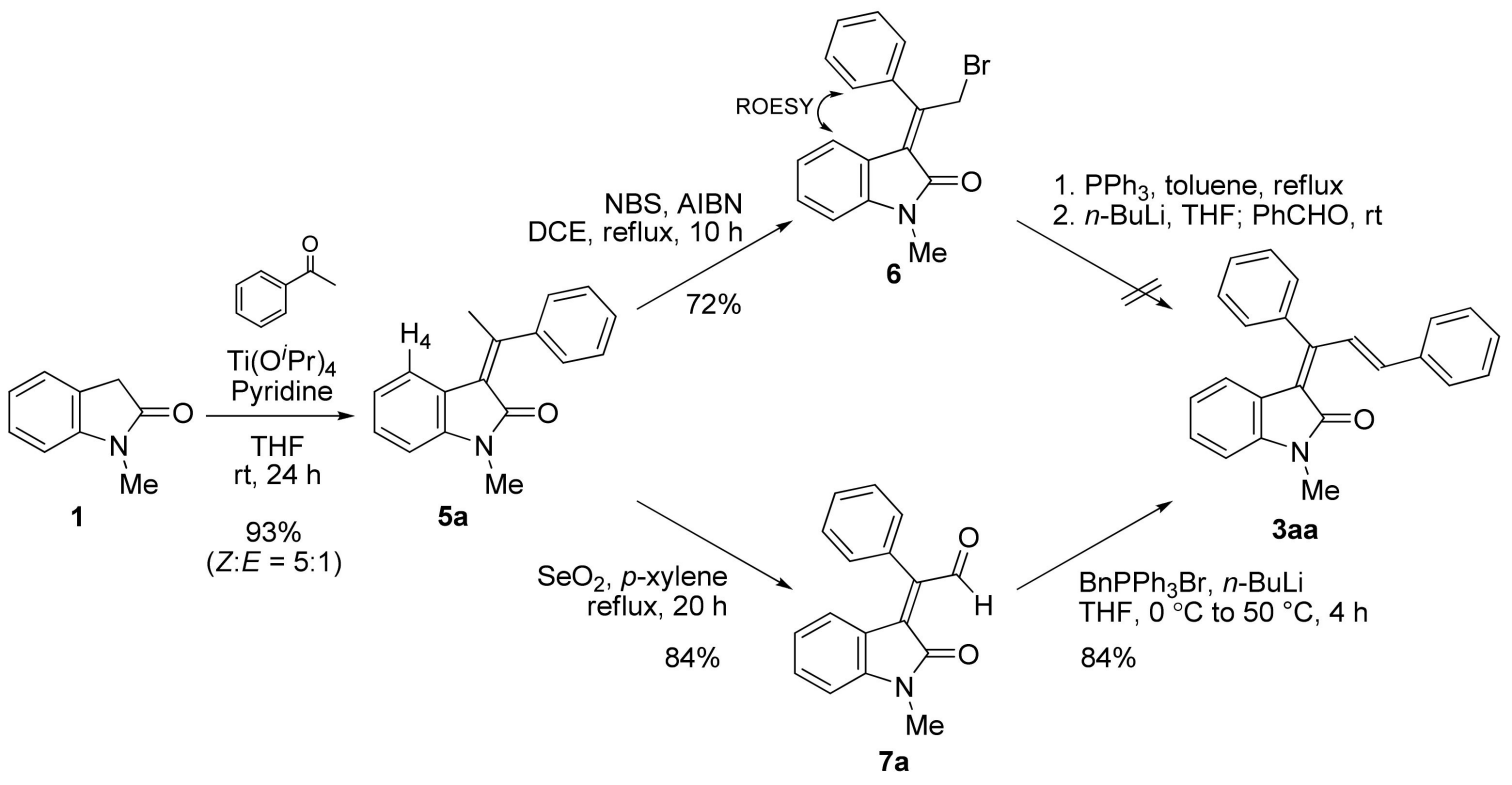
Stepwise approach to 3-(1,3-diphenylallylidene)oxindole **3aa**.

The disappointing results of the direct Knoevenagel strategy prompted us to apply a stepwise approach (Scheme 3). Using an acetophenone as a “partner” of Ti(O*^i^*Pr)_4_/pyridine-mediated Knoevenagel condensation, 3-methyleneoxindole **5** was easily obtained from oxindole **1** in 93% yield with good *Z*-stereoselectivity (*Z*:*E* = 5:1). The preference for the *Z*-isomer could be explained by a chelation-controlled transition state [14]. The geometry of each isomer was confirmed by comparing ^1^H NMR data for **(*E*)-5a** [19] and the chemical shift of H_4_ [6.84 ppm (*Z*-isomer), 6.14 ppm (*E*-isomer)]. On ^1^H NMR analysis of known 3-arylmethylenoxindoles, the chemical shift of H_4_ is upfield (generally 6.50–6.00 ppm) compared to the usual aromatic area when the aryl group attached to the 3-methylene position of oxindole is located close to H_4_ [6,9]. Next, the methyl group should be transformed into a proper functional group to introduce the second olefin. Under radical bromination conditions [20], allylic bromide **6** was obtained as a single geometric isomer, regardless of the geometry of **5a**. The structure of **6** was elucidated by intensive NMR studies, including HSQC, HMBC, COSY, and ROESY. In addition, the chemical shift of H_4_ was 6.11 ppm, which supported the (*Z*)-geometry of **6**. The Krische group reported similar scrambling of olefin geometry during radical allylic bromination [21]. Unfortunately, the Wittig reaction of the corresponding ylide derived from **6** did not afford the desired **3aa**. Given these disappointing results, we exchanged the positions of the functional groups in the Wittig reaction. Therefore, the second functionalization of the methyl group was allylic oxidation. After analyzing several oxidation conditions, we found that SeO_2_ oxidation [22] afforded aldehyde **7a** in 84% yield, including as a single geometric isomer from both (*Z*)- and (*E*)-**5a**. Interestingly, the olefin geometry of **7a** had the *Z*-configuration, which was confirmed by comparison with previously reported ^1^H NMR data for **7a** [23]. In addition, the chemical shift of H_4_ for **7a** also appeared at 6.26 ppm. The *Z*-stereoselectivity of allylic oxidation may have been due to coordination of the oxindole carbonyl group to selenium [24]. However, considering the high reaction temperature, the possibility of isomerization of **(*E*)-7a** to the more stable **(*Z*)-7a** during the reaction could not be excluded. Then, the Wittig reaction of aldehyde **7a** with the ylide proceeded smoothly and provided the desired **3aa** in 84% yield. The ^1^H and ^13^C NMR data of **3aa** exactly matched the results obtained in our previous study [6].

By applying the successful stepwise approach to 3-(1,3-diphenylallylidene)oxindole, we investigated the substrate scope of aldehyde **7** (Table 1). The Knoevenagel condensation of oxindole **1** and acetophenones with chloro, nitro, and methoxy substituents proceeded well, affording **5b**–**d** in good yield (77–95%) with *Z*-stereoselectivity (*Z*:*E* = 4–10:1) (Entries 1–3). SeO_2_-mediated allylic oxidation of **5b** and **5c** also proceeded smoothly to afford the corresponding **7** in 85% and 87% yield, respectively. However, the oxidation of **5d**, bearing a methoxy substituent at the aryl group, was unsuccessful and resulted in complete decomposition. Unfortunately, neither a lower reaction temperature nor other oxidation conditions overcame this decomposition problem.

Setting aside the problematic **5d**, we assessed the substrate scope of the final Wittig reaction of **5a**–**c** with ylides bearing various substituents on the aryl group (Table 2). Fortunately, all reactions afforded 3-(1,3-diarylallylidene)oxindoles **3,** regardless of the substituent combination, in moderate to good yield (53–95%) (Entries 1–12).

### 2.3. Stepwise Approach 2 (Knoevenagel/Aldol/Dehydration)

**Scheme 4:**
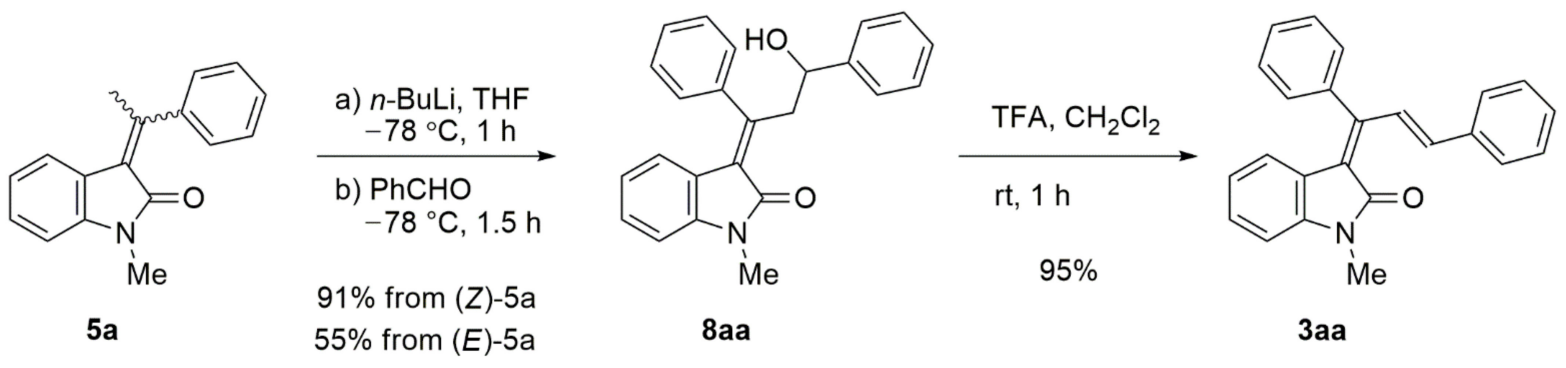
The second stepwise approach utilizing aldol reaction/dehydration.

As shown above, the allylic oxidation/Wittig reaction strategy allowed synthesis of various 3-(1,3-diarylallylidene)oxindoles **3** from Knoevenagel adducts **5**. However, decomposition of **5d** in SeO_2_ oxidation limited the application scope of this strategy. Therefore, we investigated another stepwise approach, which could be applied to **5d** and overcome the limitation of the first strategy. Based on the fact that the functional handle of the methyl group was located at the γ-position of the α,β-unsaturated carbonyl moiety, we assumed that aldol reaction may be feasible. In addition, several examples of similar aldol reactions were found in the literature [24,25,26]. According to the results of base screening, only *n*-BuLi could provide the desired aldol product, **8aa**, in good yield (Scheme 4). The olefin geometry of **8aa** was assigned as *E*, as the chemical shift of H_4_ appeared at 6.14 ppm. Notably, unlike bromination and allylic oxidation, the olefin geometry of **5a** significantly affected the aldol reaction rate. Under optimized conditions, (*Z*)-**5a** was rapidly converted to **8aa** in 91% yield, while the reaction of (*E*)-**5a** afforded **8aa** in 55% yield. A longer reaction time and/or elevated reaction temperature did not increase product yield. The difference in reaction rate may have been caused by the lithiated intermediate from (*E*)-**5a** assuming a stable chelated form via coordination of the oxindole carbonyl group. Dehydration of **8aa** proceeded smoothly under acidic conditions to provide **3aa** in 95% yield [27,28]. 

Next, we examined whether the second stepwise approach (aldol/dehydration) was applicable to **5d** (Table 3). The first aldol reaction of **5d** proceeded well with various benzaldehydes, giving aldol adduct **8** in moderate to good yields (Entries 1–3 and 5). With the exception of **8dc**, TFA-mediated dehydration of **8** also proceeded well to afford **3** in excellent yields (Entries 1, 2, and 5). The strong electron-withdrawing action of the nitro group in **8dc** may hamper dehydration under acidic conditions. Even under reflux conditions, the desired **3dc** was produced in only 45% yield (Entry 3). After several tests, we found that, under basic conditions (TsCl, DMAP, NEt_3_, CH_2_Cl_2_, room temperature, 3 h), **3dc** formed in 79% yield (Entry 4). The aldol/dehydration approach could serve as an additional option for synthesis of 3-(1,3-diarylallylidene)oxindoles **3** with the previous allylic oxidation/Wittig strategy.

### 2.4. Application of Stepwise Approach to An Iodoaryl Compound

**Scheme 5:**
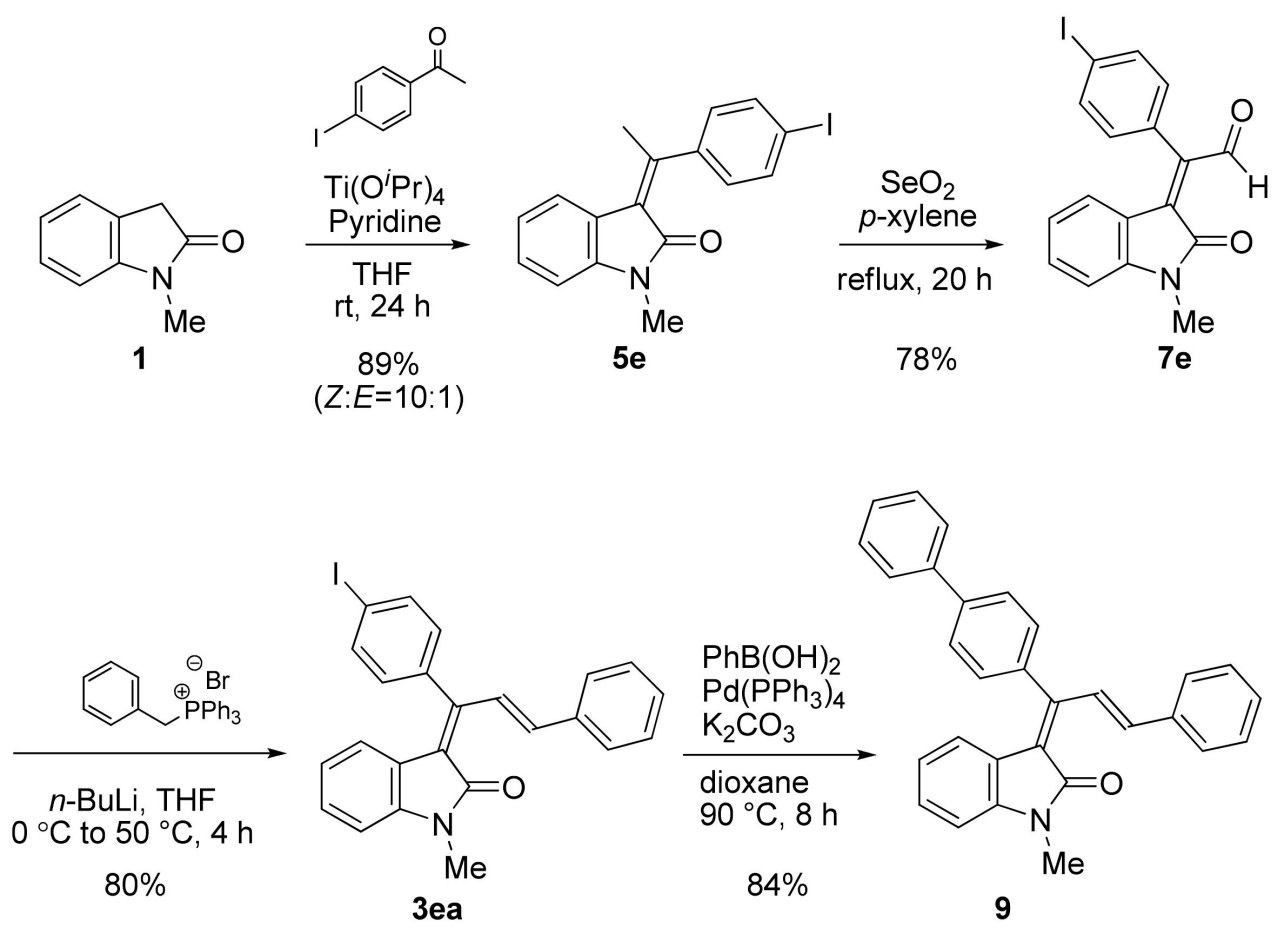
Synthesis of iodoaryl compound **3ea** and its Suzuki-Miyaura reaction.

To demonstrate that our stepwise approach is useful for synthesis of 3-(1,3-diarylallylidene)oxindoles **3**, which are not accessible by previous palladium-catalyzed methods, we synthesized **3** with an iodoaryl moiety using a Knoevenagel/allylic oxidation/Wittig strategy (Scheme 5). Due to its high reactivity toward the palladium catalyst, the iodoaryl group was not compatible with palladium-catalyzed reactions. Ti(O*^i^*Pr)_4_/pyridine-mediated Knoevenagel condensation of oxindole **1** with *p*-iodoacetophenone gave 3-methyleneoxindole **5e** in 89% yield, with a preference for the *Z*-isomer (*Z*:*E*=10:1). Allylic oxidation of **5e** afforded aldehyde **7e** in 78% yield. In addition, the last Wittig reaction of **7e** produced **3ea** (80% yield), which could not be formed using previous methods. The iodoaryl group of **3ea** could be used as a functional handle for further molecular modifications. For example, Suzuki-Miyaura reaction of **3ea** introduced another phenyl group to give **9** in 84% yield.

In conclusion, we have developed two complementary stepwise approaches to synthesize (*E*)-3-(1,3-diarylallylidene)oxindoles **3** from oxindole **1** (Knoevenagel/allylic oxidation/Wittig and Knoevenagel/aldol/dehydration). These strategies enable the synthesis of various **3**, regardless of substituents on the aryl moiety. Especially, **3** with palladium-sensitive functional groups, such as iodoaryl groups, could be obtained by these stepwise methods, which could help to expand the applications of (*E*)-3-(1,3-diarylallylidene)oxindoles.

## 3. Experimentals

### 3.1. General Information

All reactions were performed under an argon atmosphere with dry solvents, unless otherwise stated. Dry tetrahydrofuran (THF) and methylene chloride (CH_2_Cl_2_) were obtained from Ultimate Solvent Purification System (JC Meyer Solvent System, Laguna Beach, CA, USA). Other dry solvents were purchased as anhydrous grade. All glassware is oven-dried and/or flame-dried before use. All commercially available reagents were purchased and used without further purification. Reactions were monitored by thin-layer chromatography (TLC) on silica gel plates (Merck TLC Silica Gel 60 F254, Darmstadt, Germany) using UV light, PMA (an ethanolic solution of phosphomolybdic acid) or ANIS (an ethanolic solution of para-anisaldehyde) as visualizing agent. Purification of products was conducted by column chromatography through silica gel 60 (0.060−0.200 mm). Melting points of all solid compounds were determined by Buchi M-565. NMR spectra were obtained on Bruker AVANCE III 500 MHz (Bruker Corporation, Billerica, MA, USA) at 20 °C using residual undeuterated solvent or TMS (tetramethylsilane) as an internal reference. High-resolution mass spectra (HR-MS) were recorded on a JEOL JMS-700 (JEOL, Tokyo, Japan) using EI (electron impact).

### 3.2. General Procedure of Ti(O^i^Pr)_4_/pyridine Mediated Knoevenagel Condensation *(**4, 5a-e**)*

To a stirred solution of 1-methyl-2-oxindole (**1**) (1.0 mmol) and the corresponding acetophenone or chalcone (**2**) (1.0 mmol, 1.0 equiv.) in THF (10 mL) were added pyridine (0.17 mL, 2.0 mmol, 2.0 equiv.) and Ti(O*^i^*Pr)_4_ (0.90 mL, 3.0 mmol, 3.0 equiv.). The reaction mixture was stirred at rt for 24 h, Then, the mixture was diluted with EtOAc (100 mL) and 1 N aq. HCl solution (30 mL). The organic layer was separated and washed with sat. aq. NaHCO_3_ (30 mL) and brine (30 mL). The remained organic layer was dried over Na_2_SO_4_, filtered, and concentrated under reduced pressure. The crude residue was purified by column chromatography to afford Knoevenagel product **5** or 1,4-addition adduct **4**.

#### 3.2.1. 1-Methyl-3-(3-oxo-1,3-diphenylpropyl)indolin-2-one (**4**)

32% Yield; white solid; mp = 122.9–125.6 °C; *R*_f_ = 0.24 (silica gel, hexane:EtOAc = 4:1); ^1^H NMR (500 MHz, CDCl_3_): *δ* 8.09–8.01 (m, 2H), 7.57 (td, *J* = 7.4, 1.2 Hz 1H), 7.47 (dd, *J* = 10.6, 4.7 Hz, 2H), 7.33 (d, *J* = 7.3 Hz, 1H), 7.17 (t, *J* = 7.7 Hz, 1H), 7.11–7.05 (m, 5H), 7.03 (td, *J* = 7.7, 0.7 Hz, 1H), 6.57 (d, *J* = 7.7 Hz, 1H), 4.33–4.17 (m, 2H), 3.84 (d, *J* = 3.6 Hz, 1H), 3.54 (dd, *J* = 17.0, 5.1 Hz, 1H), 2.99 (s, 3H) ppm; ^13^C NMR (125 MHz, CDCl_3_) *δ* 199.1, 176.6, 144.1, 139.6, 137.2, 133.3, 128.7, 128.3, 127.98, 127.96, 127.8, 126.9, 124.4, 122.3, 107.8, 49.8, 42.2, 39.7, 25.9 ppm; HRMS (EI): calcd for C_24_H_21_NO_2_ [M^+^] : 355.1572, found 355.1573.

#### 3.2.2. (Z)-1-Methyl-3-(1-phenylethylidene)indolin-2-one (**(*Z*)-5a**)

80% Yield; yellow solid; mp = 128.9–130.2 °C; *R*_f_ = 0.45 (silica gel, hexane:EtOAc = 4:1); ^1^H NMR (500 MHz, CDCl_3_): *δ* 7.67 (d, *J* = 7.6 Hz, 1H), 7.46–7.36 (m, 3H), 7.35–7.27 (m, 3H), 7.10 (td, *J* = 7.7, 1.0 Hz, 1H), 6.84 (d, *J* = 7.7 Hz, 1H), 3.15 (s, 3H), 2.65 (s, 3H) ppm; ^13^C NMR (125 MHz, CDCl_3_) *δ* 166.4, 153.2, 143.4, 142.6, 128.6, 128.2, 128.1, 127.4, 124.0, 123.7, 123.6, 121.8, 107.8, 25.8, 25.6 ppm; HRMS (EI): calcd for C_17_H_15_NO [M^+^] : 249.1154, found 249.1151.

#### 3.2.3. (E)-1-Methyl-3-(1-phenylethylidene)indolin-2-one (**(*E*)-5a**) [19]

13% Yield; yellow solid; mp = 140.4–142.2 °C (lit. [19] 142.7-144.1 °C); *R*_f_ = 0.55 (silica gel, hexane:EtOAc = 4:1); ^1^H NMR (500 MHz, CDCl_3_): *δ* 7.51–7.41 (m, 3H), 7.28 (dt, *J* = 3.6, 2.0 Hz, 2H), 7.14 (dd, *J* = 11.2, 4.2 Hz, 1H), 6.76 (d, *J* = 7.8 Hz, 1H), 6.64 (td, *J* = 7.7, 0.8 Hz, 1H), 6.14 (d, *J* = 7.7 Hz, 1H), 3.28 (s, 3H), 2.81 (s, 3H) ppm; ^13^C NMR (125 MHz, CDCl_3_) *δ* 168.3, 155.0, 143.1, 142.4, 129.3, 128.4, 128.2, 126.6, 123.5, 122.9, 122.8, 121.5, 107.6, 29.8, 25.9, 23.0 ppm.

#### 3.2.4. (Z)-1-Methyl-3-(1-(4-chlorophenyl)ethylidene)indolin-2-one (**(*Z*)-5b**)

75% Yield; yellow solid; mp = 141.5–143.1 °C; *R*_f_ = 0.42 (silica gel, hexane:EtOAc = 4:1); ^1^H NMR (500 MHz, CDCl_3_): *δ* 7.67 (d, *J* = 7.6 Hz, 1H), 7.44–7.38 (m, 2H), 7.34 (td, *J* = 7.7, 0.9 Hz, 1H), 7.29–7.26 (m, 2H), 7.11 (td, *J* = 7.7, 0.9 Hz, 1H), 6.86 (d, *J* = 7.7 Hz, 1H), 3.17 (s, 3H), 2.64 (s, 3H) ppm; ^13^C NMR (125 MHz, CDCl_3_) *δ* 166.3, 151.5, 143.5, 140.9, 134.1, 129.0, 128.8, 128.5, 124.2, 124.1, 123.4, 122.0, 108.0, 25.8, 25.4 ppm; HRMS (EI): calcd for C_17_H_14_ClNO [M^+^] : 283.0764, found 283.0763.

#### 3.2.5. (E)-1-Methyl-3-(1-(4-chlorophenyl)ethylidene)indolin-2-one (**(*E*)-5b**)

16% Yield; yellow solid; mp = 132.1–133.9 °C; *R*_f_ = 0.43 (silica gel, hexane:EtOAc = 4:1); ^1^H NMR (500 MHz, CDCl_3_): *δ* 7.50–7.42 (m, 2H), 7.25–7.21 (m, 2H), 7.16 (t, *J* = 7.2 Hz, 1H), 6.77 (d, *J* = 7.8 Hz, 1H), 6.69 (td, *J* = 7.7, 0.9 Hz, 1H), 6.22 (d, *J* = 7.7 Hz, 1H), 3.27 (s, 3H), 2.78 (s, 3H) ppm; ^13^C NMR (125 MHz, CDCl_3_) *δ* 166.3, 151.5, 143.5, 140.9, 134.1, 129.0, 128.8, 128.5, 124.2, 124.1, 123.4, 122.0, 108.0, 25.8, 25.4 ppm; HRMS (EI): calcd for C_17_H_14_ClNO [M^+^] : 283.0764, found 283.0761.

#### 3.2.6. (Z)-1-Methyl-3-(1-(4-nitrophenyl)ethylidene)indolin-2-one (**(*Z*)-5c**)

86% Yield; orange solid; mp = 189.8–192.2 °C; *R*_f_ = 0.26 (silica gel, hexane:EtOAc = 3:1); ^1^H NMR (500 MHz, CDCl_3_): *δ* 8.28 (d, *J* = 8.5 Hz, 2H), 7.67 (d, *J* = 7.6 Hz, 1H), 7.44 (d, *J* = 8.5 Hz, 2H), 7.35 (t, *J* = 7.8 Hz, 1H), 7.12 (t, *J* = 7.7 Hz, 1H), 6.85 (d, *J* = 7.8 Hz, 1H), 3.14 (s, 3H), 2.63 (s, 3H) ppm; ^13^C NMR (125 MHz, CDCl_3_) *δ* 166.2, 149.8, 149.1, 147.4, 143.8, 129.5, 128.4, 125.1, 124.3, 123.8, 122.8, 122.3, 108.3, 25.9, 24.8 ppm; HRMS (EI): calcd for C_17_H_14_N_2_O_3_ [M^+^] : 294.1004, found 294.1001.

#### 3.2.7. (Z)-1-Methyl-3-(1-(4-methoxyphenyl)ethylidene)indolin-2-one (**(*Z*)-5d**)

62% Yield; yellow solid; mp = 125.0–127.3 °C; *R*_f_ = 0.26 (silica gel, hexane:EtOAc:CH_2_Cl_2_ = 4:1:1); ^1^H NMR (500 MHz, CDCl_3_): *δ* 7.61 (d, *J* = 7.6 Hz, 1H), 7.31 (dd, *J* = 6.4, 4.8 Hz, 2H), 7.25 (t, *J* = 7.7 Hz, 1H), 7.05 (t, *J* = 7.6 Hz, 1H), 6.92 (t, *J* = 5.6 Hz, 2H), 6.79 (d, *J* = 7.7 Hz, 1H), 3.82 (s, 3H), 3.13 (s, 3H), 2.61 (s, 3H) ppm; ^13^C NMR (125 MHz, CDCl_3_) *δ* 166.5, 160.0, 153.4, 143.1, 134.2, 129.6, 128.2, 124.0, 123.9, 123.2, 121.8, 113.5, 107.8, 55.4, 25.8, 25.7 ppm; HRMS (EI): calcd for C_18_H_17_NO_2_ [M^+^] : 279.1259, found 279.1255.

#### 3.2.8. (E)-1-Methyl-3-(1-(4-methoxyphenyl)ethylidene)indolin-2-one (**(*E*)-5d**)

15% Yield; gummy solid; *R*_f_ = 0.34 (silica gel, hexanes:EtOAc:CH_2_Cl_2_ = 4:1:1); ^1^H NMR (500 MHz, CDCl_3_): *δ* 7.26–7.22 (m, 2H), 7.14 (dd, *J* = 7.7, 7.0 Hz, 1H), 7.02–6.97 (m, 2H), 6.76 (d, *J* = 7.7 Hz, 1H), 6.67 (td, *J* = 7.7, 0.8 Hz, 1H), 6.36 (d, *J* = 7.7 Hz, 1H), 3.89 (s, 3H), 3.27 (s, 3H), 2.79 (s, 3H) ppm; ^13^C NMR (125 MHz, CDCl_3_) *δ* 168.4, 159.9, 155.2, 142.3, 135.3, 128.4, 128.0, 123.4, 123.0, 122.8, 121.4, 114.5, 107.6, 55.5, 25.9, 23.1 ppm; HRMS (EI): calcd for C_18_H_17_NO_2_ [M^+^] : 279.1259, found 279.1257.

#### 3.2.9. (Z)-1-Methyl-3-(1-(4-iodophenyl)ethylidene)indolin-2-one (**(*Z*)-5e**)

81% Yield; yellow solid; mp = 132.5–134.0 °C; *R*_f_ = 0.64 (silica gel, hexane:EtOAc:CH_2_Cl_2_ = 4:1:1); ^1^H NMR (500 MHz, CDCl_3_): *δ* 7.78–7.71 (m, 2H), 7.65 (d, *J* = 7.6 Hz, 1H), 7.32 (td, *J* = 7.7, 0.9 Hz, 1H), 7.13–7.04 (m, 3H), 6.84 (d, *J* = 7.7 Hz, 1H), 3.15 (s, 3H), 2.61 (s, 3H) ppm; ^13^C NMR (125 MHz, CDCl_3_) *δ* 166.3, 151.4, 143.5, 142.1, 137.3, 129.4, 128.9, 124.12, 124.11, 123.4, 122.0, 108.0, 94.1, 25.8, 25.3 ppm; HRMS (EI): calcd for C_17_H_14_INO [M^+^] : 375.0120, found 375.0119.

#### 3.2.10. (E)-1-Methyl-3-(1-(4-iodoxyphenyl)ethylidene)indolin-2-one (**(*E*)-5e**)

8% Yield; yellow solid; mp = 117.1–119.5 °C *R*_f_ = 0.61 (silica gel, hexane:EtOAc:CH_2_Cl_2_ = 4:1:1); ^1^H NMR (500 MHz, CDCl_3_): *δ* 7.82 (d, *J* = 8.4 Hz, 2H), 7.16 (td, *J* = 7.7, 1.0 Hz, 1H), 7.08–7.01 (m, 2H), 6.77 (d, *J* = 7.7 Hz, 1H), 6.69 (td, *J* = 7.7, 1.0 Hz, 1H), 6.24 (d, *J* = 7.7 Hz, 1H), 3.27 (s, 3H), 2.77 (s, 3H) ppm; ^13^C NMR (125 MHz, CDCl_3_) *δ* 168.1, 153.1, 142.5, 142.5, 138.5, 128.7, 128.5, 123.8, 122.9, 122.4, 121.6, 107.7, 94.2, 25.9, 22.8 ppm; HRMS (EI): calcd for C_17_H_14_INO [M^+^] : 375.0120, found 375.0120.

#### 3.3. (Z)-3-(2-Bromo-1-phenylethylidene)-1-methylindolin-2-one (**6**)

To a stirred solution of 3-methyleneoxindole **5a** (40.4 mg, 0.162 mmol) were added NBS (34.6 mg, 0.194 mmol, 1.2 equiv.) and AIBN (16 μL, 8.1 μmol, 5 mol%) in anhydrous 1,2-dichloroethane(DCE) (6 mL). The reaction mixture was refluxed under argon atmosphere for 10 h, then cooled to rt and diluted with EtOAc (50 mL) and water (50 mL). The organic layer was separated, dried over Na_2_SO_4_, filtered, and concentrated under reduced pressure. The crude residue was purified by column chromatography to afford **6** (38.1 mg, 72% yield) as a yellow solid (mp = 161.5–163.1 °C). *R*_f_ = 0.53 (silica gel, hexane:EtOAc = 3:1); ^1^H NMR (500 MHz, CDCl_3_): *δ* 7.51 (dt, *J* = 4.8, 2.5 Hz, 3H), 7.40–7.35 (m, 2H), 7.18 (td, *J* = 7.7, 1.1 Hz, 1H), 6.75 (d, *J* = 7.8 Hz, 1H), 6.64 (td, *J* = 7.7, 0.9 Hz, 1H), 6.11 (d, *J* = 7.7 Hz, 1H), 5.20 (s, 2H), 3.27 (s, 3H) ppm; ^13^C NMR (125 MHz, CDCl_3_) *δ* 167.2, 149.5, 143.4, 139.1, 129.9, 129.4, 129.3, 129.2, 127.8, 123.9, 122.0, 121.9, 108.0, 31.8, 26.0 ppm; HRMS (EI): calcd for C_17_H_14_BrNO [M^+^] : 327.0259, found 327.0254.

### 3.4. General Procedure of SeO2 Mediated Allylic Oxidation *(**7a–c, 7e**)*

To a stirred solution of the corresponding 3-methyleneoxindole **5** (0.256 mmol) in *p*-xylene (3.0 mL) was added SeO_2_ (170.4 mg, 2.0 mmol, 2.0 equiv.). The reaction mixture was refluxe for 24 h, then, the mixture was diluted with EtOAc (50 mL) and brine (30 mL). The organic layer was separated, dried over Na_2_SO_4_, filtered, and concentrated under reduced pressure. The crude residue was purified by column chromatography to afford aldehye **7**.

#### 3.4.1. (Z)-2-(1-Methyl-2-oxoindolin-3-ylidene)-2-phenylacetaldehyde (**7a**) [23]

84% Yield; orange solid; mp = 187.8–190.7 °C (lit. [23] 174-176 °C); *R*_f_ = 0.35 (silica gel, hexanes:EtOAc = 3:1); ^1^H NMR (500 MHz, CDCl_3_): *δ* 11.40 (s, 1H), 7.49 (dd, *J* = 3.7, 2.7 Hz, 3H), 7.28–7.19 (m, 3H), 6.77 (d, *J* = 7.8 Hz, 1H), 6.68 (td, *J* = 7.7, 0.9 Hz, 1H), 6.26 (d, *J* = 7.7 Hz, 1H), 3.25 (s, 3H) ppm; ^13^C NMR (125 MHz, CDCl_3_) *δ* 193.3, 167.3, 145.8, 145.6, 135.5, 133.3, 132.2, 129.4, 129.2, 128.9, 126.2, 122.6, 121.4, 108.6, 26.2 ppm; HRMS (EI): calcd for C_17_H_13_NO_2_ [M^+^] : 263.0946, found 263.0942.

#### 3.4.2. (Z)-2-(4-Chlorophenyl)-2-(1-methyl-2-oxoindolin-3-ylidene)acetaldehyde (**7b**)

85% Yield; orange solid; mp = 186.8–189.1 °C; *R*_f_ = 0.32 (silica gel, hexane:EtOAc = 3:1); ^1^H NMR (500 MHz, CDCl_3_): *δ* 11.38 (s, 1H), 7.52–7.45 (m, 2H), 7.29 (td, *J* = 7.8, 1.1 Hz, 1H), 7.20–7.16 (m, 2H), 6.79 (d, *J* = 7.8 Hz, 1H), 6.74 (td, *J* = 7.7, 0.9 Hz, 1H), 6.36 (d, *J* = 7.4 Hz, 1H), 3.27 (s, 3H) ppm; ^13^C NMR (125 MHz, CDCl_3_) *δ* 192.9, 167.1, 145.7, 144.4, 135.9, 135.6, 132.6, 131.6, 130.6, 129.6, 126.1, 122.7, 121.1, 108.8, 26.3 ppm; HRMS (EI): calcd for C_17_H_12_ClNO_2_ [M^+^] : 297.0557, found 297.0556.

#### 3.4.3. (Z)-2-(1-Methyl-2-oxoindolin-3-ylidene)-2-(4-nitrophenyl)acetaldehyde (**7c**)

87% Yield; red solid; mp = 194.2–196.8 °C; *R*_f_ = 0.34 (silica gel, hexane:EtOAc = 4:1); ^1^H NMR (500 MHz, CDCl_3_): *δ* 11.41 (s, 1H), 8.36 (d, *J* = 8.5 Hz, 2H), 7.43 (d, *J* = 8.5 Hz, 2H), 7.31 (t, *J* = 7.7 Hz, 1H), 6.81 (d, *J* = 7.8 Hz, 1H), 6.71 (t, *J* = 7.7 Hz, 1H), 6.20 (d, *J* = 7.7 Hz, 1H), 3.27 (s, 3H) ppm; ^13^C NMR (125 MHz, CDCl_3_) *δ* 192.1, 166.8, 148.4, 146.1, 142.9, 140.2, 136.4, 133.2, 130.4, 126.1, 124.4, 122.9, 120.6, 109.1, 26.3 ppm; HRMS (EI): calcd for C_17_H_12_N_2_O_4_ [M^+^] : 308.0797, found 308.0797.

#### 3.4.4. (Z)-2-(4-Iodophenyl)-2-(1-methyl-2-oxoindolin-3-ylidene)acetaldehyde (**7e**)

78% Yield; orange solid; mp = 157.2–159.5 °C; *R*_f_ = 0.20 (silica gel, hexane:EtOAc = 3:1); ^1^H NMR (500 MHz, CDCl_3_): *δ* 11.36 (s, 1H), 7.83 (d, *J* = 8.2 Hz, 2H), 7.29 (td, *J* = 7.8, 0.8 Hz, 1H), 6.98 (d, *J* = 8.2 Hz, 2H), 6.78 (d, *J* = 7.8 Hz, 1H), 6.74 (t, *J* = 7.7 Hz, 1H), 6.38 (d, *J* = 7.7 Hz, 1H), 3.25 (s, 3H) ppm; ^13^C NMR (125 MHz, CDCl_3_) *δ* 192.7, 167.0, 145.7, 144.3, 138.3, 135.7, 132.7, 132.6, 130.9, 126.1, 122.7, 121.0, 108.7, 95.7, 26.2 ppm; HRMS (EI): calcd for C_17_H_12_INO_2_ [M^+^] : 388.9913, found 388.9908.

### 3.5. General Procedure of Wittig Reaction *(**3aa-ad, 3ba-bd, 3ca-cd, 3ea**)*

To a stirred solution of the corresponding benzyltriphenylphosphonium bromide (0.098 mmol, 1.5 equiv.) in THF (2.0 mL) was slowly added *n*-butyllithium (2.5 M in hexane, 0.036 mL, 0.091 mmol, 1.4 equiv.) at 0 °C under argon atmosphere. After stirring for 1 h at the same temperature, a solution of the corresponding aldehyde **7** (0.065 mmol, 1.0 equiv.) in THF (1.0 mL) was added. Then, the reaction temperature was raised to 50 °C. After 4 h, the reaction mixture was cooled to rt and diluted with sat. aq. NH_4_Cl (30 mL) and EtOAc (70 mL). The organic layer was separated, dried over Na_2_SO_4_, filtered, and concentrated under reduced pressure. The crude residue was purified by column chromatography to afford 3-(1,3-diarylallyliden)oxindole **3**.

#### 3.5.1. (E)-3-((E)-1,3-Diphenylallylidene)-1-methylindolin-2-one (**3aa**)

84% Yield; yellow solid; mp = 125.7 °C; *R*_f_ = 0.4 (silica gel, hexane:EtOAc = 4:1); ^1^H NMR (500 MHz, CDCl_3_): *δ* 9.38 (d, *J* = 16.0 Hz, 1H), 7.55–7.51 (m, 5H), 7.33–7.25 (m, 5H), 7.08 (td, *J* = 8.2, 7.6 Hz, 1H), 6.74 (d, *J* = 7.8 Hz, 1H), 6.60 (t, *J* = 7.7 Hz, 1H), 6.47 (d, *J* = 16.0 Hz, 1H), 5.72 (d, *J* = 7.8 Hz, 1H), 3.3 (s, 3H) ppm; ^13^C NMR (125 MHz, CDCl_3_): *δ* 168.1, 151.2, 142.9, 141.6, 137.8, 136.9, 129.3, 129.2, 128.8, 128.7, 128.6, 128.3, 128.0, 127.7, 123.7, 123.4, 122.6, 121.6, 107.6, 25.9 ppm; HRMS (EI): calcd for C_24_H_19_NO [M^+^]: 337.1467, found 337.1466.

#### 3.5.2. (E)-3-((E)-3-(4-Chlorophenyl)-1-phenylallylidene)-1-methylindolin-2-one (**3ab**)

67% Yield; yellow solid; mp = 123.2 °C; *R*_f_ = 0.44 (silica gel, hexane:EtOAc = 3:1); ^1^H NMR (500 MHz, CDCl_3_): *δ* 9.36 (d, *J* = 16 Hz, 1H), 7.57–7.53 (m, 3H), 7.44 (dt, *J* = 13.3, 2.3 Hz, 2H), 7.29–7.26 (m, 4H), 7.10 (td, *J* = 7.7, 1.1 Hz, 1H), 6.75 (d, *J* = 7.7 Hz, 1H), 6.59 (td, *J* = 7.7, 1.0 Hz, 1H), 6.40 (d, *J* = 16.0 Hz, 1H), 5.72 (d, *J* = 7.8 Hz, 1H), 3.30 (s, 3H) ppm; ^13^C NMR (125 MHz, CDCl_3_): *δ* 168.1, 150.8, 143.0, 139.9, 137.5, 135.5, 134.8, 129.4, 129.1, 129.0, 128.7, 128.6, 128.5, 128.2, 123.8, 123.3, 123.0, 121.7, 107.7, 25.9 ppm; HRMS (EI): calcd for C_24_H_18_ClNO [M^+^]: 371.1077, found 371.1077.

#### 3.5.3. (E)-1-Methyl-3-((E)-3-(4-nitrophenyl)-1-phenylallylidene)indolin-2-one (**3ac**)

64% Yield; red solid; mp = 183.3–184.7 °C; *R*_f_ = 0.32 (silica gel, hexane:EtOAc = 8:1); ^1^H NMR (500 MHz, CDCl_3_): *δ* 9.52 (d, *J* = 16.0 Hz, 1H), 8.22–8.10 (m, 2H), 7.64 (d, *J* = 8.8 Hz, 2H), 7.61–7.53 (m, 3H), 7.31–7.26 (m, 2H), 7.15 (td, *J* = 7.7, 1.1 Hz, 1H), 6.76 (d, *J* = 7.7 Hz, 1H), 6.62 (td, *J* = 7.7, 0.9 Hz, 1H), 6.46 (d, *J* = 16.0 Hz, 1H), 5.76 (d, *J* = 7.5 Hz, 1H), 3.30 (s, 3H) ppm; ^13^C NMR (125 MHz, CDCl_3_): *δ* 168.0, 149.5, 147.5, 143.4, 137.9, 137.0, 131.8, 129.6, 129.3, 128.9, 128.6, 128.3, 124.9, 124.21, 124.16, 123.0, 121.9, 107.9, 25.9 ppm; HRMS (EI): calcd for C_24_H_18_N_2_O_3_ [M^+^] : 382.1317, found 382.1316.

#### 3.5.4. (E)-3-((E)-3-(4-Methoxyphenyl)-1-phenylallylidene)-1-methylindolin-2-one (**3ad**)

53% Yield; yellow solid; mp = 135.5 °C; *R*_f_ = 0.2 (silica gel, hexane:EtOAc = 5:1); ^1^H NMR (500 MHz, CDCl_3_): *δ* 9.27 (d, *J* = 16.0 Hz, 1H), 7.55–7.52 (m, 3H), 7.48 (d, *J* = 8.7 Hz, 2H), 7.28–7.27 (m, 2H), 7.08 (td, *J* = 7.7, 1.1 Hz, 1H), 6.84 (dt, *J* = 14.3, 2.9 Hz, 2H), 6.75 (d, *J* = 7.7 Hz, 1H), 6.59 (td, *J* = 7.7, 1.0 Hz, 1H), 6.44 (d, *J* = 16 Hz, 1H), 5.70 (d, *J* = 7.7 Hz, 1H), 3.82 (s, 3H), 3.30(s, 3H) ppm; ^13^C NMR (125 MHz, CDCl_3_): *δ* 168.2, 160.7, 151.9, 142.7, 141.5, 137.9, 129.9, 129.6, 129.3, 128.7, 128.5, 128.0, 125.7, 123.6, 123.5, 121.5, 114.3, 107.5, 55.5, 25.9 ppm; HRMS (EI): calcd for C_25_H_21_NO_2_ [M^+^]: 367.1572, found 367.1572.

#### 3.5.5. (E)-3-((E)-1-(4-Chlorophenyl)-3-phenylallylidene)-1-methylindolin-2-one (**3ba**)

95% Yield; yellow solid; mp = 178.2 °C; *R*_f_ = 0.3 (silica gel, hexane:EtOAc = 6:1); ^1^H NMR (500 MHz, CDCl_3_): *δ* 9.37 (d, *J* = 16.1 Hz, 1H), 7.56–7.53 (m, 4H), 7.33 (t, *J* = 7.3 Hz, 2H), 7.29 (d, *J* = 7.1 Hz, 1H), 7.24 (dt, *J* = 12.8, 2.2 Hz, 2H), 7.13 (td, *J* = 7.7, 1.0 Hz, 1H), 6.76 (d, *J* = 7.7 Hz, 1H), 6.66 (td, *J* = 7.7, 0.9 Hz, 1H), 6.43 (d, *J* = 16.1 Hz, 1H), 5.86 (d, *J* = 7.7 Hz, 1H), 3.29 (s, 3H) ppm; ^13^C NMR (125 MHz, CDCl_3_): *δ* 167.9, 149.6, 143.0, 141.4, 136.8, 136.1, 134.6, 130.3, 129.7, 129.3, 128.9, 128.6, 128.0, 127.5, 123.5, 123.1, 122.8, 121.7, 107.8, 25.9 ppm; HRMS (EI): calcd for C_24_H_18_ClNO [M^+^]: 371.1077, found 371.1078.

#### 3.5.6. (E)-3-((E)-1,3-Bis(4-chlorophenyl)allylidene)-1-methylindolin-2-one (**3bb**)

79% Yield; yellow solid; mp = 193.0 °C; *R*_f_ = 0.27 (silica gel, hexane:EtOAc = 8:1); ^1^H NMR (500 MHz, CDCl_3_): *δ* 9.33 (d, *J* = 16.1 Hz, 1H), 7.55 (d, *J* = 8.3 Hz, 2H), 7.44 (d, *J* = 8.4 Hz, 2H), 7.28 (d, *J* = 8.4 Hz, 2H), 7.23 (d, *J* = 8.3 Hz, 2H), 7.15 (t, *J* = 7.7 Hz, 1H), 6.76 (d, *J* = 7.8 Hz, 1H), 6.67 (t, *J* = 7.7 Hz, 1H), 6.35 (d, *J* = 16.1 Hz, 1H), 5.86 (d, *J* = 7.7 Hz, 1H), 3.28 (s, 3H) ppm; ^13^C NMR (125 MHz, CDCl_3_): *δ* 167.9, 167.2, 163.1, 139.7, 135.9, 135.3, 135.0, 134.8, 130.3, 129.8, 129.1, 128.8, 128.0, 123.7, 123.2, 123.0, 121.8, 107.8, 25.9 ppm; HRMS (EI): calcd for C_24_H_17_Cl_2_NO [M^+^]: 405.0687, found 405.0689.

#### 3.5.7. (E)-3-((E)-1-(4-Chlorophenyl)-3-(4-nitrophenyl)allylidene)-1-methylindolin-2-one (**3bc**)

82% Yield; red solid; mp = >250 °C; *R*_f_ = 0.50 (silica gel, hexane:EtOAc:CH_2_Cl_2_ = 4:1:1); ^1^H NMR (500 MHz, CDCl_3_): *δ* 9.53 (d, *J* = 16.2 Hz, 1H), 8.47 (d, *J* = 8.1 Hz, 2H), 8.18 (d, *J* = 8.3 Hz, 2H), 7.63 (d, *J* = 8.3 Hz, 2H), 7.53 (d, *J* = 8.1 Hz, 2H), 7.20 (t, *J* = 7.6 Hz, 1H), 6.80 (d, *J* = 7.6 Hz, 1H), 6.66 (t, *J* = 7.6 Hz, 1H), 6.30 (d, *J* = 16.2 Hz, 1H), 5.74 (d, *J* = 7.6 Hz, 1H), 3.31 (s, 3H) ppm; ^13^C NMR (125 MHz, CDCl_3_): *δ* 167.5, 148.3, 147.8, 146.3, 143.9, 143.7, 142.8, 137.8, 130.8, 130.1, 128.3, 125.2, 125.0, 124.3, 123.8, 122.19, 122.15, 108.4, 26.1 ppm; HRMS (EI): calcd for C_24_H_17_N_3_O_5_ [M^+^] : 427.1168, found 427.1169.

#### 3.5.8. (E)-3-((E)-1-(4-Chlorophenyl)-3-(4-methoxyphenyl)allylidene)-1-methylindolin-2-one (**3bd**)

68% Yield; yellow solid; mp = 129.0 °C; *R*_f_ = 0.38 (silica gel, CH_2_Cl_2_:hexane 5:1); ^1^H NMR (500 MHz, CDCl_3_): *δ* 9.24 (d, *J* = 16 Hz, 1H), 7.53 (dt, *J* = 13.1, 2.2 Hz, 2H), 7.48 (d, *J* = 8.7 Hz, 2H), 7.22 (dt, *J* = 12.6, 2.1 Hz, 2H), 7.11 (td, *J* = 7.4, 1.1 Hz, 1H), 6.85 (dt, *J* = 14.4, 2.4 Hz, 2H), 6.76 (d, *J* = 7.7 Hz, 1H), 6.64 (td, *J* = 7.7, 1.0, 1H), 6.38 (d, *J* = 16 Hz, 1H), 5.83 (d, *J* = 7.5 Hz, 1H), 3.82 (s, 3H), 3.30 (s, 3H) ppm; ^13^C NMR (125 MHz, CDCl_3_): *δ* 168.0, 160.8, 150.2, 142.8, 141.4, 136.4, 134.5, 130.3, 129.73, 129.65, 129.6, 128.3, 125.5, 123.3, 121.6, 114.4, 107.7, 55.5, 25.9. ppm; HRMS (EI): calcd for C_25_H_20_ClNO_2_ [M^+^]: 401.1183, found 401.1185.

#### 3.5.9. (E)-1-Methyl-3-((E)-1-(4-nitrophenyl)-3-phenylallylidene)indolin-2-one (**3ca**)

80% Yield; yellow solid; mp = 228.2 °C; *R*_f_ = 0.3 (silica gel, hexanes:EtOAc = 3:1); ^1^H NMR (500 MHz, CDCl_3_): *δ* 9.39 (d, *J* = 16.2 Hz, 1H), 8.45 (d, *J* = 8.8 Hz, 2H), 7.53–7.50 (m, 4H), 7.35–7.30 (m, 3H), 7.15 (td, *J* = 7.7, 1.1 Hz, 1H), 6.78 (d, *J* = 7.7 Hz, 1H), 6.62 (td, *J* = 7.7, 1.0 Hz, 1H), 6.30 (d, *J* = 16.2 Hz, 1H), 5.70 (d, *J* = 7.5 Hz, 1H), 3.31 (s, 3H) ppm; ^13^C NMR (125 MHz, CDCl_3_): *δ* 167.7, 148.1, 148.0, 144.7, 143.2, 141.5, 136.5, 130.2, 129.6, 129.2, 123.0, 128.0, 126.8, 124.8, 123.3, 122.9, 122.6, 121.9, 108.1, 26.0 ppm; HRMS (EI): calcd for C_24_H_18_N_2_O_3_ [M^+^]: 382.1317, found 382.1318.

#### 3.5.10. (E)-3-((E)-3-(4-Chlorophenyl)-1-(4-nitrophenyl)allylidene)-1-methylindolin-2-one (**3cb**)

79% Yield; orange solid; mp = 249.5 °C; *R*_f_ = 0.34 (silica gel, hexanes:EtOAc = 5:1); ^1^H NMR (500 MHz, CDCl_3_): *δ* 9.37 (d, *J* = 16.2 Hz, 1H), 8.44 (dt, *J* = 12.9, 2.3 Hz, 2H), 7.50 (dt, *J* = 13.0, 2.3 Hz, 2H), 7.43 (d, *J* = 8.5 Hz, 2H), 7.30 (d, *J* = 8.6 Hz, 2H), 7.15 (td, *J* = 7.7, 0.9 Hz, 1H), 6.78 (d, *J* = 7.8 Hz, 1H), 6.62 (td, *J* = 7.7, 0.8 Hz, 1H), 6.23 (d, *J* = 16.2 Hz, 1H), 5.71 (d, *J* = 7.7 Hz, 1H), 3.30 (s, 3H) ppm; ^13^C NMR (125 MHz, CDCl_3_): *δ* 167.6, 148.2, 147.5, 144.4, 143.3, 139.8, 135.4, 135.0, 130.2, 129.4, 129.3, 129.2, 129.1, 128.3, 127.3, 127.1, 124.8, 124.3, 123.4, 123.3, 122.5, 122.0, 108.2, 26.0 ppm; HRMS (EI): calcd for C_24_H_17_ClN_2_O_3_ [M^+^]: 416.0928, found 416.0928.

#### 3.5.11. (E)-3-((E)-1,3-Bis(4-nitrophenyl)allylidene)-1-methylindolin-2-one (**3cc**)

68% Yield; orange solid; mp = 243.5–245.2 °C; *R*_f_ = 0.38 (silica gel, hexanes:EtOAc = 5:1); ^1^H NMR (500 MHz, CDCl_3_): *δ* 9.49 (d, *J* = 16.1 Hz, 1H), 8.16 (d, *J* = 8.7 Hz, 2H), 7.63 (d, *J* = 8.7 Hz, 2H), 7.57 (d, *J* = 8.2 Hz, 2H), 7.24 (d, *J* = 8.2 Hz, 2H), 7.17 (d, *J* = 7.6 Hz, 1H), 6.77 (d, *J* = 7.7 Hz, 1H), 6.68 (t, *J* = 7.6 Hz, 1H), 6.41 (d, *J* = 16.1 Hz, 1H), 5.88 (d, *J* = 7.7 Hz, 1H), 3.29 (s, 3H) ppm; ^13^C NMR (125 MHz, CDCl_3_): *δ* 167.8, 148.0, 147.6, 143.5, 143.1, 137.8, 135.4, 135.0, 131.5, 130.2, 130.0, 129.6, 128.3, 125.1, 124.2, 124.1, 122.7, 122.1, 108.1, 26.0 ppm; HRMS (EI): calcd for C_24_H_17_ClN_2_O_3_ [M^+^] : 416.0928, found 416.0923.

#### 3.5.12. (E)-3-((E)-3-(4-Methoxyphenyl)-1-(4-nitrophenyl)allylidene)-1-methylindolin-2-one (**3cd**)

82% Yield; orange solid; mp = 197.0 °C; *R*_f_ = 0.23 (silica gel, hexanes:EtOAc = 4:1); ^1^H NMR (500 MHz, CDCl_3_): *δ* 9.27 (d, *J* = 16.2 Hz, 1H), 8.43 (dt, *J* = 13.0, 2.3 Hz, 2H), 7.50 (dt, *J* = 13, 2.3 Hz, 2H), 7.46 (d, *J* = 8.8 Hz, 2H), 7.13 (td, *J* = 7.7, 0.9 Hz, 1H), 6.9 (d, *J* = 8.8 Hz, 2H), 6.78 (d, *J* = 7.8 Hz, 1H), 6.60 (td, *J* = 7.7, 0.8 Hz, 1H), 6.26 (d, *J* = 16.2 Hz, 1H), 5.68 (,d *J* = 7.7 Hz, 1H), 3.82 (s, 3H), 3.30 (s, 3H) ppm; ^13^C NMR (125 MHz, CDCl_3_): *δ* 167.8, 161.1, 148.6, 148.1, 144.9, 143.0, 141.4, 130.2, 129.7, 129.4, 128.8, 124.8, 124.7, 123.1, 122.8, 121.8, 121.7, 114.5, 108.0, 55.5, 25.9 ppm; HRMS (EI): calcd for C_25_H_20_N_2_O_4_ [M^+^]: 412.1423, found 412.1422.

#### 3.5.13. (E)-3-((E)-1-(4-Iodophenyl)-3-phenylallylidene)-1-methylindolin-2-one (**3ea**)

80% Yield; orange solid; mp = 182.7–184.0 °C; *R*_f_ = 0.31 (silica gel, hexanes:EtOAc = 6:1); ^1^H NMR (500 MHz, CDCl_3_): *δ* 9.35 (d, *J* = 16.1 Hz, 1H), 7.93–7.85 (m, 2H), 7.56–7.50 (m, 2H), 7.36–7.31 (m, 2H), 7.31–7.27 (m, 1H), 7.15 (td, *J* = 7.7, 1.1 Hz, 1H), 7.07–7.02 (m, 2H), 6.76 (d, *J* = 7.7 Hz, 1H), 6.68 (td, *J* = 7.7, 1.0 Hz, 1H), 6.42 (d, *J* = 16.1 Hz, 1H), 5.86 (d, *J* = 7.4 Hz, 1H), 3.30 (s, 3H) ppm; ^13^C NMR (125 MHz, CDCl_3_): *δ* 167.9, 149.7, 143.0, 141.5, 138.6, 137.2, 136.8, 130.8, 129.3, 128.9, 128.7, 128.0, 127.3, 123.6, 123.1, 122.7, 121.8, 107.8, 94.5, 25.9 ppm; HRMS (EI): calcd for C_24_H_18_INO [M^+^] : 463.0433, found 463.0430.

### 3.6. General Procedure of Aldol Reaction *(**8aa**, **8da-dd**)*

To a stirred solution of the corresponding 3-methyleneoxindole **5** (0.20 mmol) in THF (2.0 mL) was slowly added *n*-butyllithium (2.5 M in hexane, 0.088 mL, 0.22 mmol, 1.1 equiv.) at −78 °C under argon atmosphere. After stirring for 1 h at the same temperature, the corresponding benzaldehyde (0.26 mmol, 1.3 equiv.) was added. After stirring for additional 1.5 h at −78 °C, the reaction mixture was diluted with sat. aq. NH_4_Cl (10 mL) and EtOAc (50 mL). The organic layer was separated, dried over Na_2_SO_4_, filtered, and concentrated under reduced pressure. The crude residue was purified by column chromatography to afford aldol adduct **8**.

#### 3.6.1. (E)-3-(3-Hydroxy-1,3-diphenylpropylidene)-1-methylindolin-2-one (**8aa**)

91% Yield; yellow foam; *R*_f_ = 0.37 (silica gel, hexane:EtOAc = 2:1); ^1^H NMR (500 MHz, CDCl_3_): *δ* 7.57–7.43 (m, 4H), 7.40 (d, *J* = 7.4 Hz, 2H), 7.31 (t, *J* = 7.5 Hz, 2H), 7.27–7.19 (m, 2H), 7.15 (t, *J* = 7.6 Hz, 1H), 6.79 (d, *J* = 7.7 Hz, 1H), 6.66 (t, *J* = 7.6 Hz, 1H), 6.14 (d, *J* = 7.7 Hz, 1H), 4.82 (d, *J* = 9.8 Hz, 1H), 4.50 (s, 1H), 4.42 (dd, *J* = 12.6, 11.0 Hz, 1H), 3.30 (s, 3H), 2.88 (dd, *J* = 12.8, 2.8 Hz, 1H) ppm; ^13^C NMR (125 MHz, CDCl_3_): *δ* 169.6, 155.6, 145.5, 142.3, 141.3, 129.6, 129.1, 128.9, 128.7, 128.5, 127.3, 126.5, 126.3, 125.7, 123.0, 122.8, 122.1, 108.0, 73.7, 46.0, 26.2 ppm; HRMS (EI): calcd for C_24_H_21_NO_2_ [M^+^] : 355.1572, found 355.1568.

#### 3.6.2. (E)-3-(3-Hydroxy-1-(4-methoxyphenyl)-3-phenylpropylidene)-1-methylindolin-2-one (**8da**)

93% Yield; yellow foam; *R*_f_ = 0.18 (silica gel, hexane:EtOAc:CH_2_Cl_2_ = 4:1:1); ^1^H NMR (500 MHz, CDCl_3_): *δ* 7.42 (dd, *J* = 13.8, 7.3 Hz, 3H), 7.33 (dd, *J* = 10.4, 4.8 Hz, 2H), 7.26–7.19 (m, 2H), 7.19–7.13 (m, 1H), 7.09–6.96 (m, 2H), 6.81 (d, *J* = 7.7 Hz, 1H), 6.71 (td, *J* = 7.7, 1.0 Hz, 1H), 6.38 (d, *J* = 7.4 Hz, 1H), 4.82–4.73 (m, 1H), 4.65 (d, *J* = 7.7 Hz, 1H), 4.39 (dd, *J* = 12.8, 10.8 Hz, 1H), 3.92 (s, 3H), 3.32 (s, 3H), 2.88 (dd, *J* = 12.8, 2.9 Hz, 1H) ppm; ^13^C NMR (125 MHz, CDCl_3_): *δ* 169.8, 160.4, 156.1, 145.7, 142.2, 133.2, 130.7, 128.53, 128.48, 128.0, 127.3, 126.3, 125.7, 123.1, 122.8, 122.1, 115.1, 114.3, 108.1, 74.1, 55.5, 46.3, 26.2 ppm; HRMS (EI): calcd for C_25_H_21_NO_2_ [M-H_2_O]^+^ : 367.1572, found 367.1567.

#### 3.6.3. (E)-3-(3-(4-Chlorophenyl)-3-hydroxy-1-(4-methoxyphenyl)propylidene)-1-methylindolin-2-one (**8db**)

75% Yield; orange foam; *R*_f_ = 0.38 (silica gel, hexane:EtOAc:CH_2_Cl_2_ = 4:1:1); ^1^H NMR (500 MHz, CDCl_3_): *δ* 7.39 (d, *J* = 7.8 Hz, 1H), 7.35–7.22 (m, 4H), 7.18 (dd, *J* = 15.7, 8.0 Hz, 2H), 7.00 (dd, *J* = 22.8, 8.2 Hz, 2H), 6.80 (d, *J* = 7.7 Hz, 1H), 6.71 (t, *J* = 7.6 Hz, 1H), 6.38 (d, *J* = 7.7 Hz, 1H), 4.78 (s, 2H), 4.32–4.19 (m, 1H), 3.90 (s, 3H), 3.30 (s, 3H), 2.97–2.88 (m, 1H) ppm; ^13^C NMR (125 MHz, CDCl_3_): *δ* 169.7, 160.4, 155.6, 144.1, 142.2, 133.0, 132.8, 130.5, 128.6, 128.5, 128.1, 127.1, 126.4, 123.0, 122.8, 122.2, 115.0, 114.3, 108.1, 73.4, 55.5, 46.2, 26.2 ppm; HRMS (EI): calcd for C_25_H_20_ClNO_2_ [M-H_2_O]^+^ : 401.1183, found 401.1183.

#### 3.6.4. (E)-3-(3-Hydroxy-1-(4-methoxyphenyl)-3-(4-nitrophenyl)propylidene)-1-methylindolin-2-one (**8dc**)

86% Yield; orange foam; *R*_f_ = 0.20 (silica gel, hexane:EtOAc:CH_2_Cl_2_ = 4:1:1); ^1^H NMR (500 MHz, CDCl_3_): *δ* 8.14 (d, *J* = 8.5 Hz, 2H), 7.54 (d, *J* = 8.4 Hz, 2H), 7.39 (d, *J* = 7.7 Hz, 1H), 7.19 (t, *J* = 7.5 Hz, 2H), 7.00 (t, *J* = 9.6 Hz, 2H), 6.82 (d, *J* = 7.7 Hz, 1H), 6.73 (t, *J* = 7.6 Hz, 1H), 6.41 (d, *J* = 7.7 Hz, 1H), 5.18 (d, *J* = 6.7 Hz, 1H), 5.00–4.86 (m, 1H), 4.17 (dd, *J* = 12.7, 10.4 Hz, 1H), 3.90 (s, 3H), 3.32 (s, 3H), 3.05 (dd, *J* = 12.8, 2.5 Hz, 1H) ppm; ^13^C NMR (125 MHz, CDCl_3_): *δ* 169.8, 160.6, 154.7, 153.0, 147.1, 142.2, 132.8, 130.5, 128.8, 128.3, 126.8, 126.5, 123.7, 122.83, 122.81, 122.4, 115.0, 114.5, 108.3, 73.4, 55.5, 45.9, 26.3 ppm; HRMS (EI): calcd for C_25_H_20_N_2_O_4_ [M-H_2_O]^+^ : 412.1423, found 412.1421.

#### 3.6.5. (E)-3-(3-Hydroxy-1,3-bis(4-methoxyphenyl)propylidene)-1-methylindolin-2-one (**8dd**)

63% Yield; orange foam; *R*_f_ = 0.14 (silica gel, hexane:EtOAc:CH_2_Cl_2_ = 4:1:1); ^1^H NMR (500 MHz, CDCl_3_): *δ* 7.41 (d, *J* = 8.0 Hz, 1H), 7.33 (d, *J* = 8.6 Hz, 2H), 7.21 (d, *J* = 7.7 Hz, 1H), 7.16 (t, *J* = 7.4 Hz, 1H), 7.02 (dd, *J* = 25.1, 8.3 Hz, 2H), 6.86 (d, *J* = 8.6 Hz, 2H), 6.79 (d, *J* = 7.7 Hz, 1H), 6.71 (t, *J* = 7.6 Hz, 1H), 6.37 (d, *J* = 7.7 Hz, 1H), 4.73 (d, *J* = 9.8 Hz, 1H), 4.48 (s, 1H), 4.37 (dd, *J* = 12.7, 10.7 Hz, 1H), 3.91 (s, 3H), 3.79 (s, 3H), 3.31 (s, 3H), 2.90 (dd, *J* = 12.8, 3.0 Hz, 1H) ppm; ^13^C NMR (125 MHz, CDCl_3_): *δ* 169.7, 160.3, 158.9, 156.2, 142.2, 137.9, 133.2, 130.6, 128.5, 128.1, 126.9, 126.2, 123.1, 122.8, 122.1, 115.0, 114.3, 113.9, 108.0, 73.6, 55.5, 55.4, 46.2, 26.2 ppm; HRMS (EI): calcd for C_26_H_23_NO_3_ [M-H_2_O]^+^ : 397.1678, found 397.1678.

### 3.7. General Procedure of Dehydration under Acidic Conditions *(**3aa, 3da-dd**)*

To a stirred solution of the corresponding alcohol **8** (0.20 mmol) in CH_2_Cl_2_ (2.4 mL) was added trifluoroacetic acid (TFA) (0.3 mL) at rt. After 1 h, volatile material was distilled off under reduced pressure. The crude residue was purified by column chromatography to afford 3-(1,3-diarylallyliden)oxindole **3**.

#### 3.7.1. (E)-3-((E)-1,3-Diphenylallylidene)-1-methylindolin-2-one (**3aa**)

95% Yield.

#### 3.7.2. (E)-3-((E)-1-(4-Methoxyphenyl)-3-phenylallylidene)-1-methylindolin-2-one (**3da**)

90% Yield; yellow solid; mp = 153.6 °C; *R*_f_ = 0.3 (silica gel, hexane:EtOAc = 4:1); ^1^H NMR (500 MHz, CDCl_3_): *δ* 9.36 (d, *J* = 16 Hz, 1H), 7.55 (d, *J* = 7.4 Hz, 2H), 7.32 (t, *J* = 7.4 Hz, 2H), 7.27 (d, *J* = 5.8 Hz, 1H), 7.21 (d, *J* = 8.5 Hz, 2H), 7.12 (t, *J* = 7.6 Hz, 1H), 7.08 (d, *J* = 8.5 Hz, 2H), 6.75 (d, *J* = 7.7 Hz, 1H), 6.65 (t, *J* = 7.6 Hz, 1H), 6.53 (d, *J* = 16 Hz, 1H), 5.92 (d, *J* = 7.7 Hz, 1H), 3.94 (s, 3H), 3.30 (s, 3H) ppm; ^13^C NMR (125 MHz, CDCl_3_): *δ* 168.1, 159.8, 151.2, 142.8, 141.4, 137.0, 130.1, 129.9, 129.1, 128.8, 128.21, 128.19, 128.0, 123.64, 123.58, 122.8, 121.6, 114.7, 107.6, 55.5, 25.8 ppm; HRMS (EI): calcd for C_25_H_21_NO_2_ [M^+^]: 367.1572, found 367.1571.

#### 3.7.3. (E)-3-((E)-3-(4-Chlorophenyl)-1-(4-methoxyphenyl)allylidene)-1-methylindolin-2-one (**3db**)

93% Yield; yellow solid; mp = 143.1 °C; *R*_f_ = 0.36 (silica gel, hexane:EtOAc = 5:1); ^1^H NMR (500 MHz, CDCl_3_): *δ* 9.31 (d, *J* = 16.0 Hz, 1H), 7.45 (d, *J* = 8.5 Hz, 2H), 7.27 (d, *J* = 8.6 Hz, 2H), 7.19 (d, *J* = 8.6 Hz, 2H), 7.12 (t, *J* = 7.7 Hz, 1H), 7.07 (d, *J* = 8.6 Hz, 2H), 6.74 (d, *J* = 7.8 Hz, 1H), 6.65 (t, *J* = 7.8 Hz, 1H), 6.45 (d, *J* = 16.0 Hz, 1H), 5.92 (d, *J* = 7.7 Hz, 1H), 3.93 (s, 3H), 3.28 (s, 3H) ppm; ^13^C NMR (125 MHz, CDCl_3_): *δ* 168.1, 159.9, 150.7, 142.9, 139.7, 135.6, 134.7, 130.1, 129.6, 129.04, 128.97, 128.7, 128.4, 123.7, 123.5, 123.2, 121.6, 114.7, 107.6, 55.5, 25.8 ppm; HRMS (EI): calcd for C_25_H_20_ClNO_2_ [M^+^]: 401.1185, found 401.1185.

#### 3.7.4. (E)-3-((E)-1-(4-Methoxyphenyl)-3-(4-nitrophenyl)allylidene)-1-methylindolin-2-one (**3dc**)

Reflux for 20 h; 45% yield; red solid; mp = 143.1–144.6 °C; *R*_f_ = 0.54 (silica gel, hexane:EtOAc:CH_2_Cl_2_ = 4:1:2); ^1^H NMR (500 MHz, CDCl_3_): *δ* 9.48 (d, *J* = 16.0 Hz, 1H), 8.19–8.15 (m, 2H), 7.65 (d, *J* = 8.7 Hz, 2H), 7.22–7.18 (m, 2H), 7.16 (td, *J* = 7.7, 1.1 Hz, 1H), 7.12–7.06 (m, 2H), 6.77 (d, *J* = 7.7 Hz, 1H), 6.67 (td, *J* = 7.7, 1.0 Hz, 1H), 6.51 (d, *J* = 16.0 Hz, 1H), 5.95 (d, *J* = 7.4 Hz, 1H), 3.95 (s, 3H), 3.30 (s, 3H) ppm; ^13^C NMR (125 MHz, CDCl_3_): *δ* 168.0, 160.1, 149.6, 147.5, 143.5, 143.3, 137.8, 132.3, 130.1, 129.2, 129.1 128.3, 125.1, 124.2, 123.2, 121.9, 114.9, 107.9, 55.6, 26.0 ppm; HRMS (EI): calcd for C_25_H_20_N_2_O_4_ [M^+^] : 412.1423, found 412.1422.

#### 3.7.5. (E)-3-((E)-1,3-Bis(4-methoxyphenyl)allylidene)-1-methylindolin-2-one (**3dd**)

91% yield; yellow solid; mp = 131.7 °C; *R*_f_ = 0.21 (silica gel, hexane:EtOAc = 5:1); ^1^H NMR (500 MHz, CDCl_3_): *δ* 9.23 (d, *J* = 15.9 Hz, 1H), 7.49 (d, *J* = 8.8 Hz, 2H), 7.19 (dt, *J* = 13.9, 2.3 Hz, 2H), 7.09 (td, *J* = 7.7, 0.8 Hz, 1H), 7.06 (dt, *J* = 14.0, 2.4 Hz, 2H), 6.85 (d, *J* = 8.8 Hz, 2H), 6.74 (d, *J* = 7.7 Hz, 1H), 6.63 (td, *J* = 7.7, 0.8 Hz, 1H), 6.48 (d, *J* = 15.9 Hz, 1H), 5.89 (d, *J* = 7.7 Hz, 1H), 3.9 (s, 3H), 3.82 (s, 3H), 3.30 (s, 3H) ppm; ^13^C NMR (125 MHz, CDCl_3_): *δ* 168.2, 160.6, 159.8, 151.8, 142.6, 141.3, 130.1, 130.0, 129.6, 127.9, 126.2, 123.8, 123.4, 121.8, 121.5, 114.6, 114.3, 107.5, 55.49, 55.46, 25.8 ppm; HRMS (EI): calcd for C_26_H_23_NO_3_ [M^+^]: 397.1678, found 397.1677.

### 3.8. Dehydration under Basic Conditions *(**3dc**)*

To a stirred solution of the alcohol **8dc** (15.3 mg, 0.0355 mmol) in CH_2_Cl_2_ (1.0 mL) was added NEt_3_ (40 μL, 0.29 mmol, 8 equiv.), MsCl (10 μL, 0.13 mmol, 3.7 equiv.), and DMAP (1.1 mg, 9.0 μmol, 0.25 equiv.) at rt. After stirring for 1 h, the reaction mixture was diluted with sat. aq. NH_4_Cl (5 mL) and EtOAc (30 mL). The organic layer was separated, dried over Na_2_SO_4_, filtered, and concentrated under reduced pressure. The crude residue was purified by column chromatography (silica gel, Hexane:EtOAc:CH_2_Cl_2_ = 4:1:1) to afford aldol adduct **3dc** (11.6 mg, 0.0281 mmol, 79% yield).

### 3.9. (E)-3-((E)-1-(Biphenyl-4-yl)-3-phenylallylidene)-1-methylindolin-2-one *(**9**)*

To a solution of **3ea** (16.4 mg, 0.0354 mmol) in dioxane (1.0 mL) were added phenylboronic acid (5.2 mg, 0.043 mmol, 1.2 equiv.), K_2_CO_3_ (14.7 mg, 0.106 mmol, 3 equiv.) and Pd(PPh_3_)_4_ (2.0 mg, 1.7 μmol, 5 mol%). The reaction mixture was stirred at 90 °C for 8 h, then cooled to rt and diluted with EtOAc (50 mL) and water (20 mL). The organic layer was separated, dried over Na_2_SO_4_, filtered, and concentrated under reduced pressure. The crude residue was purified by column chromatography (silica gel, hexane:EtOAc = 8:1) to afford **9** (12.2 mg, 81% yield) as orange gum. *R*_f_ = 0.43 (silica gel, hexane:EtOAc = 5:1); ^1^H NMR (500 MHz, CDCl_3_): *δ* 9.41 (d, *J* = 16.0 Hz, 1H), 7.85–7.79 (m, 2H), 7.77 (dd, *J* = 8.2, 1.1 Hz, 2H), 7.56 (d, *J* = 7.3 Hz, 2H), 7.53 (t, *J* = 7.7 Hz, 2H), 7.45–7.40 (m, 1H), 7.39–7.35 (m, 2H), 7.33 (t, *J* = 7.3 Hz, 2H), 7.31–7.27 (m, 1H), 7.13 (td, *J* = 7.7, 1.0 Hz, 1H), 6.77 (d, *J* = 7.7 Hz, 1H), 6.63 (td, *J* = 7.7, 0.9 Hz, 1H), 6.56 (d, *J* = 16.0 Hz, 1H), 5.92 (d, *J* = 7.7 Hz, 1H), 3.32 (s, 3H) ppm; ^13^C NMR (125 MHz, CDCl_3_) *δ* 168.1, 151.0, 142.9, 141.6, 141.2, 140.4, 137.0, 136.7, 129.3, 129.2, 129.1, 128.8, 128.4, 128.0, 127.9, 127.7, 127.2, 123.7, 123.4, 122.7, 121.7, 107.7, 25.9 ppm; HRMS (EI): calcd for C_30_H_23_NO [M^+^]: 413.1780, found 413.1776.

## Figures and Tables

**Table 1:** Substrate scope for preparation of aldehyde **7**.

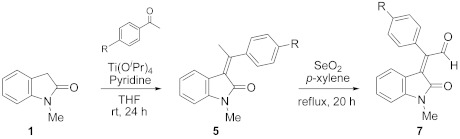						
Entry	5	R	Yield ^1^ (%)	*Z*:*E*^2^	7	Yield ^3^ (%)
1	**5b**	Cl	91	4.6:1	**7b**	85
2	**5c**	NO_2_	95	10:1 ^4^	**7c**	87
3	**5d**	OMe	77	4:1	**7d**	0

^1^ Sum of isolated yields of (*Z*)- and (*E*)-isomers, ^2^ Ratio of isolated yield of (*Z*)- and (*E*)-isomers, ^3^ Isolated yield. ^4^ Ratio in ^1^H NMR of mixture of two isomers, which could not be isolated.

**Table 2:** Substrate scope of the Wittig reaction.

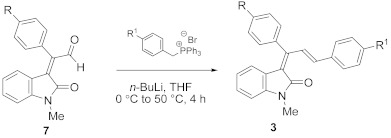					
Entry	7	3	R	R ^1^	Yield ^1^ (%)
1	**7a**	**3aa**	H	H	84
2	**7a**	**3ab**	H	Cl	67
3	**7a**	**3ac**	H	NO_2_	64
4	**7a**	**3ad**	H	OMe	53
5	**7b**	**3ba**	Cl	H	95
6	**7b**	**3bb**	Cl	Cl	79
7	**7b**	**3bc**	Cl	NO_2_	82
8	**7b**	**3bd**	Cl	OMe	68
9	**7c**	**3ca**	NO_2_	H	80
10	**7c**	**3cb**	NO_2_	Cl	79
11	**7c**	**3cc**	NO_2_	NO_2_	68
12	**7c**	**3cd**	NO_2_	OMe	82

^1^ Isolated yield.

**Table 3:** Substrate scope of the aldol/dehydration strategy from **(*Z*)-5d**.

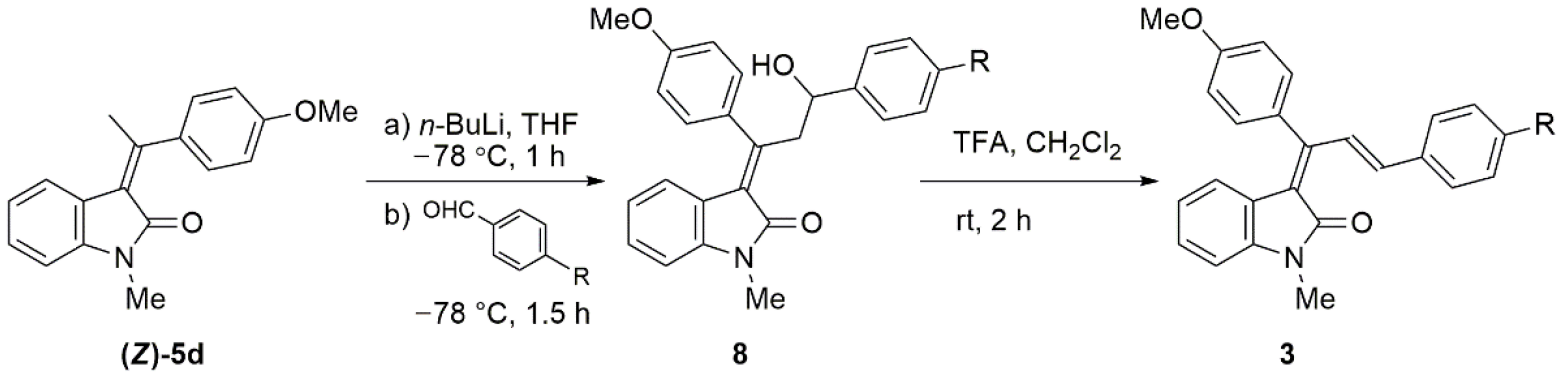					
Entry	R	8	Yield ^1^ (%)	3	Yield ^1^ (%)
1	H	**8da**	93	**3da**	90
2	Cl	**8db**	75	**3db**	93
3	NO_2_	**8dc**	86	**3dc**	45 ^2^
4	NO_2_			**3dc**	79 ^3^
5	OMe	**8dd**	63	**3dd**	91

^1^ Isolated yield, ^2^ Reflux, 20 h, ^3^ TsCl, DMAP, NEt_3_, CH_2_Cl_2_, rt, 3 h.

## Data Availability

The data presented in this study are available in the Supplementary Materials.

## Supplementary Materials

The following supporting information can be downloaded at: https://www.mdpi.com/article/10.3390/molecules27165304/s1, copies of NMR spectra of **4**–**9**, and **3** (**3ac**, **3bc**, **3cc**, **3dc**, **3ea**).

## Sample Availability

Samples of the compounds **3**–**9** are available from the authors.
