# Supplementary material for: Non-Palladium-Catalyzed Approach to the Synthesis of (E)-3-(1,3-Diarylallylidene)Oxindoles
Source: Molecules. 2022 Aug 19;27(16):5304. doi: 10.3390/molecules27165304 (PMC9415770; doi:10.3390/molecules27165304)

<*Supplementary Materials*>

Non-palladium-catalyzed Approach to the Synthesis of (*E*)-3-(1,3-Diarylallylidene)oxindoles

Jahyun Koo, Minsu Kim, Kye Jung Shin and Jae Hong Seo *

Integrated Research Institute of Pharmaceutical Sciences, College of Pharmacy, The Catholic University of Korea, 43 Jibong-ro, Wonmi-gu, Bucheon, Gyeonggi-do 14662, Republic of Korea

E-mail: jaehongseo@catholic.ac.kr

Contents

^{1}H and ^{13}C NMR spectra of **4** 2

^{1}H and ^{13}C NMR spectra of **(*E*/*Z*)-5a-e** 3

^{1}H, ^{13}C, HSQC, HMBC, COSY, ROESY NMR spectra of **6** 12

^{1}H and ^{13}C NMR spectra of **7a-c**, and **7e** 15

^{1}H and ^{13}C NMR spectra of **8** 19

^{1}H and ^{13}C NMR spectra of **3ac**, **3bc**, **3cc**, **3dc**, and **3ea** 24

^{1}H and ^{13}C NMR spectra of **9** 29

Some spectra have peaks from other isomer and impurities (remained solvents, grease, TMS).

Example: CH_2Cl_2 (s, 5.30 ppm), grease (m, 0.84-0.87 ppm and bs, 1.25 ppm), TMS (s, 0.00 ppm)

1-Methyl-3-(3-oxo-1,3-diphenylpropyl)indolin-2-one (**4**)

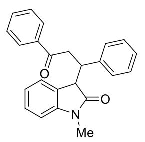

4

^{1}H NMR

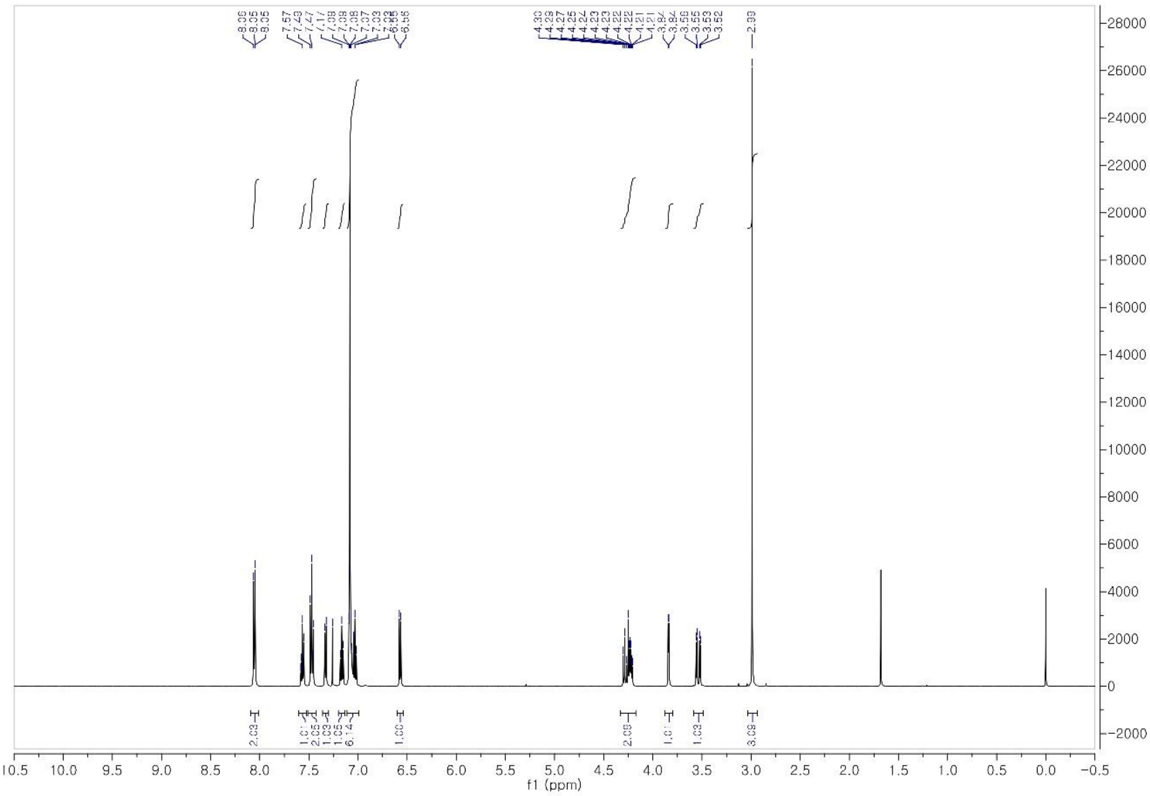

^{13}C NMR

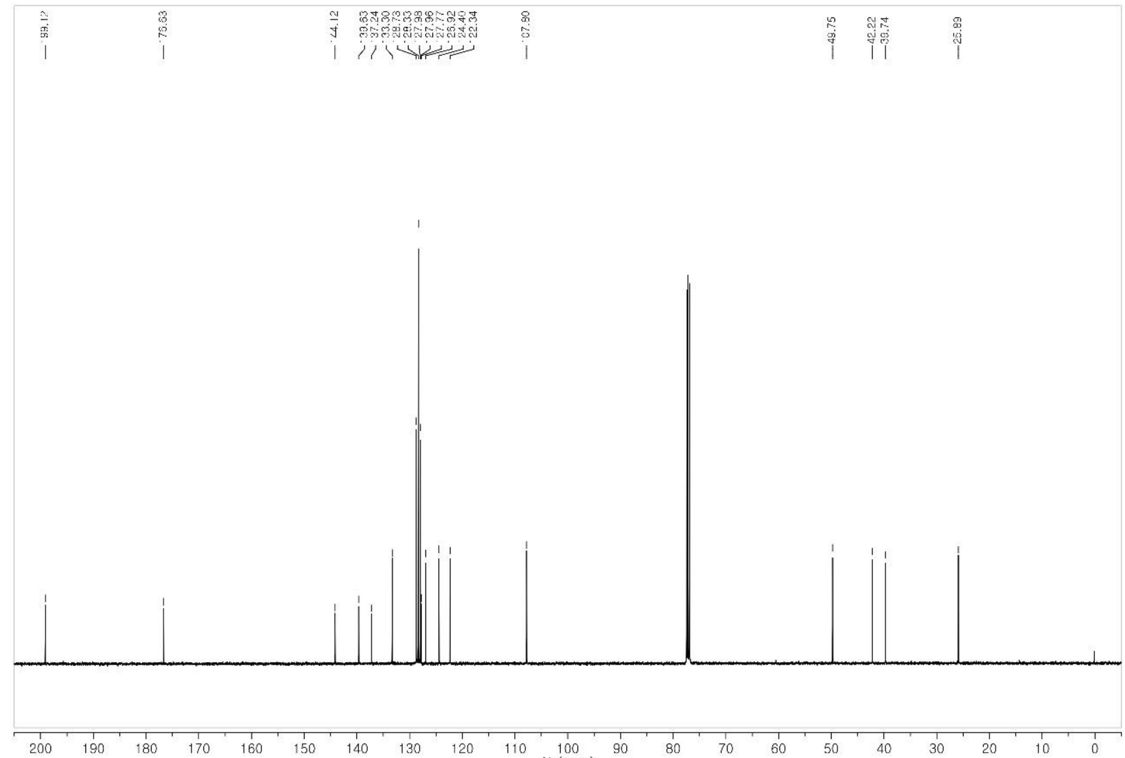

(*Z*)-1-Methyl-3-(1-phenylethylidene)indolin-2-one (**(*Z*)-5a**)

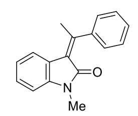

(Z)-5a

^{1}H NMR

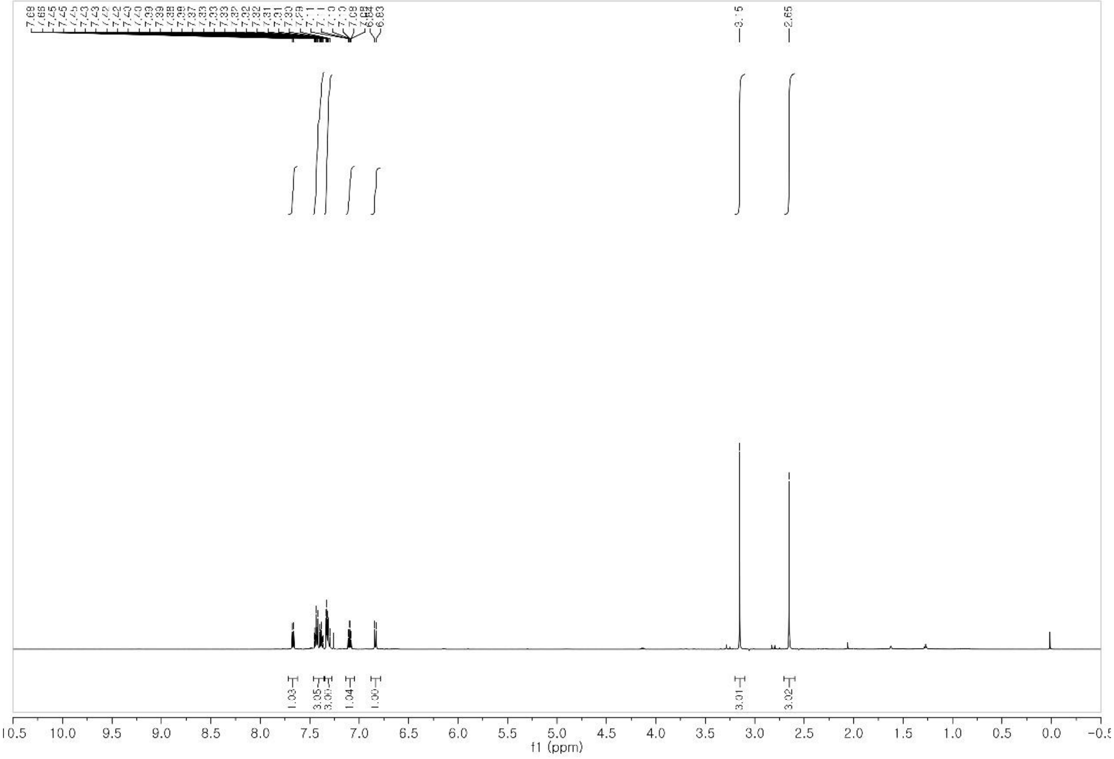

^{13}C NMR

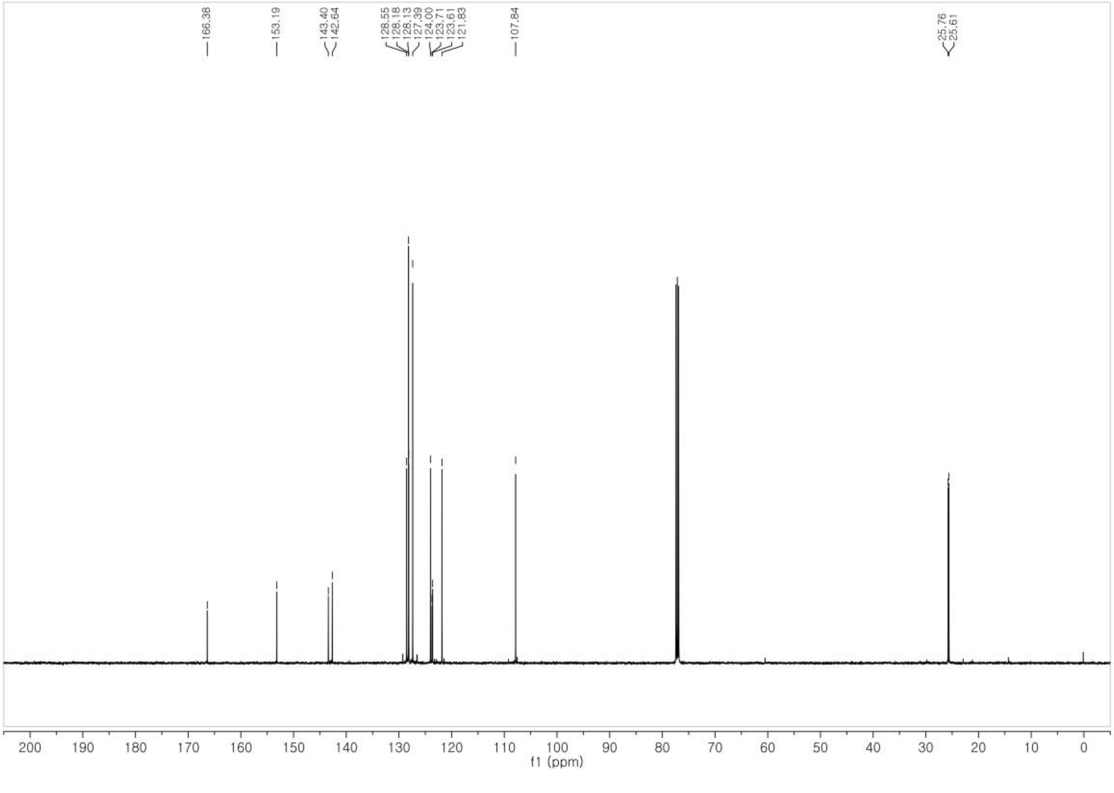

(*E*)-1-Methyl-3-(1-phenylethylidene)indolin-2-one (**(*E*)-5a**)

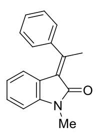

(*E*)-5a

^{1}H NMR

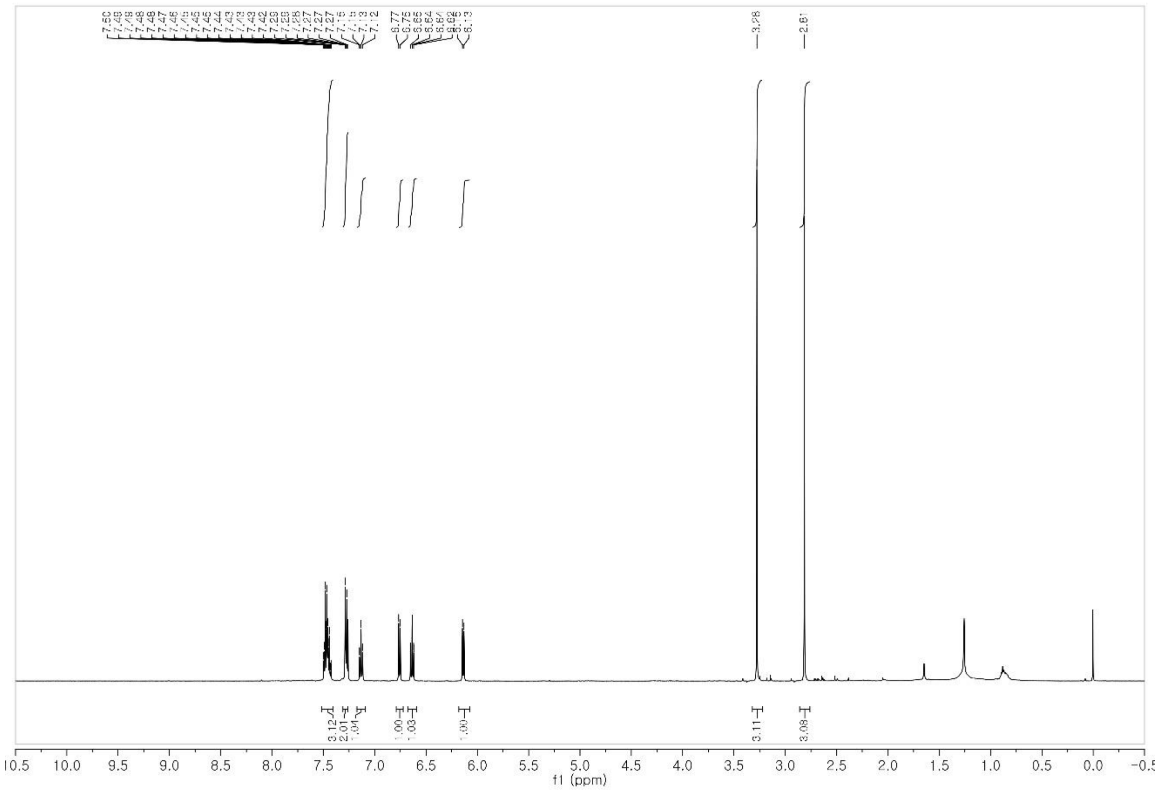

^{13}C NMR

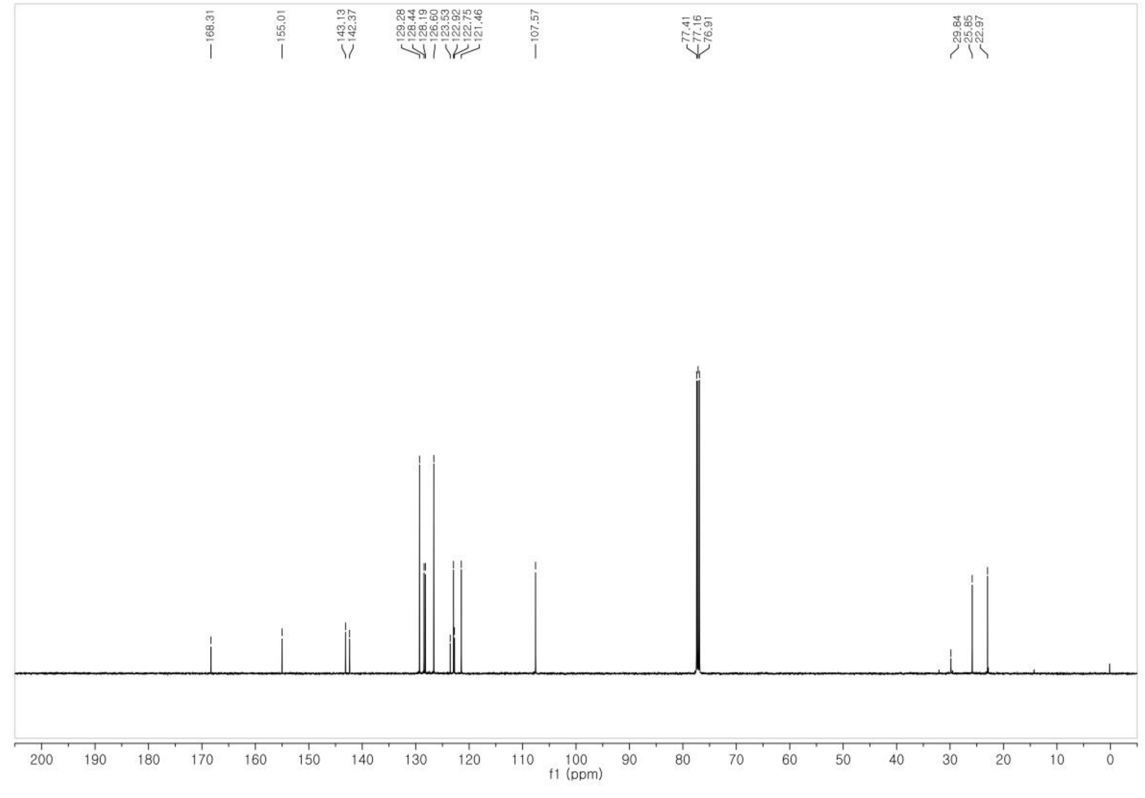

(*Z*)-3-(1-(4-Chlorophenyl)ethylidene)-1-methylindolin-2-one ((*Z*)-**5b**)

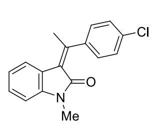

(*Z*)-5b

^{1}H NMR

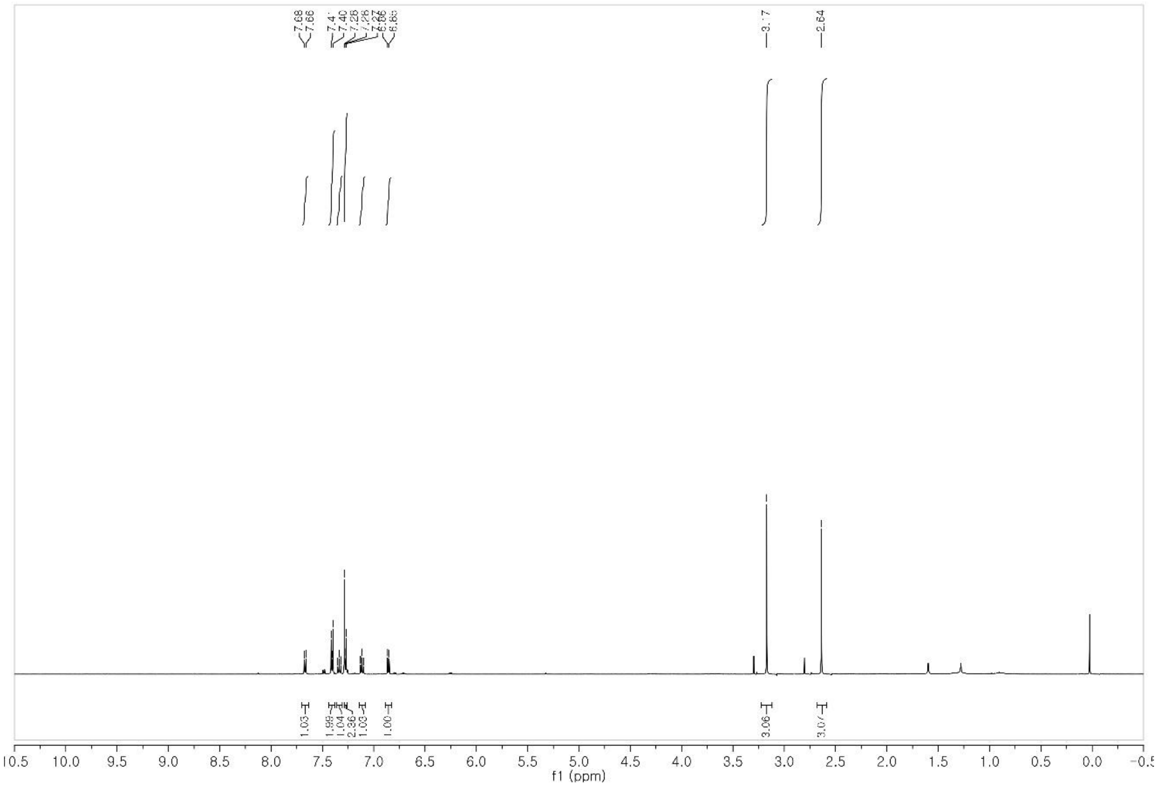

^{13}C NMR

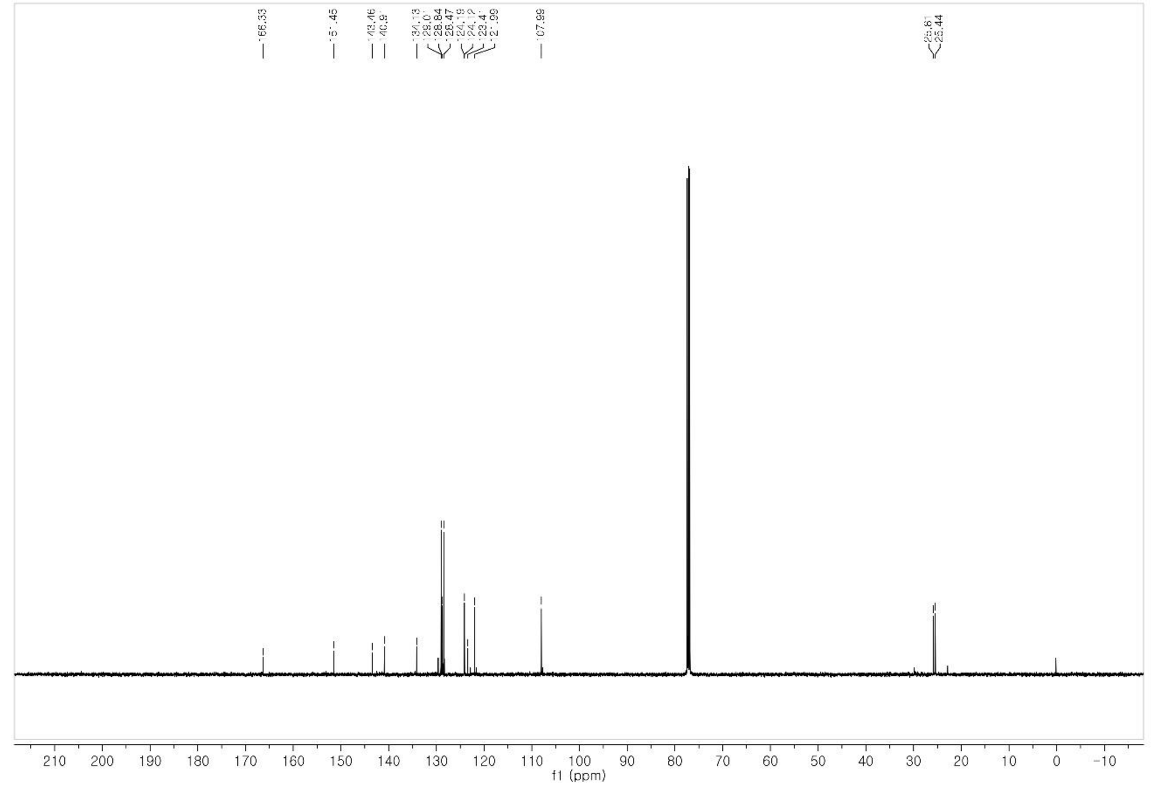

(*E*)-3-(1-(4-Chlorophenyl)ethylidene)-1-methylindolin-2-one (**(*E*)-5b**)

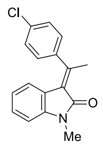

(*E*)-5b

^{1}H NMR

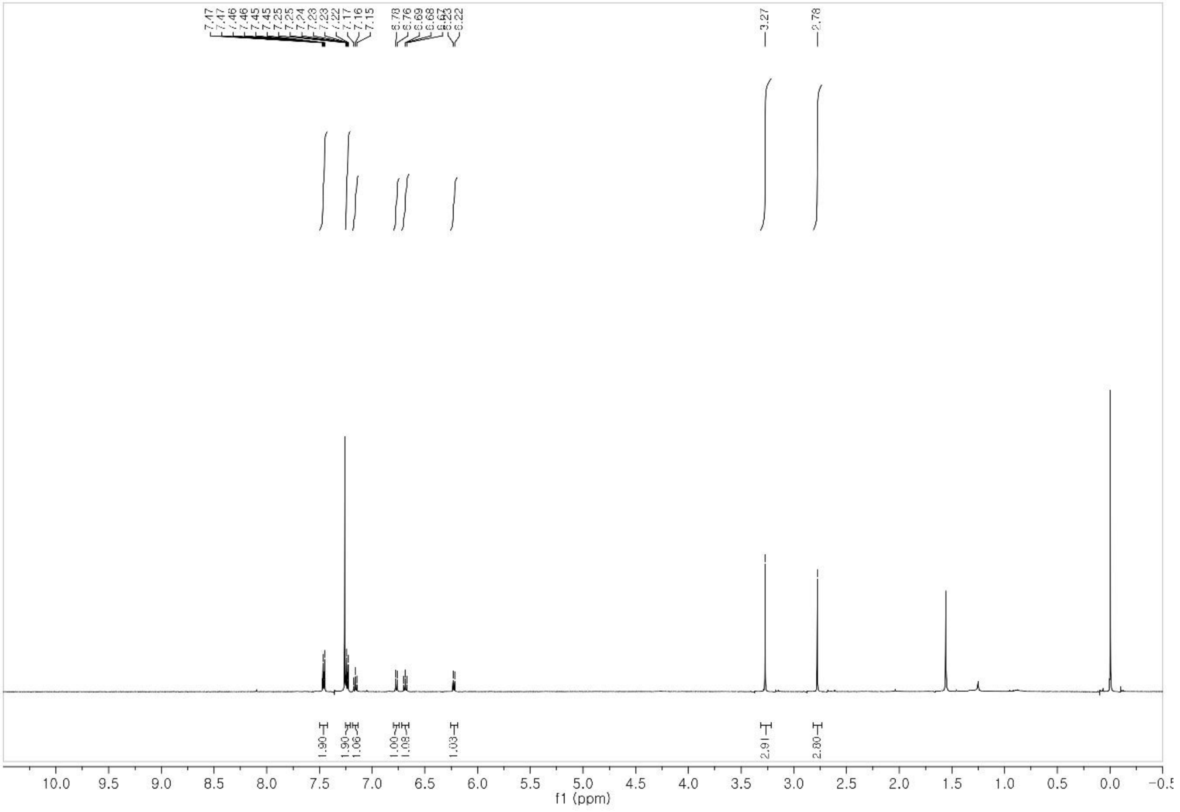

^{13}C NMR

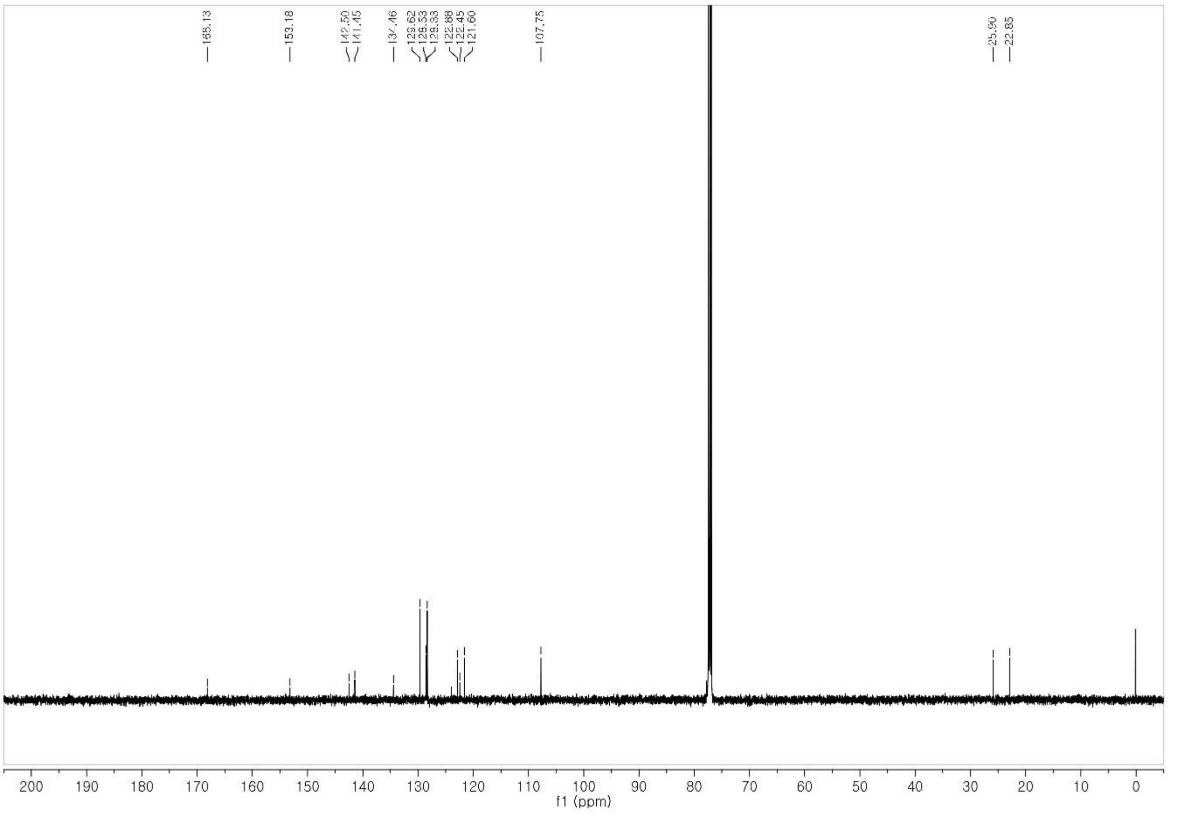

(*Z*)-1-Methyl-3-(1-(4-nitrophenyl)ethylidene)indolin-2-one (***(Z)*-5c**)

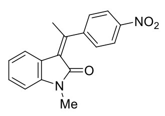

(Z)-5c

^{1}H NMR

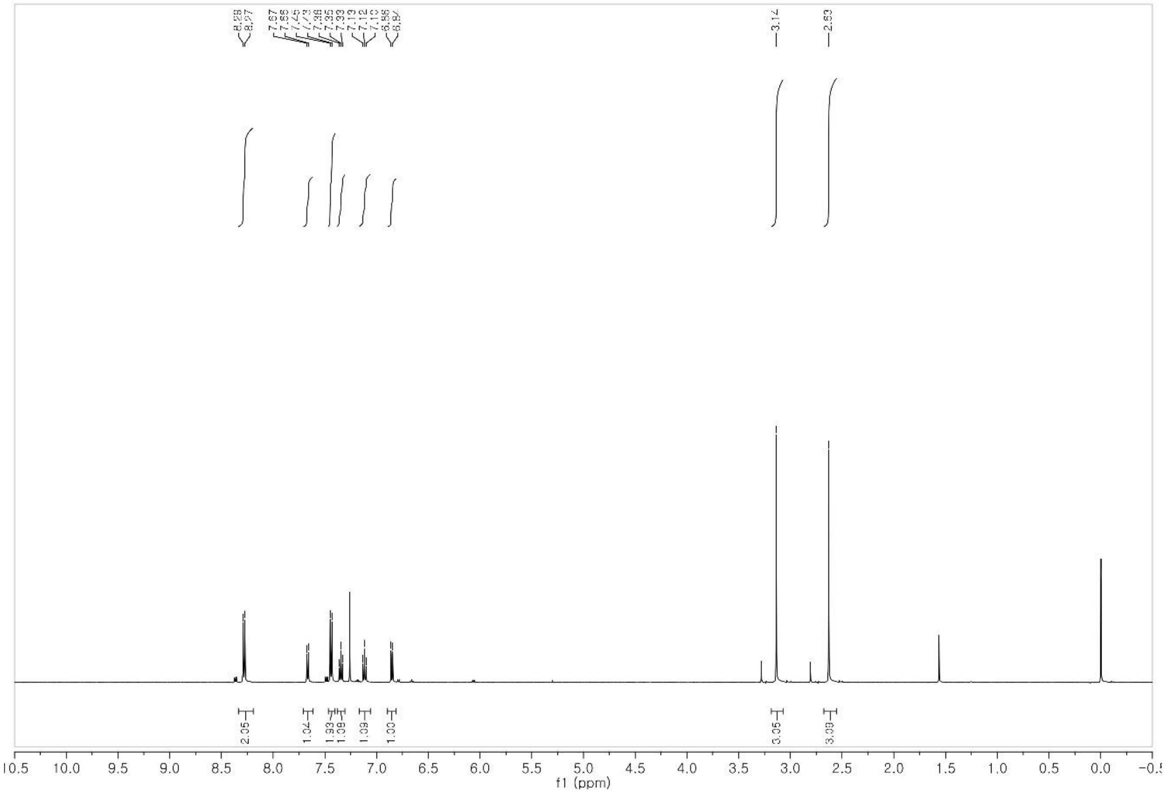

^{13}C NMR

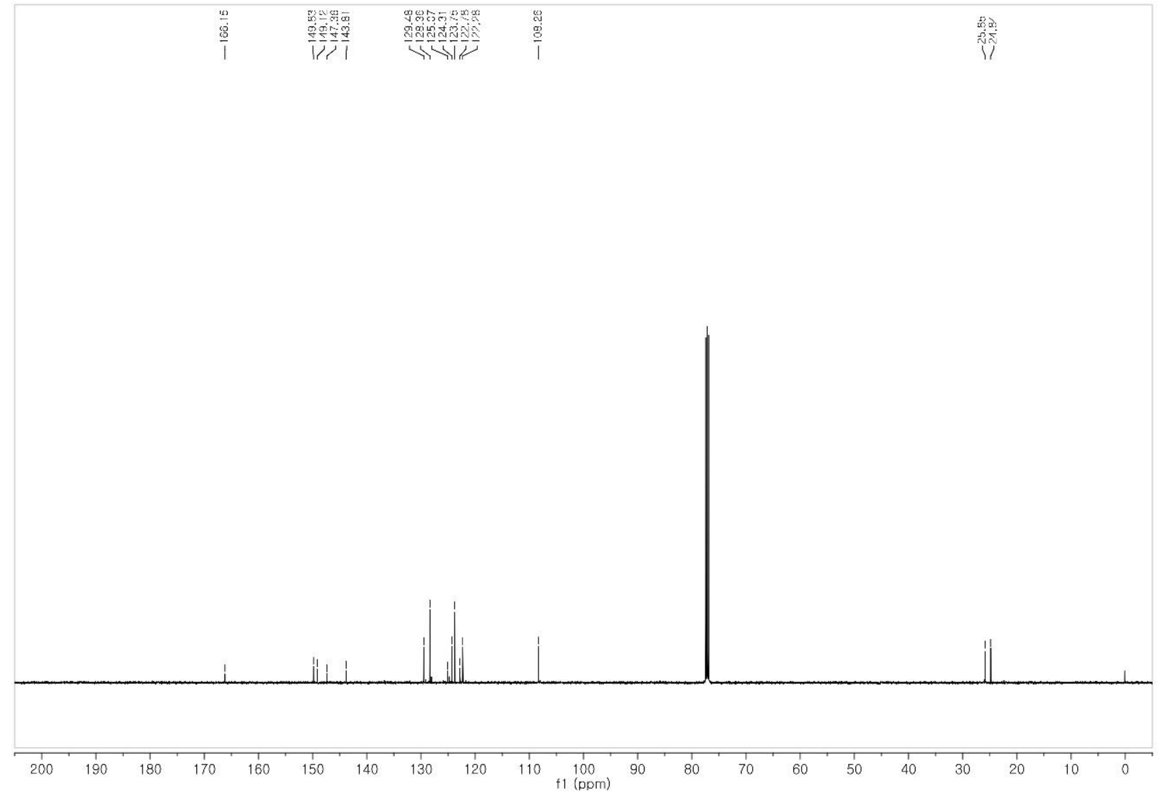

(*Z*)-3-(1-(4-Methoxyphenyl)ethylidene)-1-methylindolin-2-one (***(Z)*-5d**)

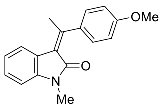

(*Z*)-5d

^{1}H NMR

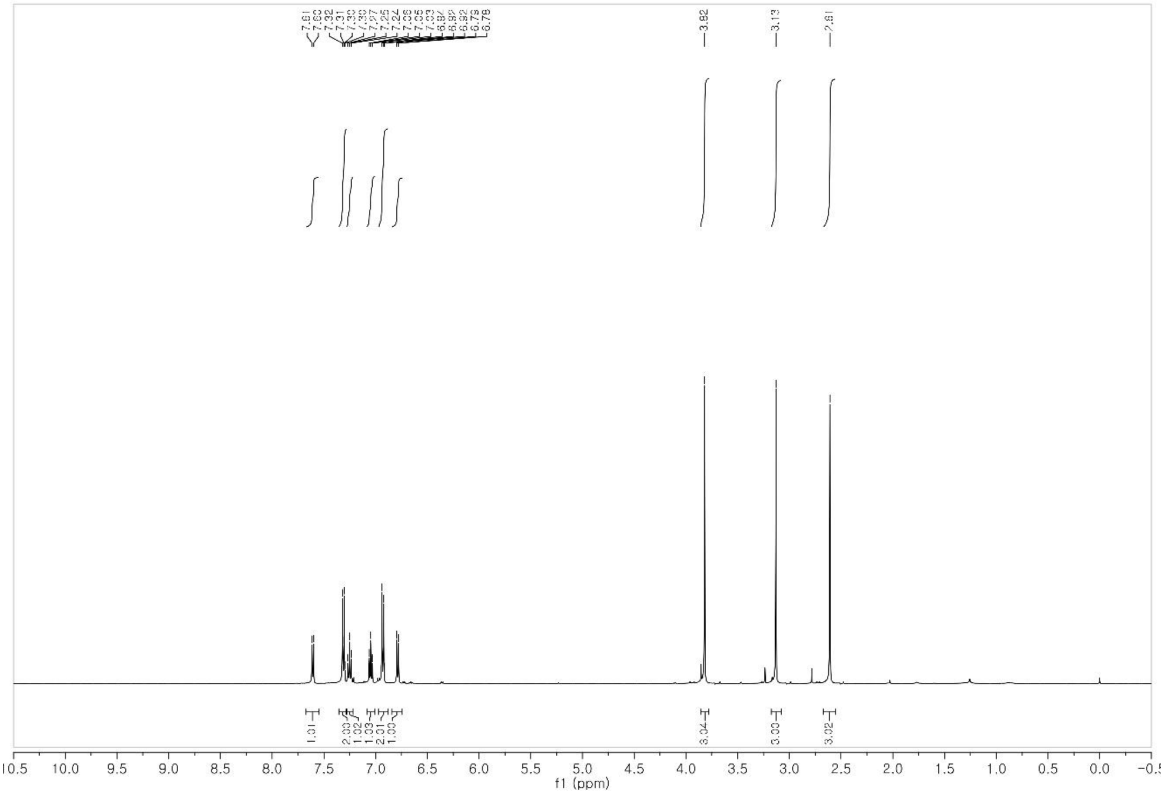

^{13}C NMR

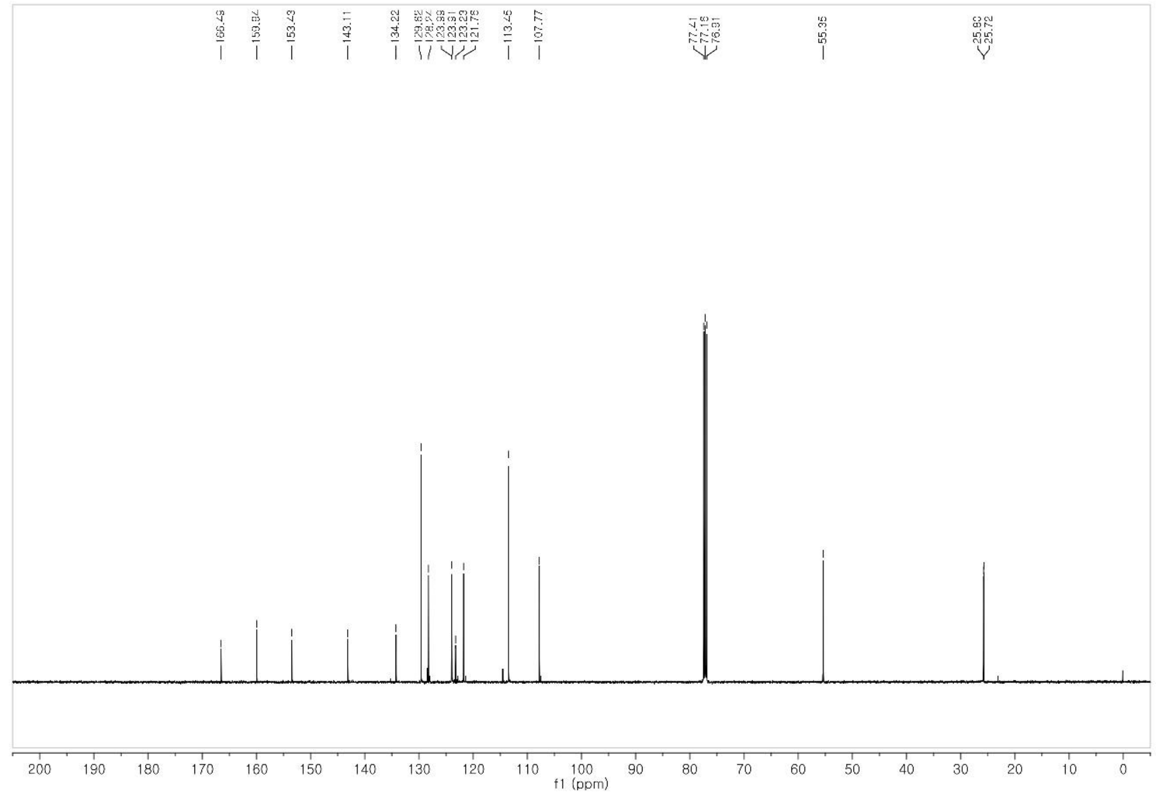

(*E*)-3-(1-(4-Methoxyphenyl)ethylidene)-1-methylindolin-2-one (**(*E*)-5d**)

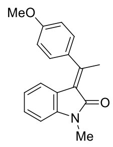

(*E*)-5d

^{1}H NMR

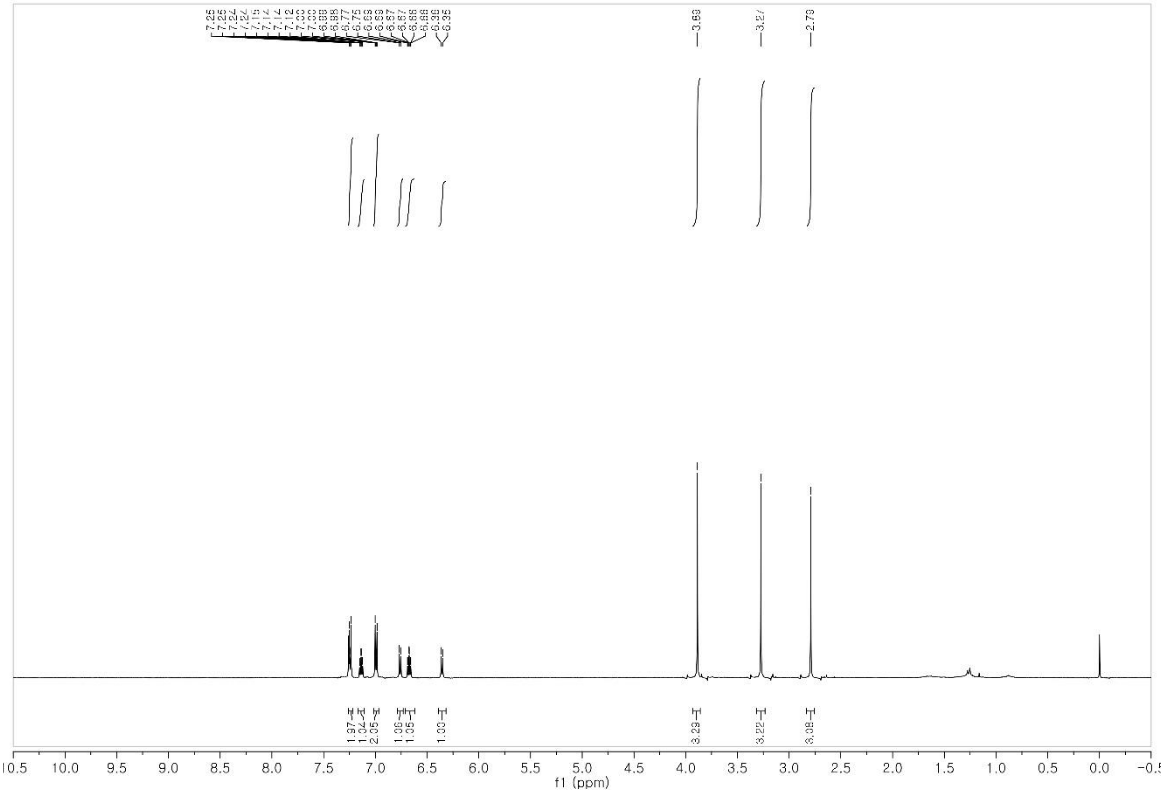

^{13}C NMR

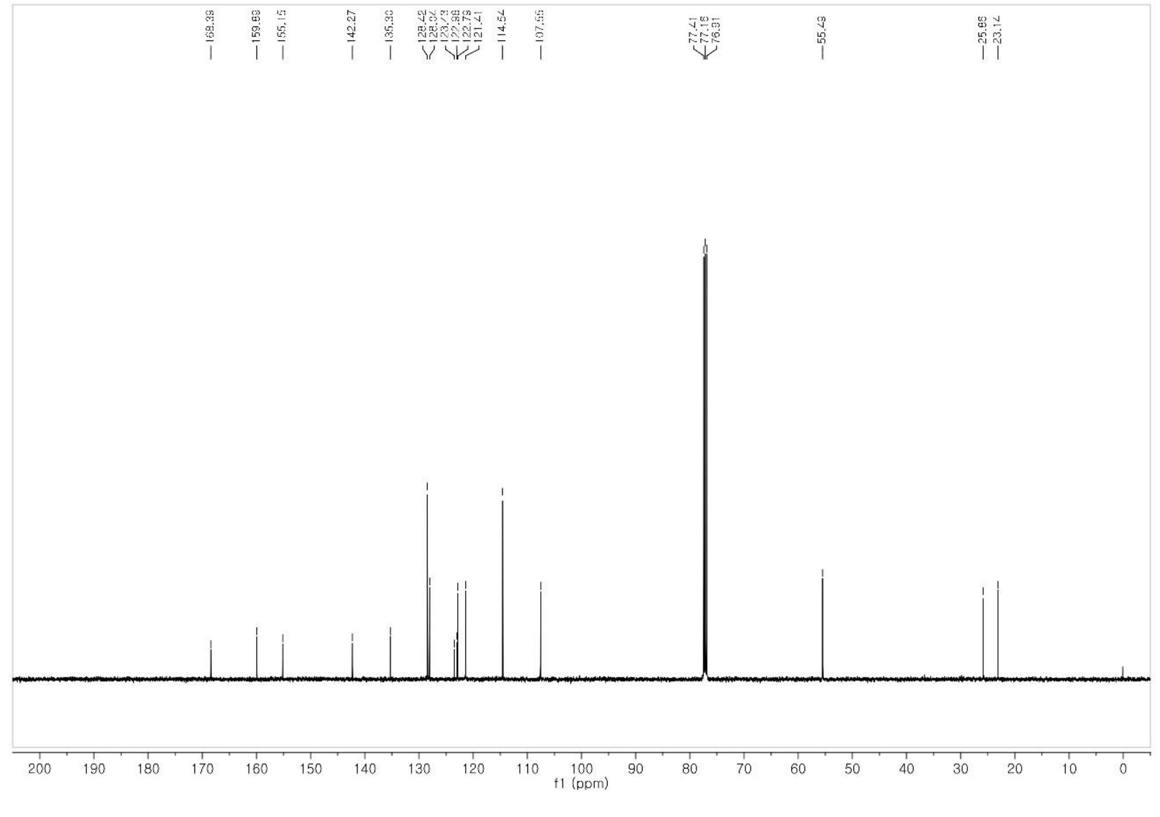

(*Z*)-3-(1-(4-Iodophenyl)ethylidene)-1-methylindolin-2-one (**(*Z*)-5e**)

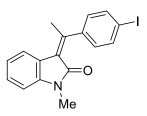

(*Z*)-5e

^{1}H NMR

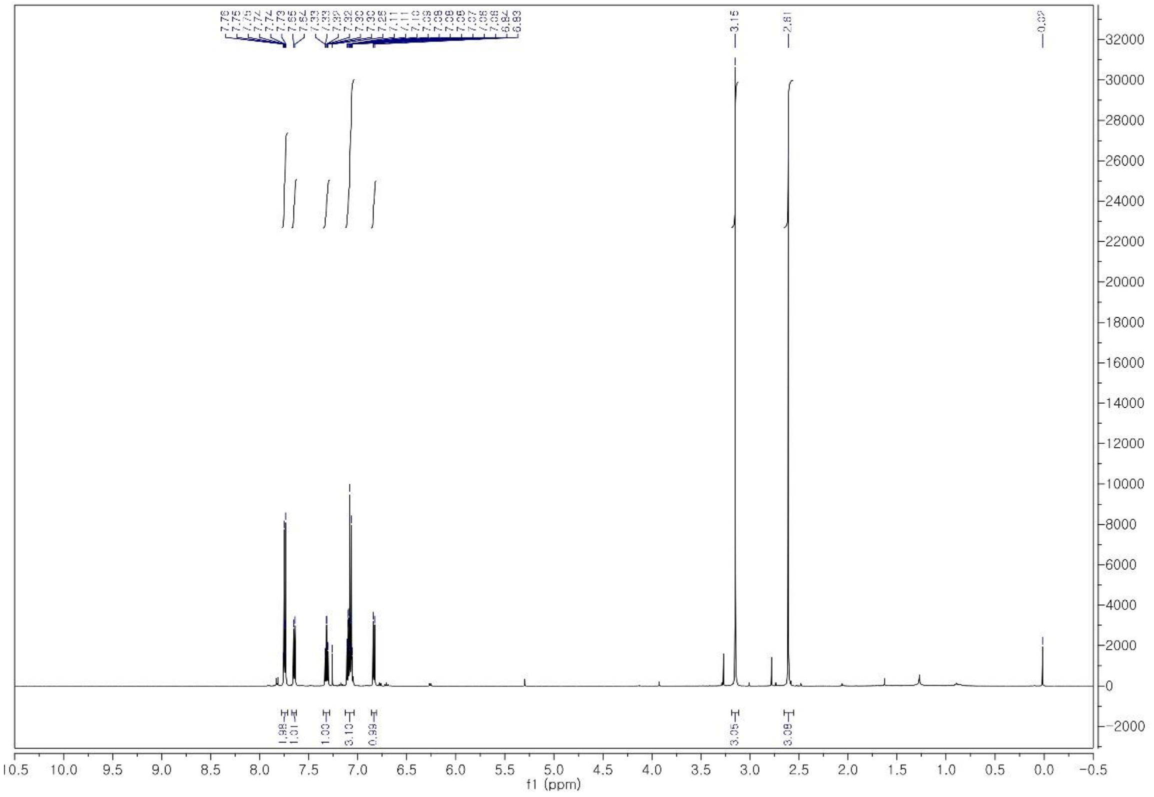

^{13}C NMR

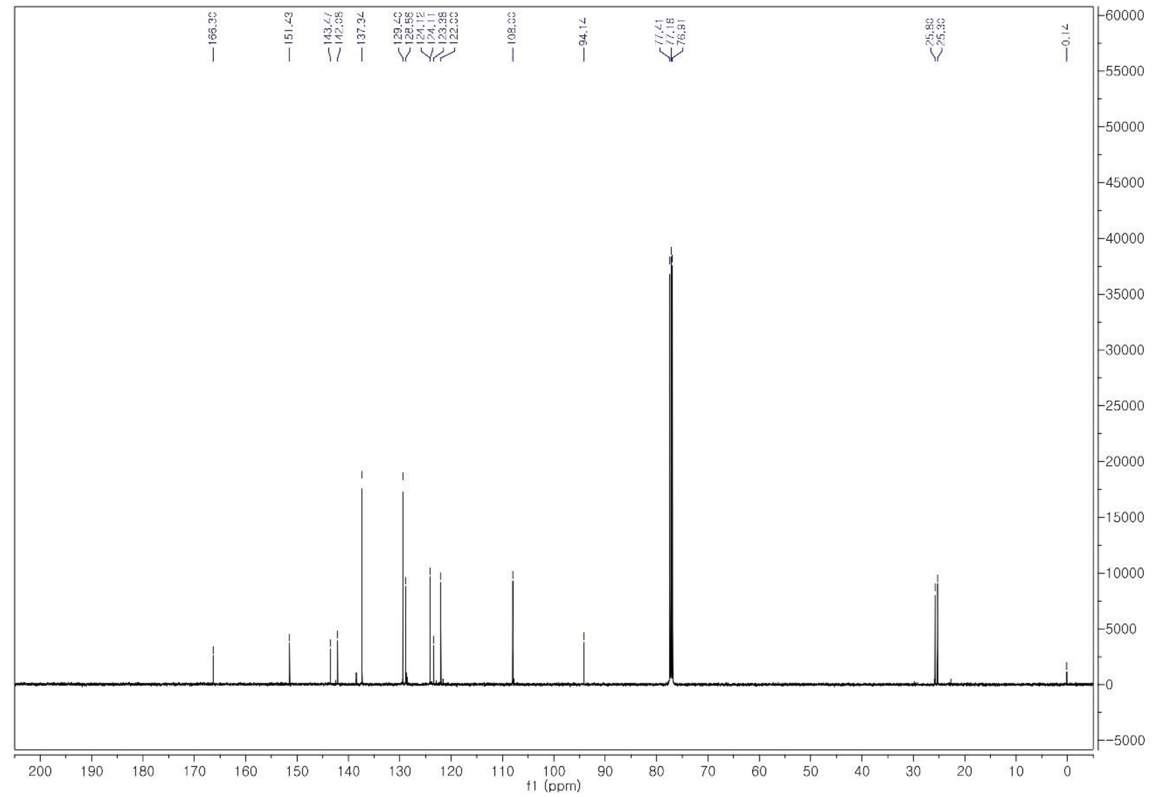

(*E*)-3-(1-(4-Iodophenyl)ethylidene)-1-methylindolin-2-one (**(*E*)-5e**)

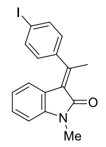

(*E*)-5e

^{1}H NMR

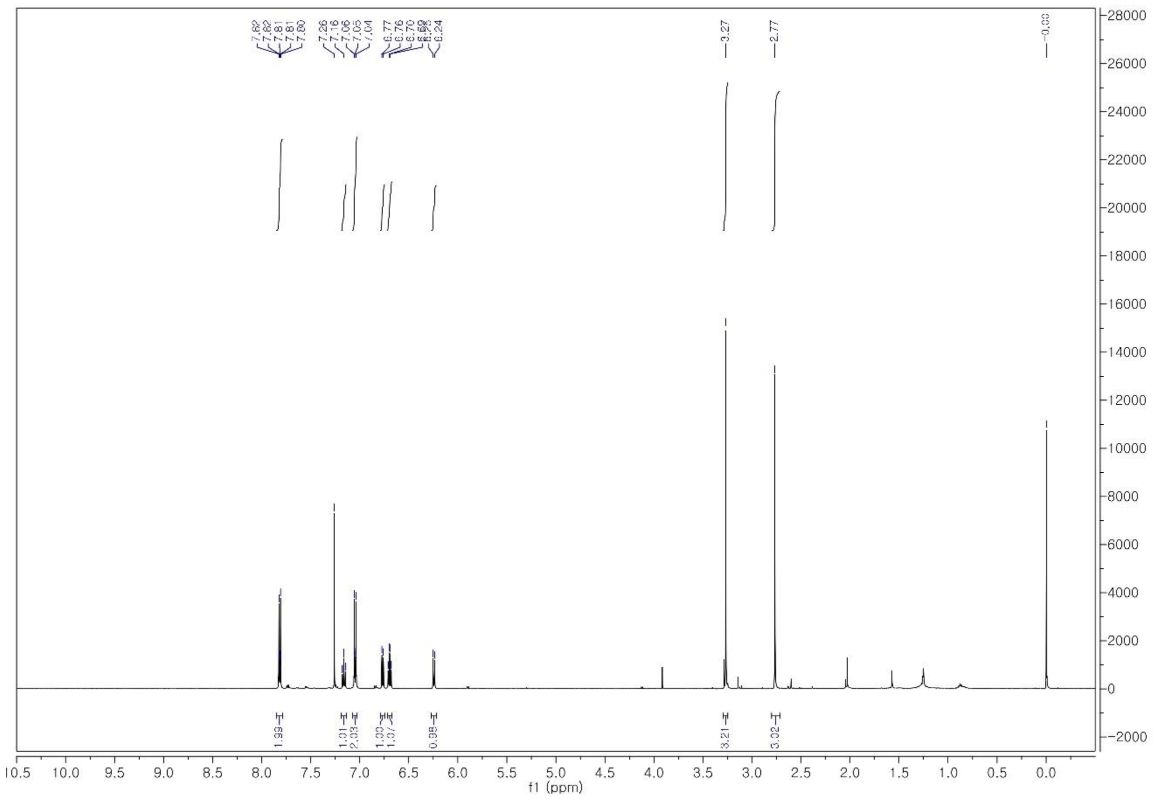

^{13}C NMR

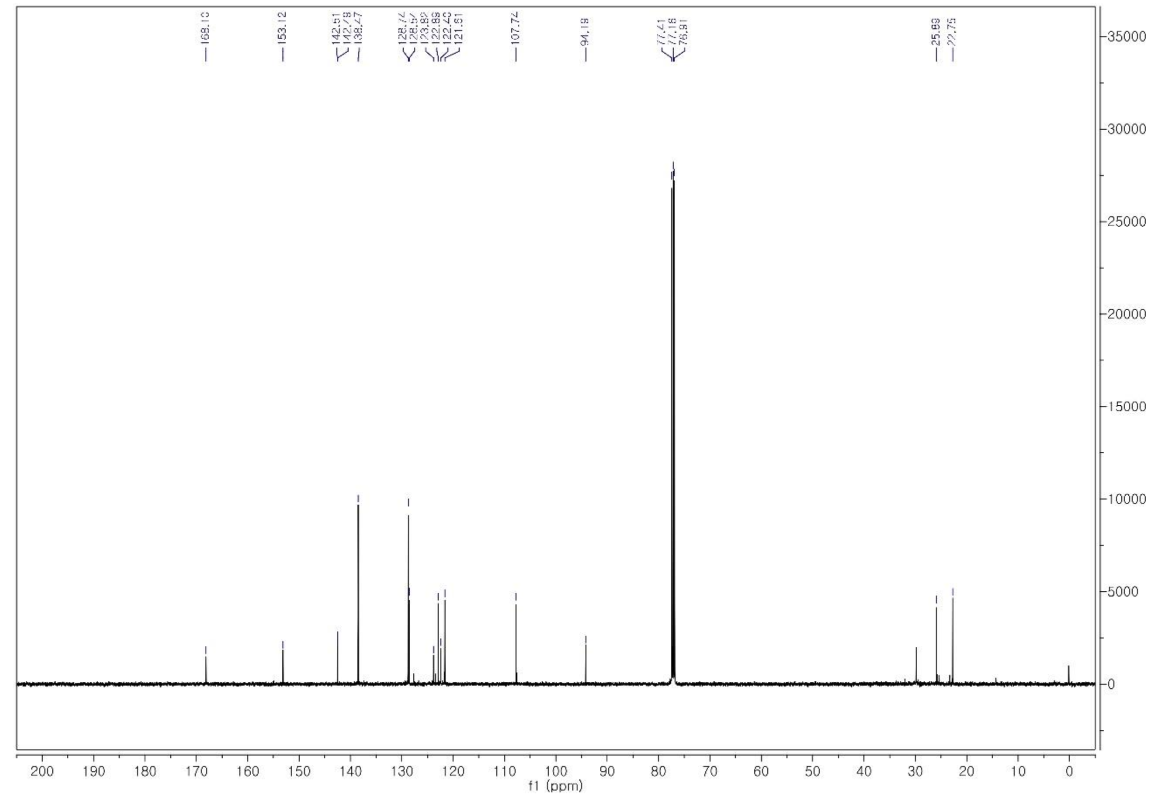

(*Z*)-3-(2-Bromo-1-phenylethylidene)-1-methylindolin-2-one (**6**)

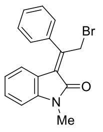

6

^{1}H NMR

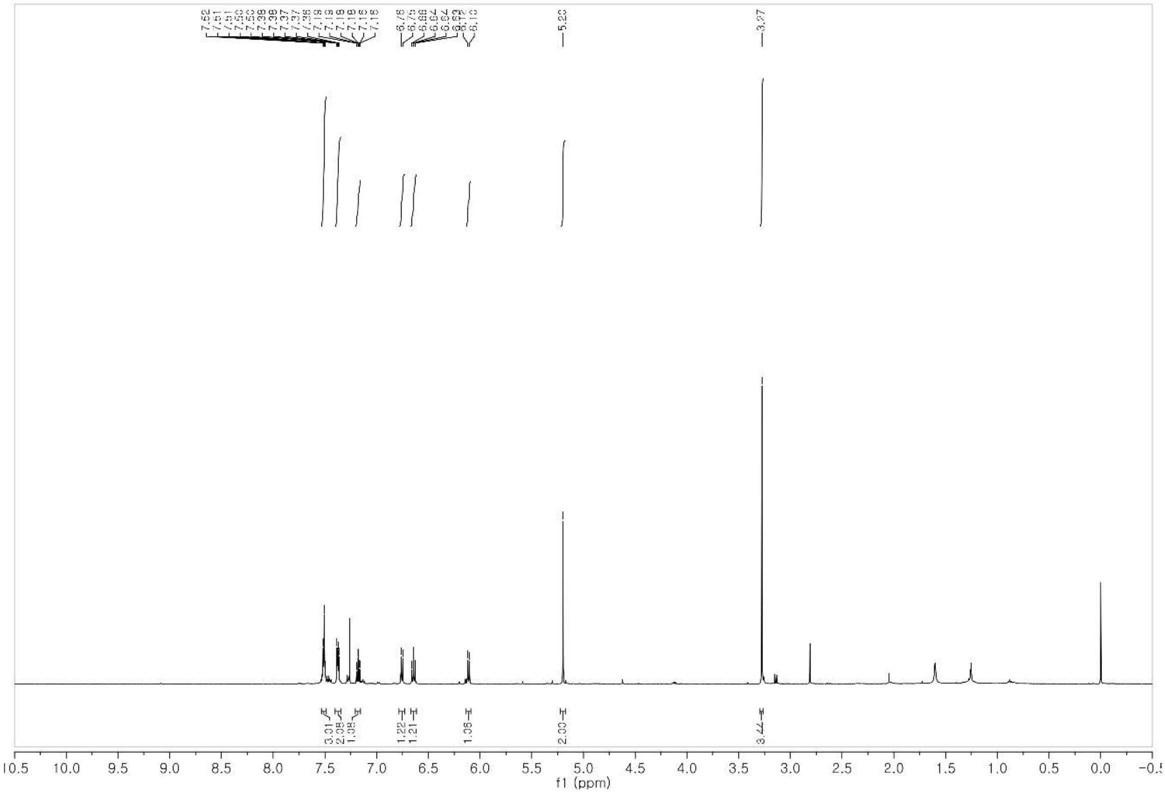

^{13}C NMR

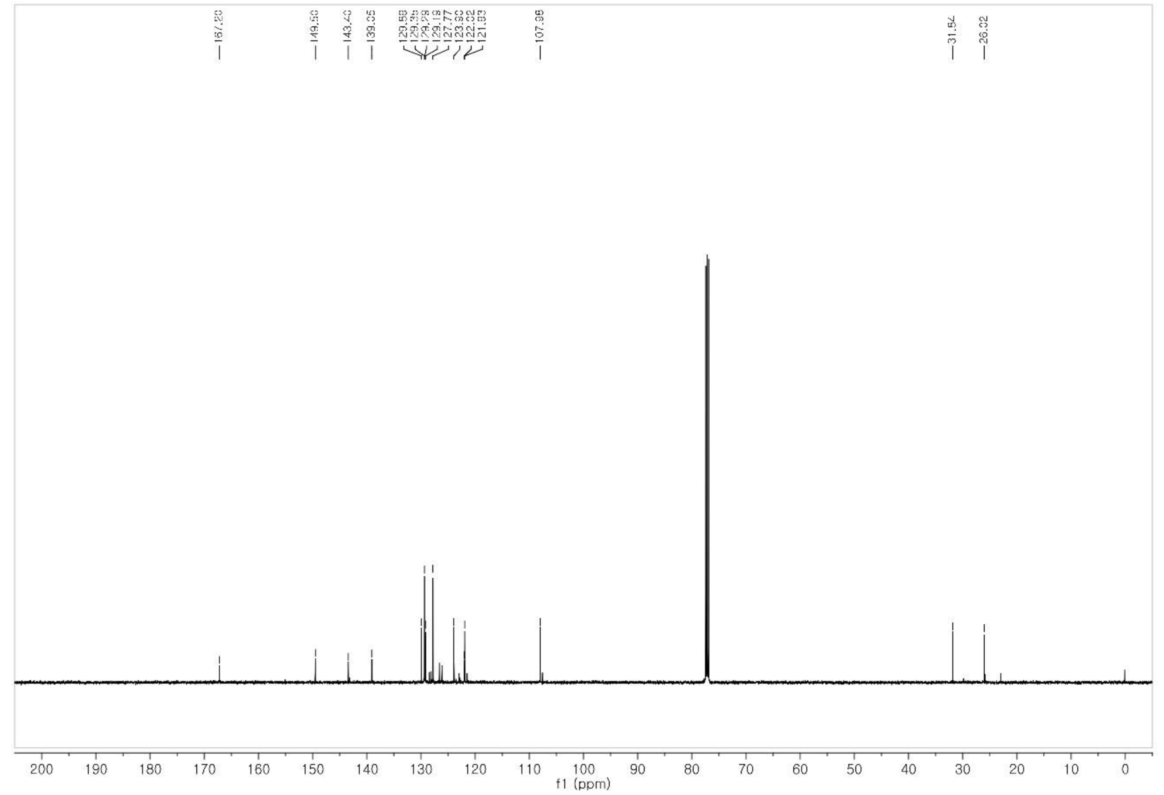

(*Z*)-3-(2-Bromo-1-phenylethylidene)-1-methylindolin-2-one (**6**)

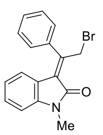

6

HSQC

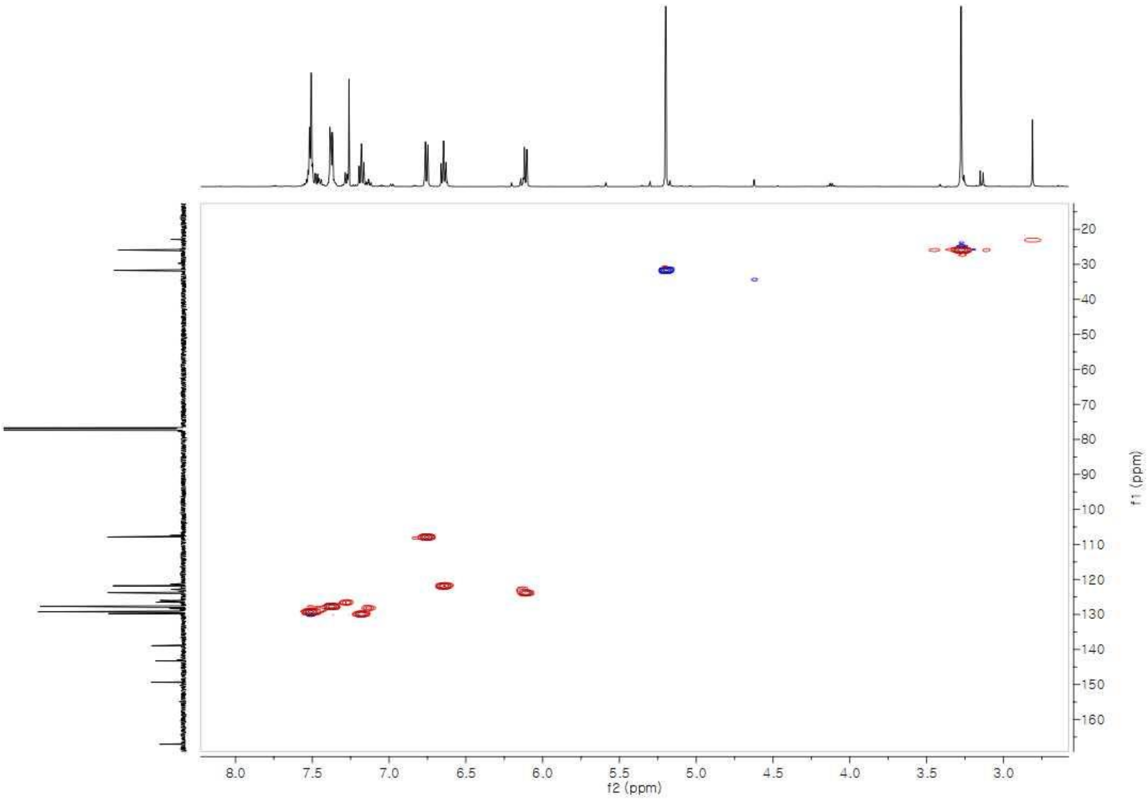

HMBC

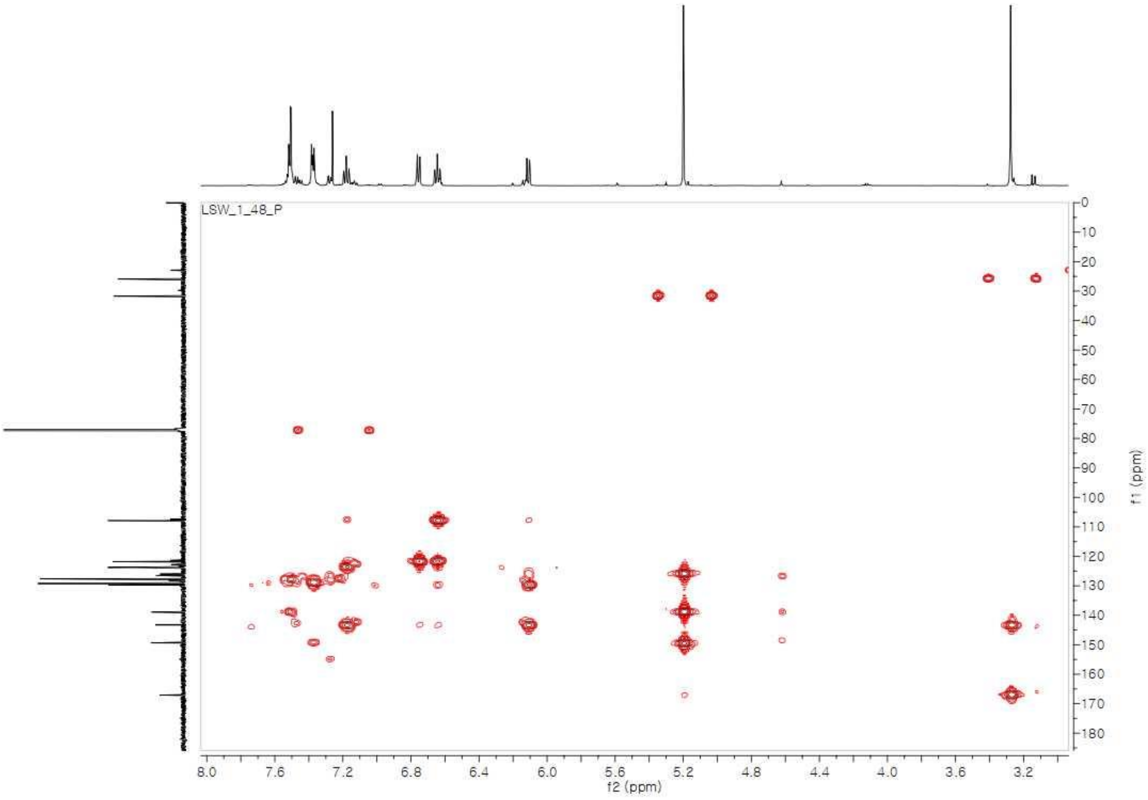

(*Z*)-3-(2-Bromo-1-phenylethylidene)-1-methylindolin-2-one (**6**)

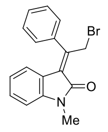

6

COSY

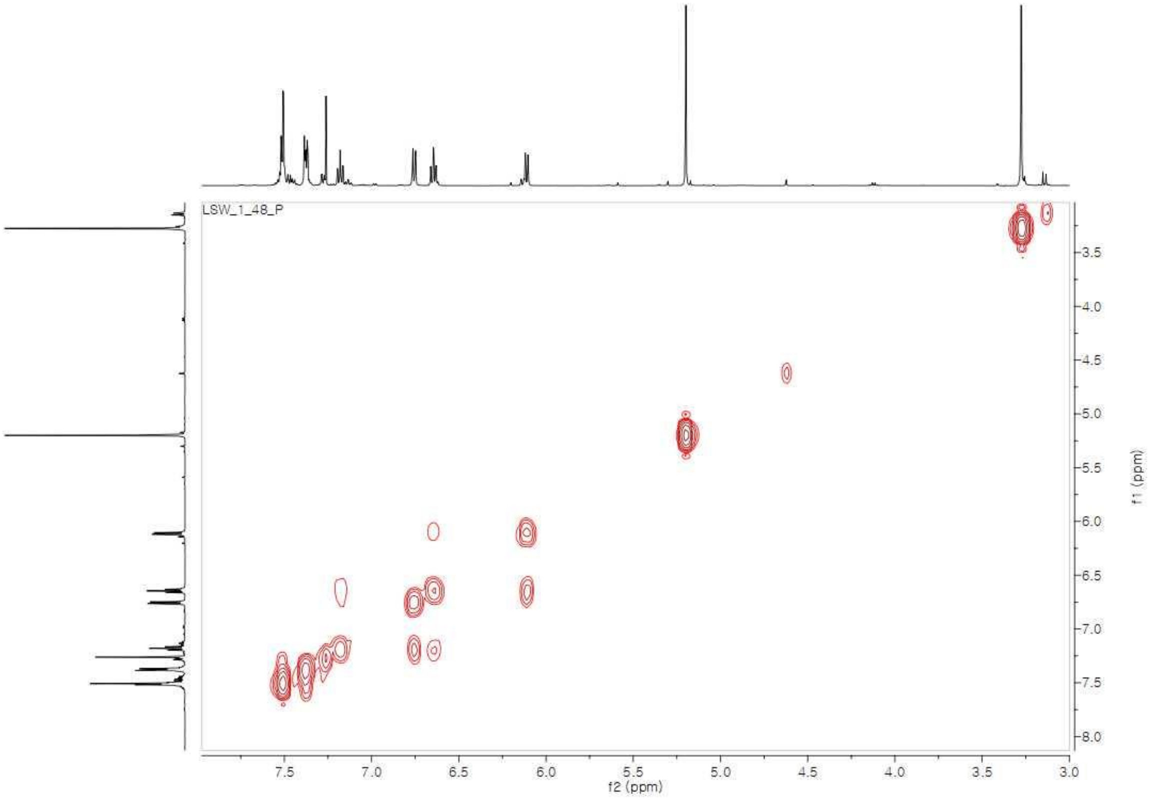

¹ROESY

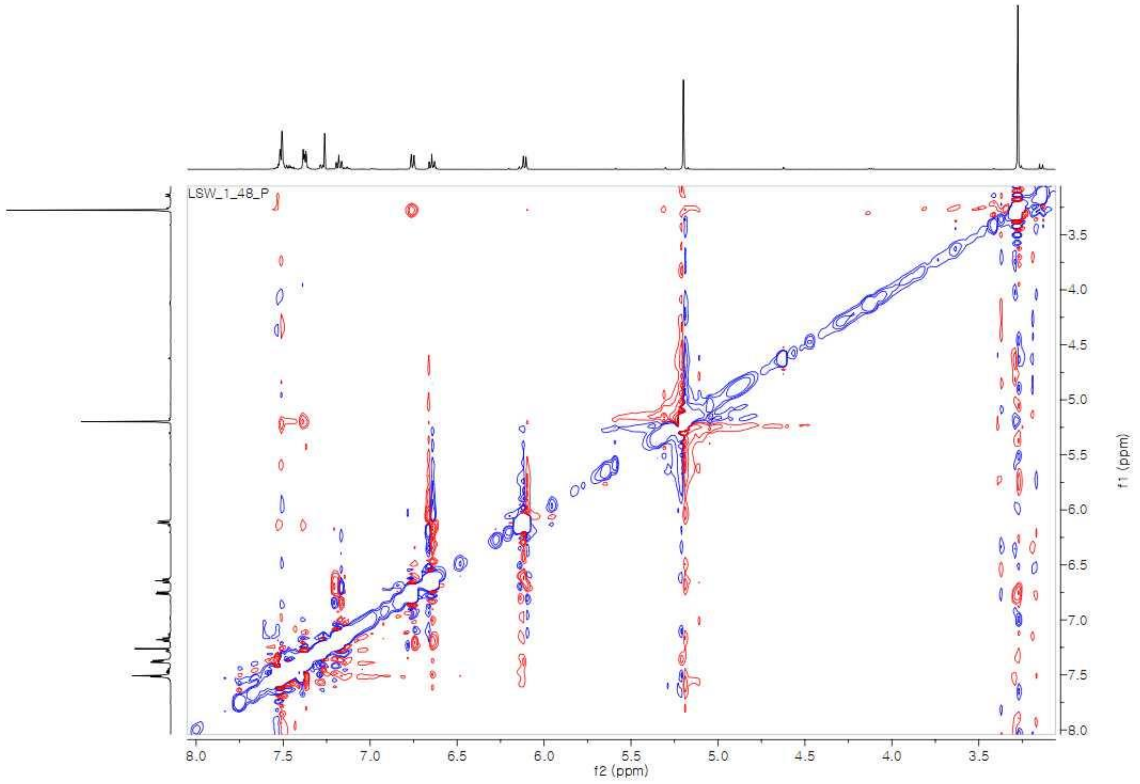

(*Z*)-2-(1-Methyl-2-oxoindolin-3-ylidene)-2-phenylacetaldehyde (**7a**)

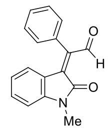

7a

^{1}H NMR

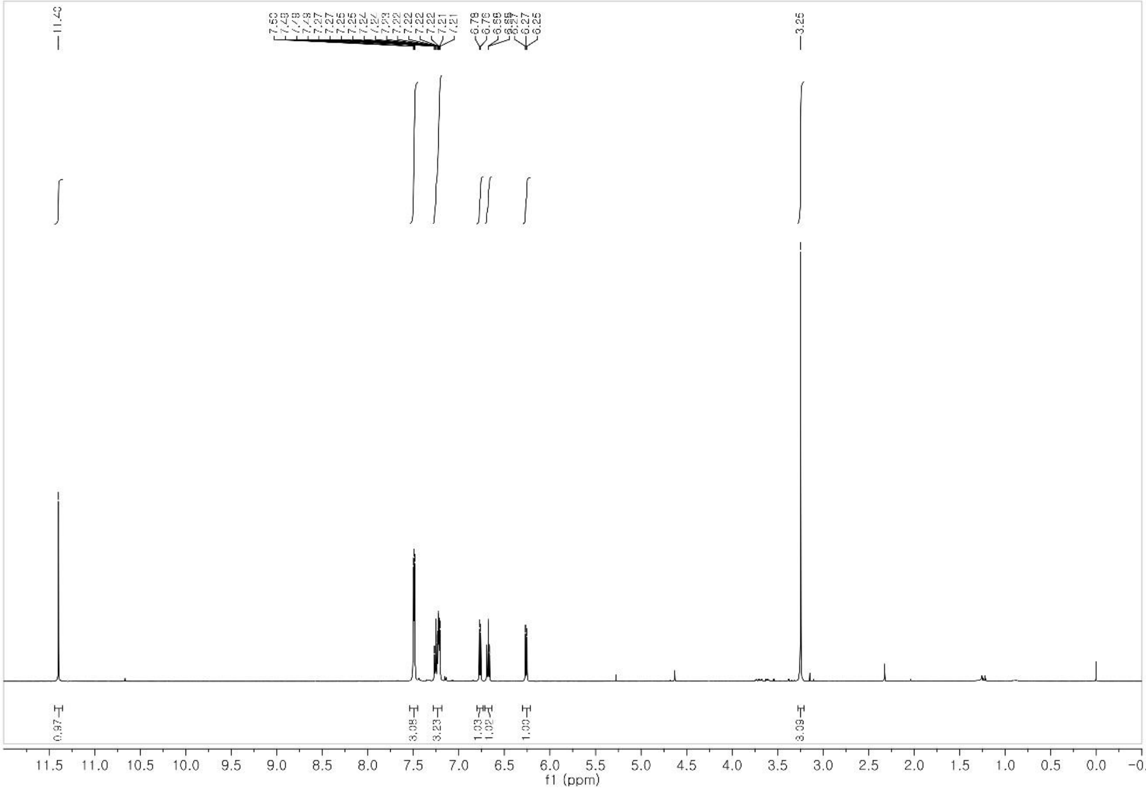

^{13}C NMR

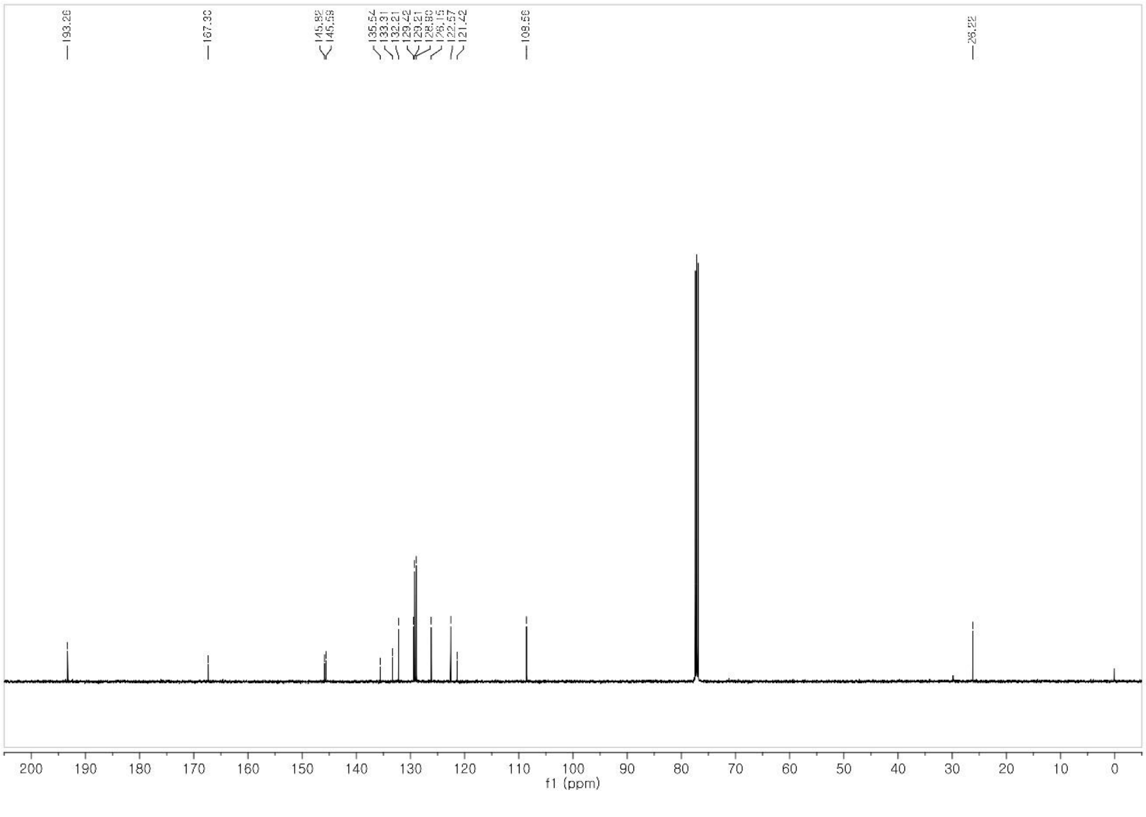

(*Z*)-2-(4-Chlorophenyl)-2-(1-methyl-2-oxoindolin-3-ylidene)acetaldehyde (**7b**)

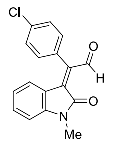

7b

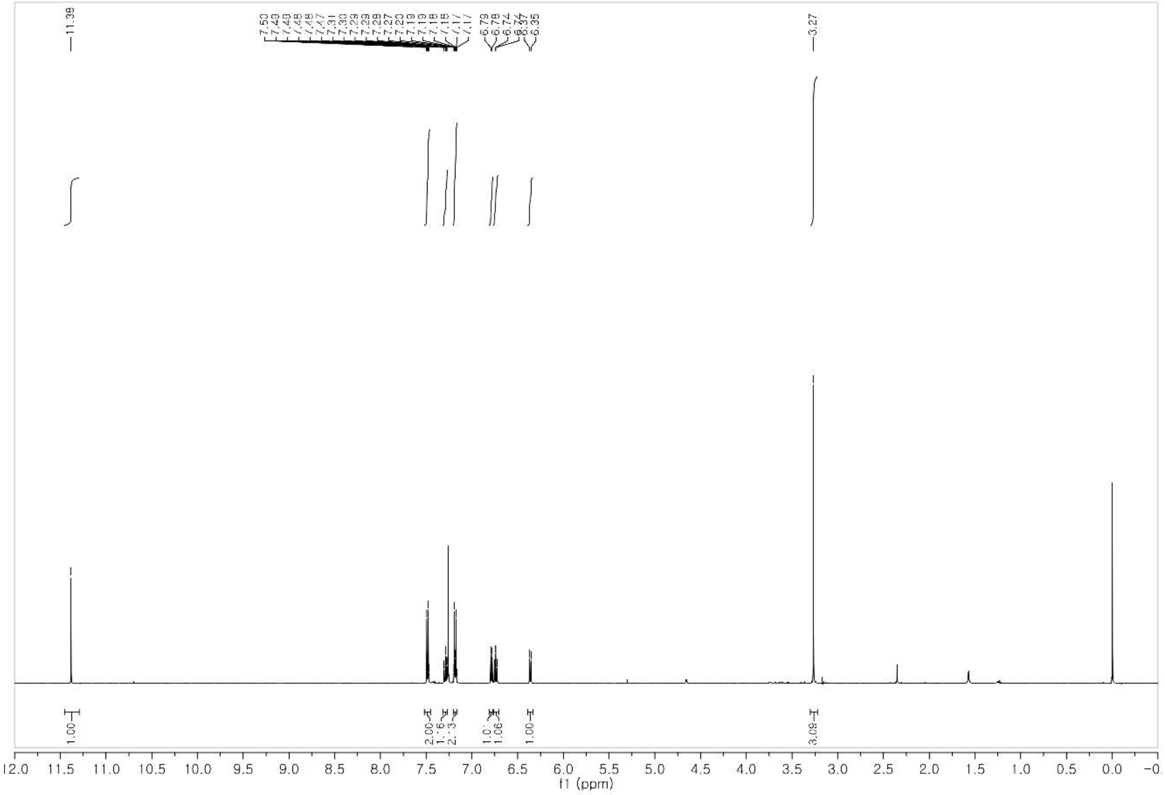

^{1}H NMR

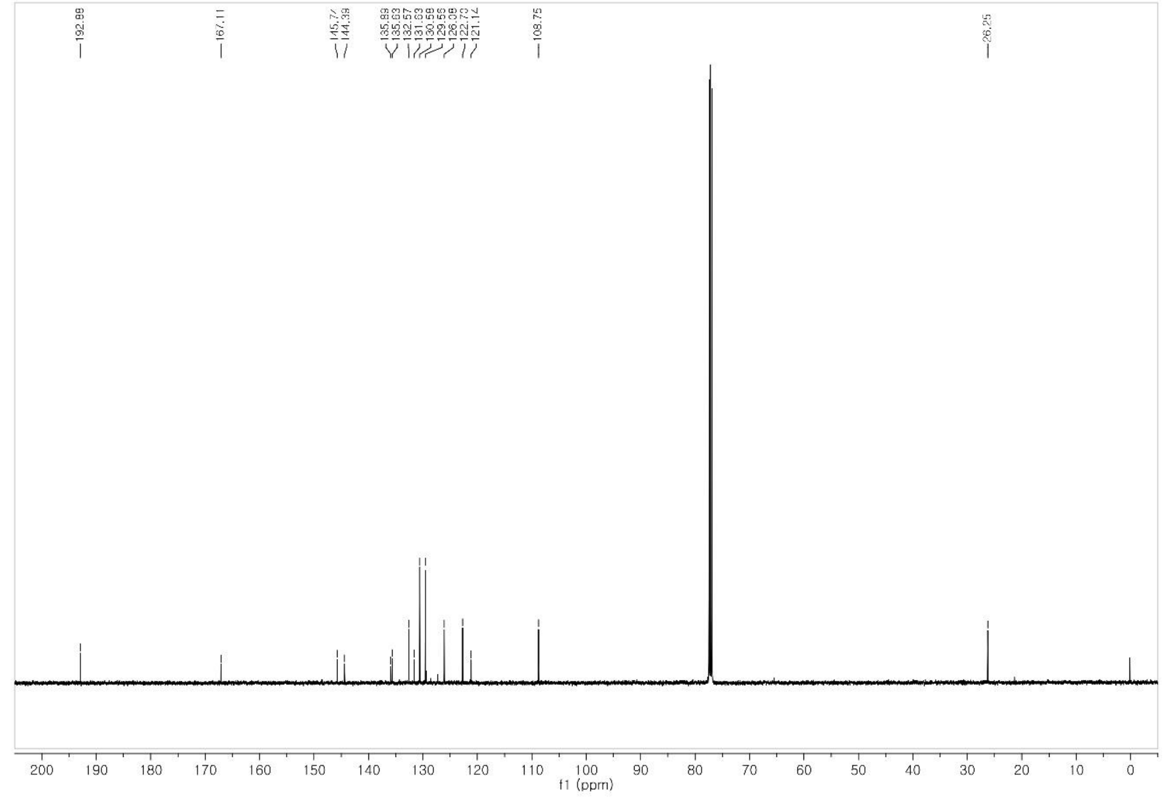

^{13}C NMR

(*Z*)-2-(1-Methyl-2-oxoindolin-3-ylidene)-2-(4-nitrophenyl)acetaldehyde (**7c**)

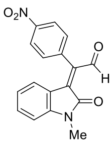

7c

^{1}H NMR

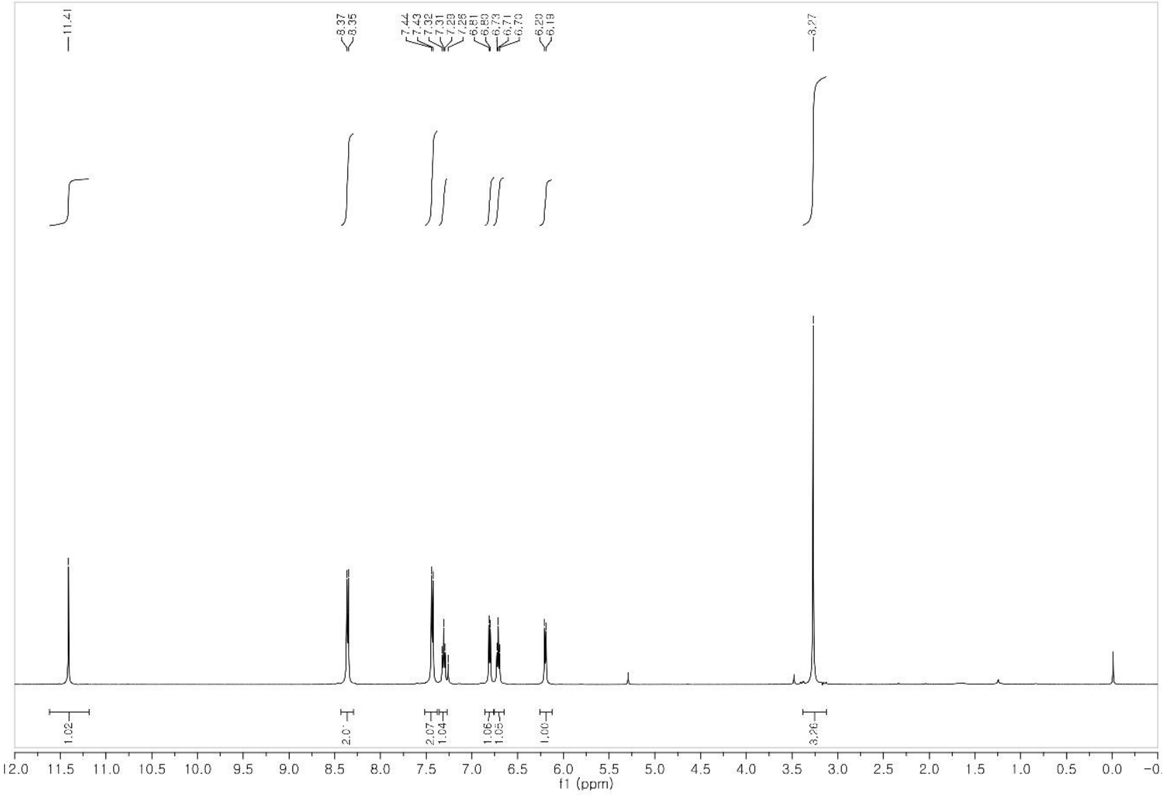

^{13}C NMR

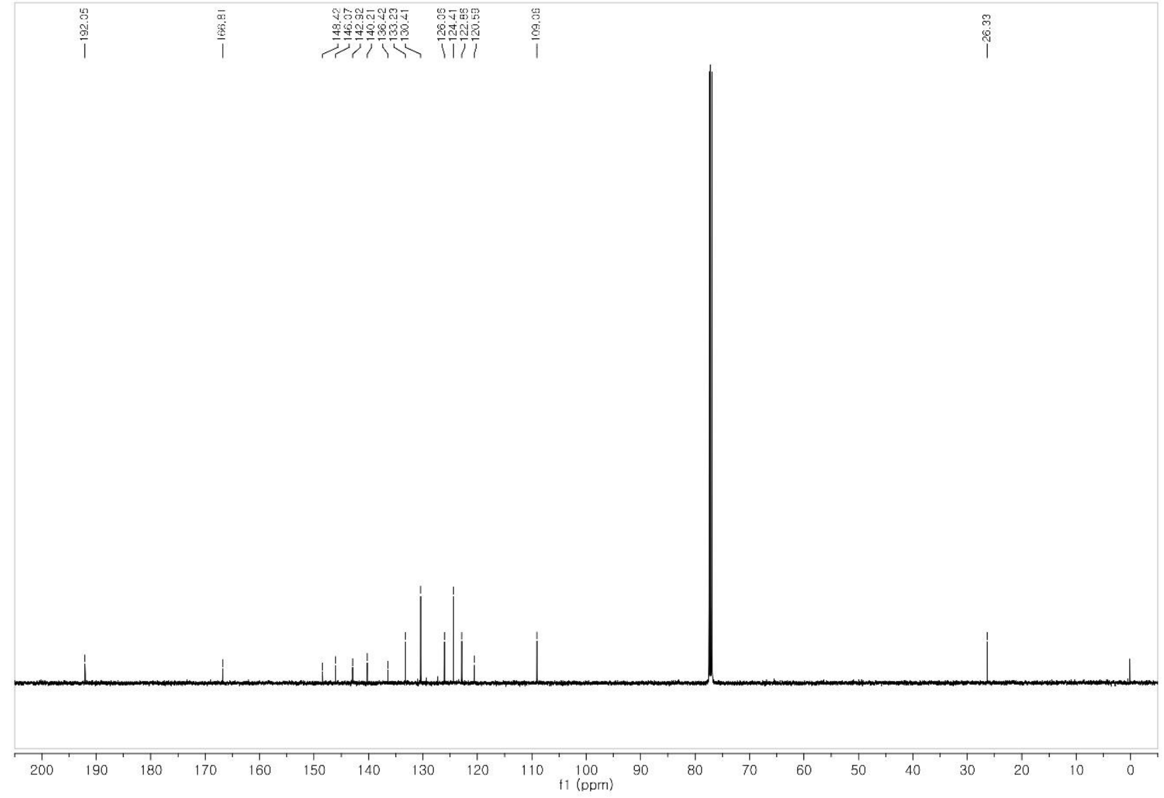

(*Z*)-2-(4-Iodophenyl)-2-(1-methyl-2-oxoindolin-3-ylidene)acetaldehyde (**7e**)

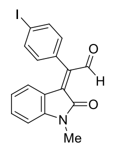

7e

^{1}H NMR

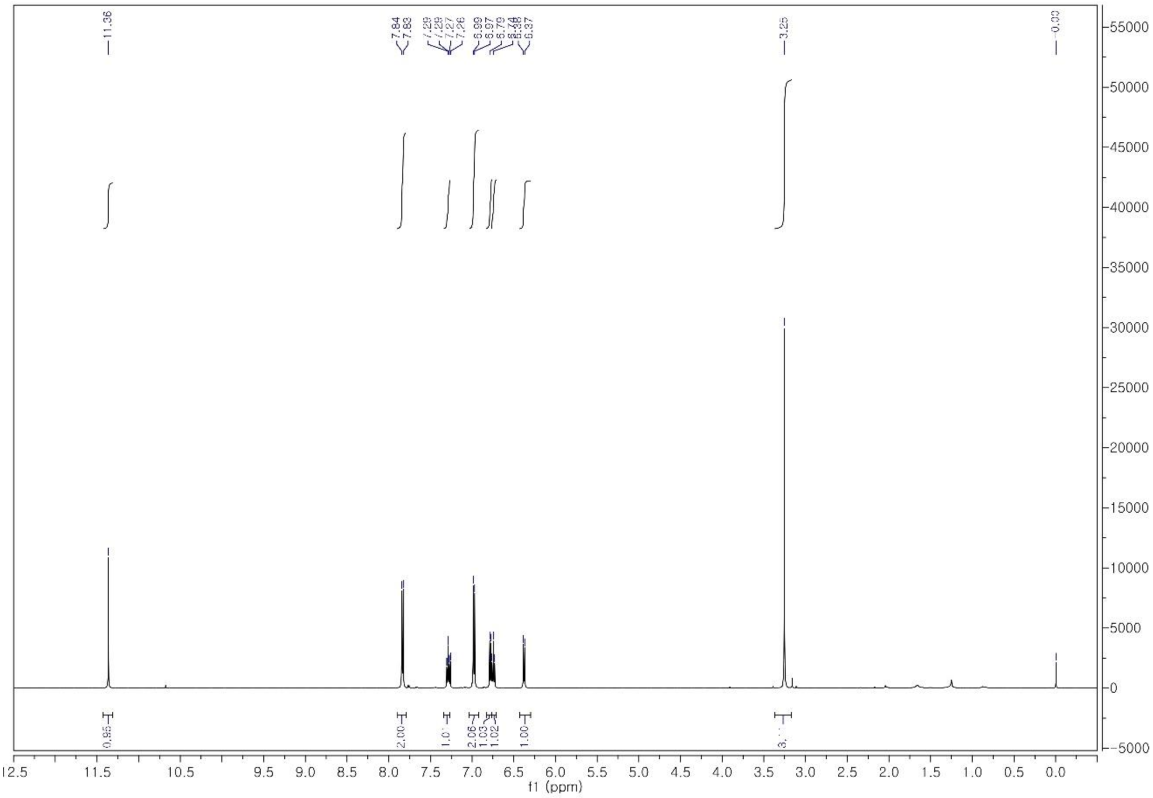

^{13}C NMR

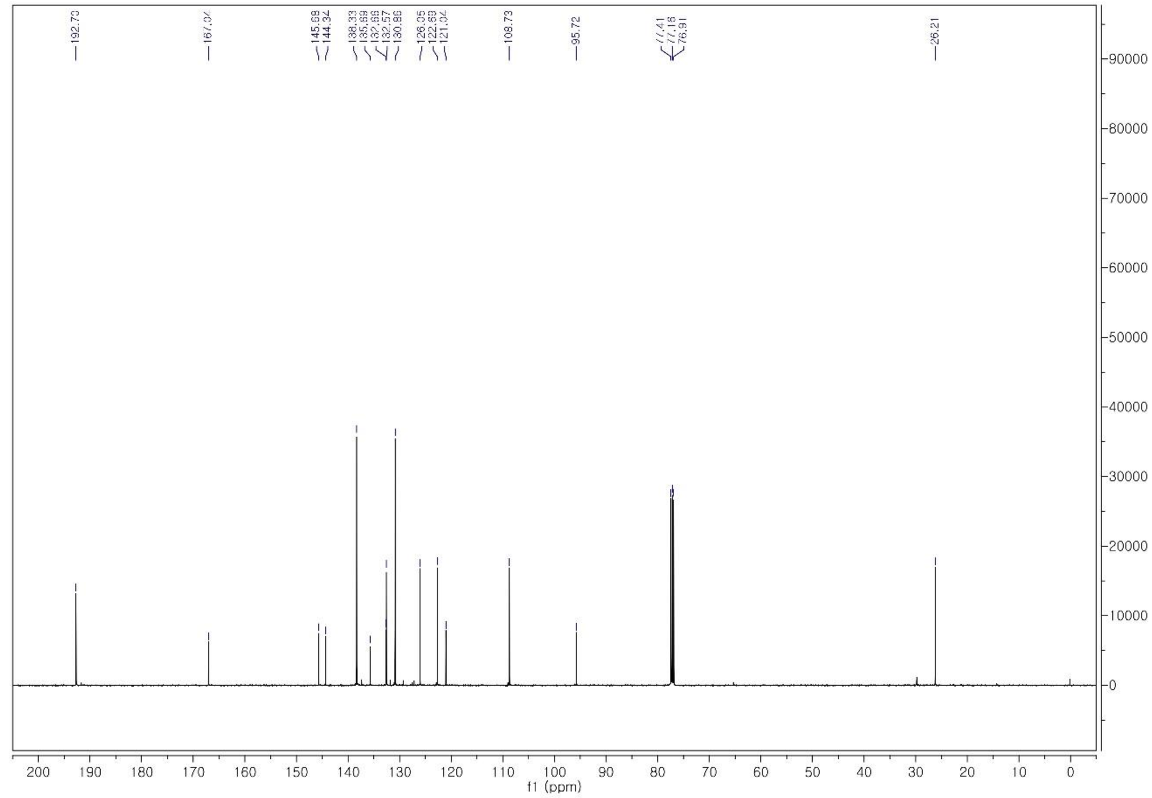

(*E*)-3-(3-Hydroxy-1,3-diphenylpropylidene)-1-methylindolin-2-one (**8aa**)

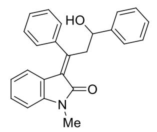

8aa

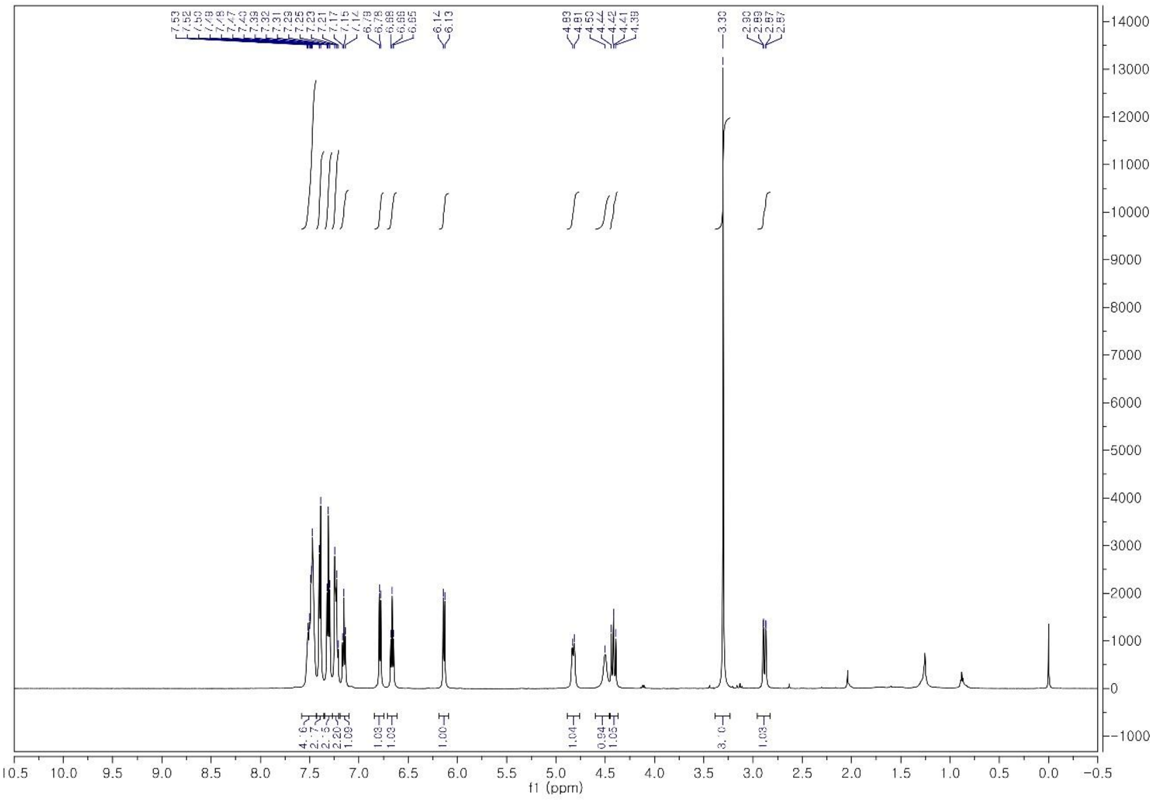

^{1}H NMR

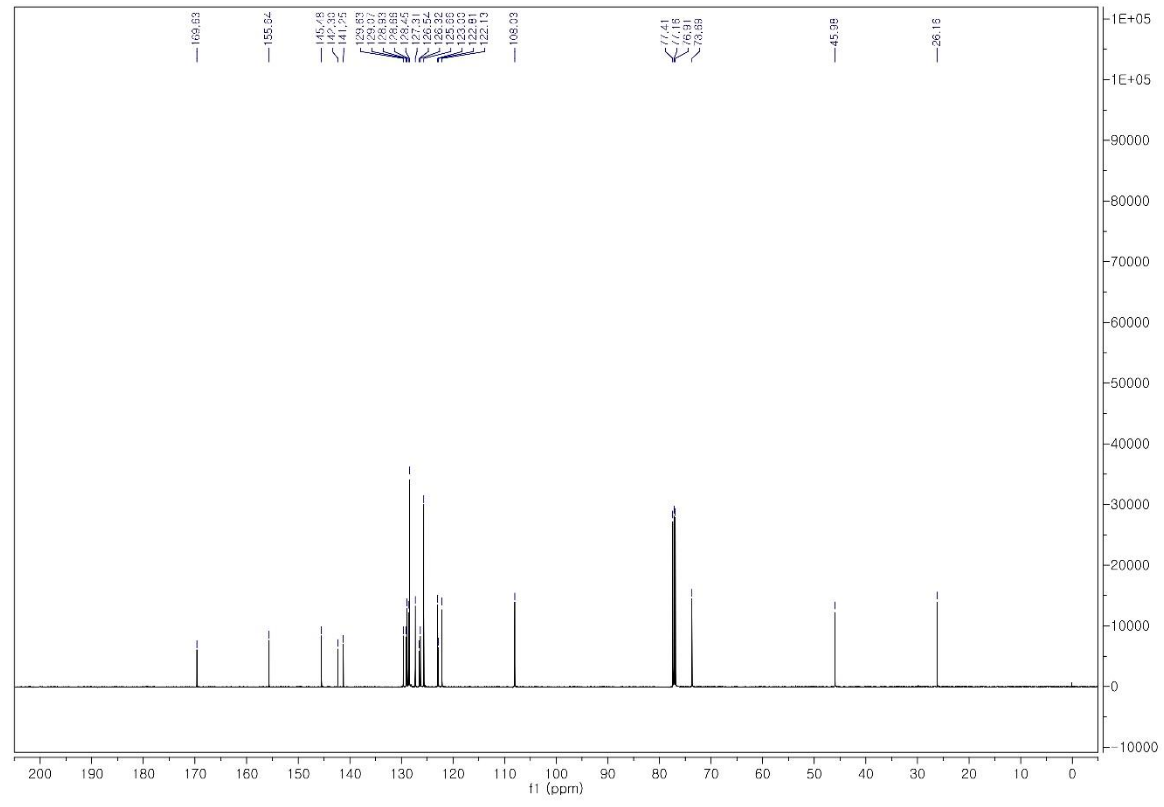

^{13}C NMR

(*E*)-3-(3-Hydroxy-1-(4-methoxyphenyl)-3-phenylpropylidene)-1-methylindolin-2-one (**8da**)

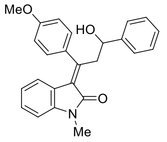

8da

^{1}H NMR

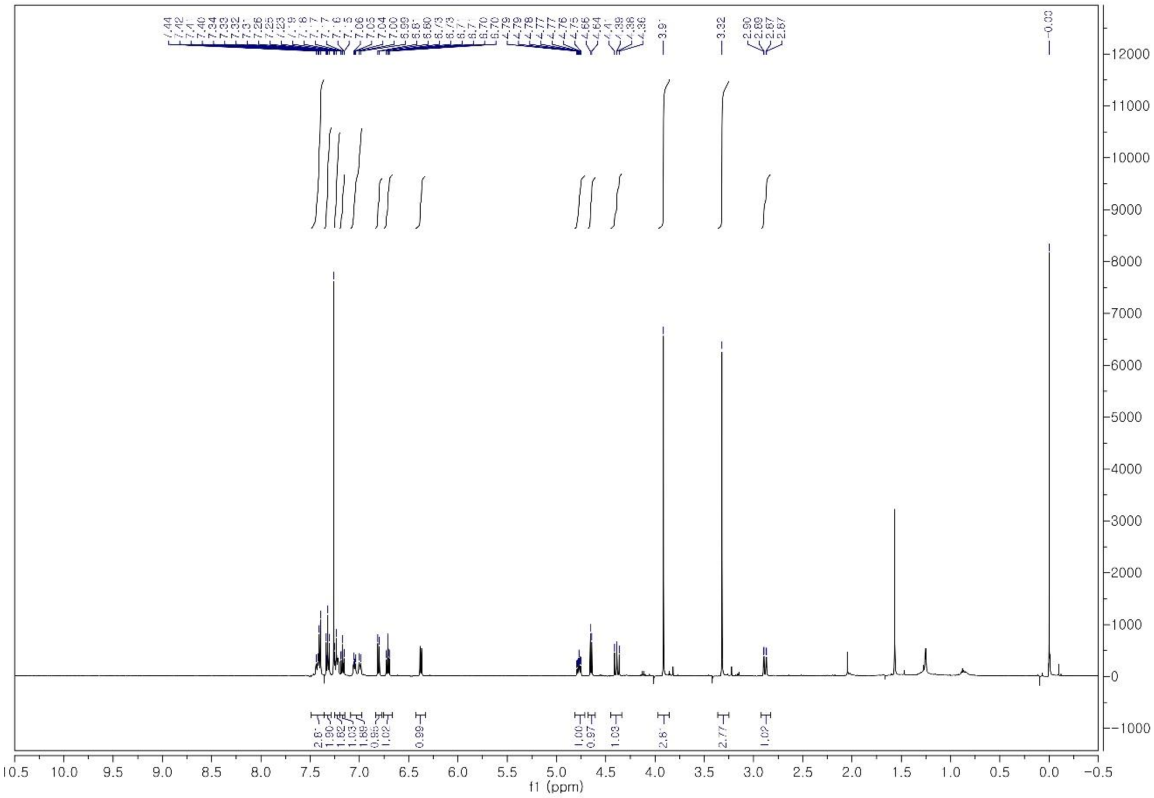

^{13}C NMR

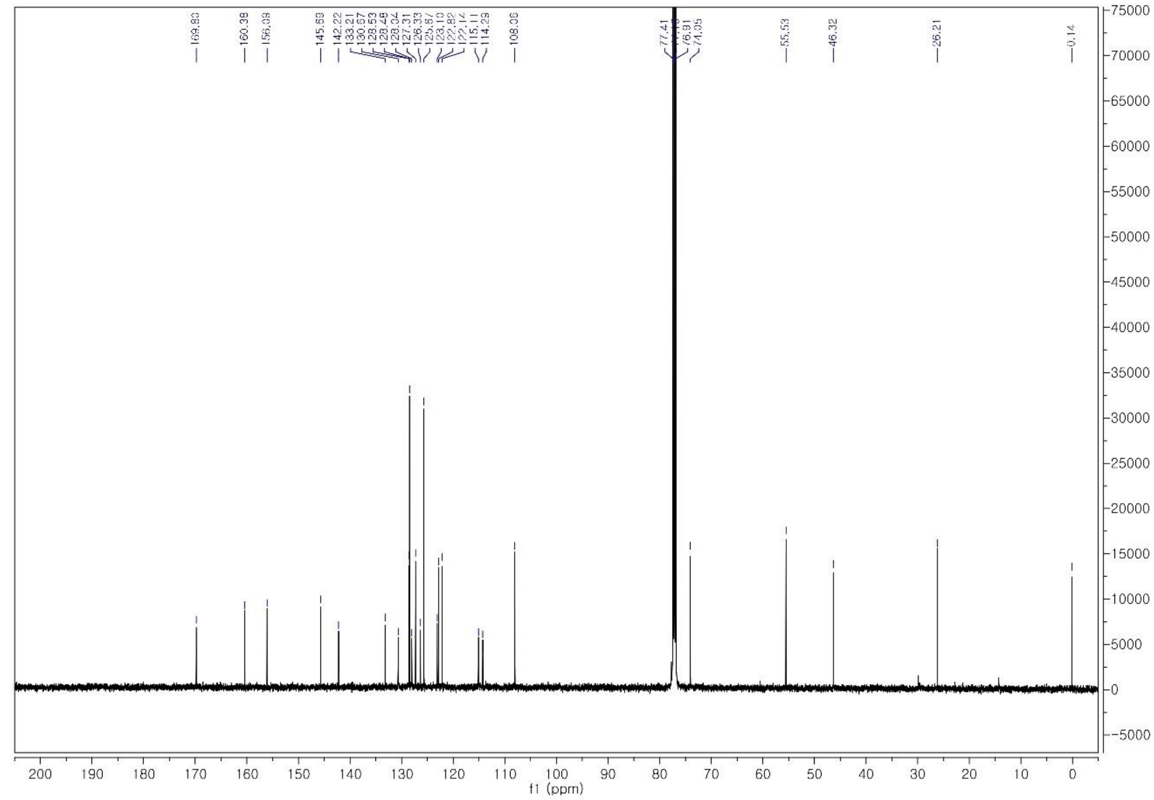

(*E*)-3-(3-(4-Chlorophenyl)-3-hydroxy-1-(4-methoxyphenyl)propylidene)-1-methylindolin-2-one (**8db**)

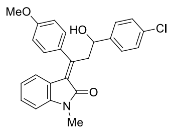

8db

^{1}H NMR

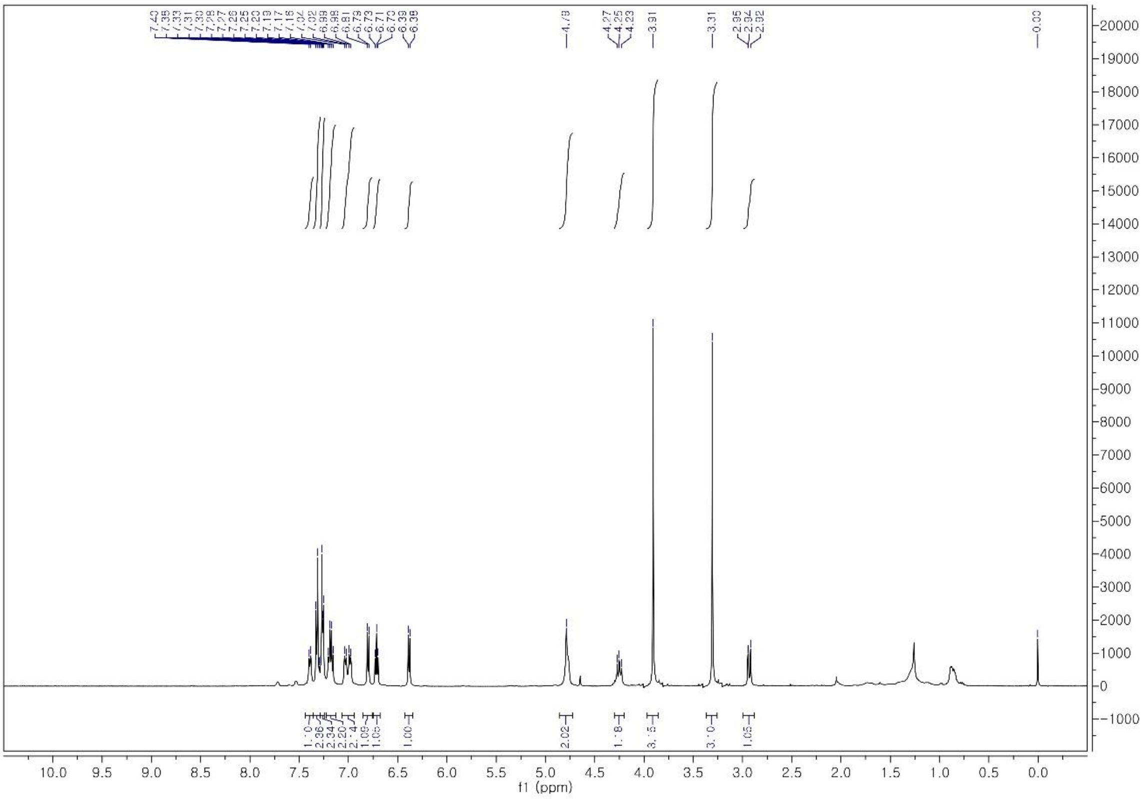

^{13}C NMR

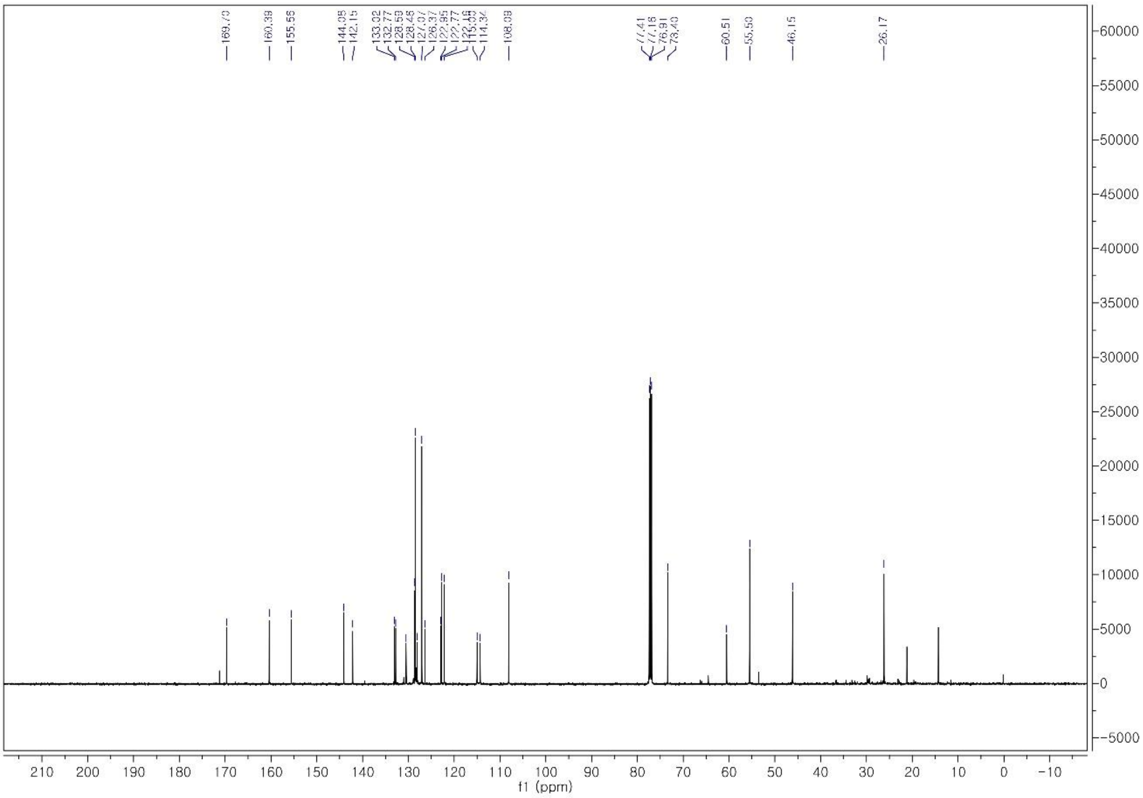

(*E*)-3-(3-Hydroxy-1-(4-methoxyphenyl)-3-(4-nitrophenyl)propylidene)-1-methylindolin-2-one (**8dc**)

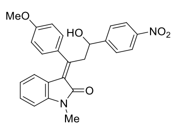

8dc

^{1}H NMR

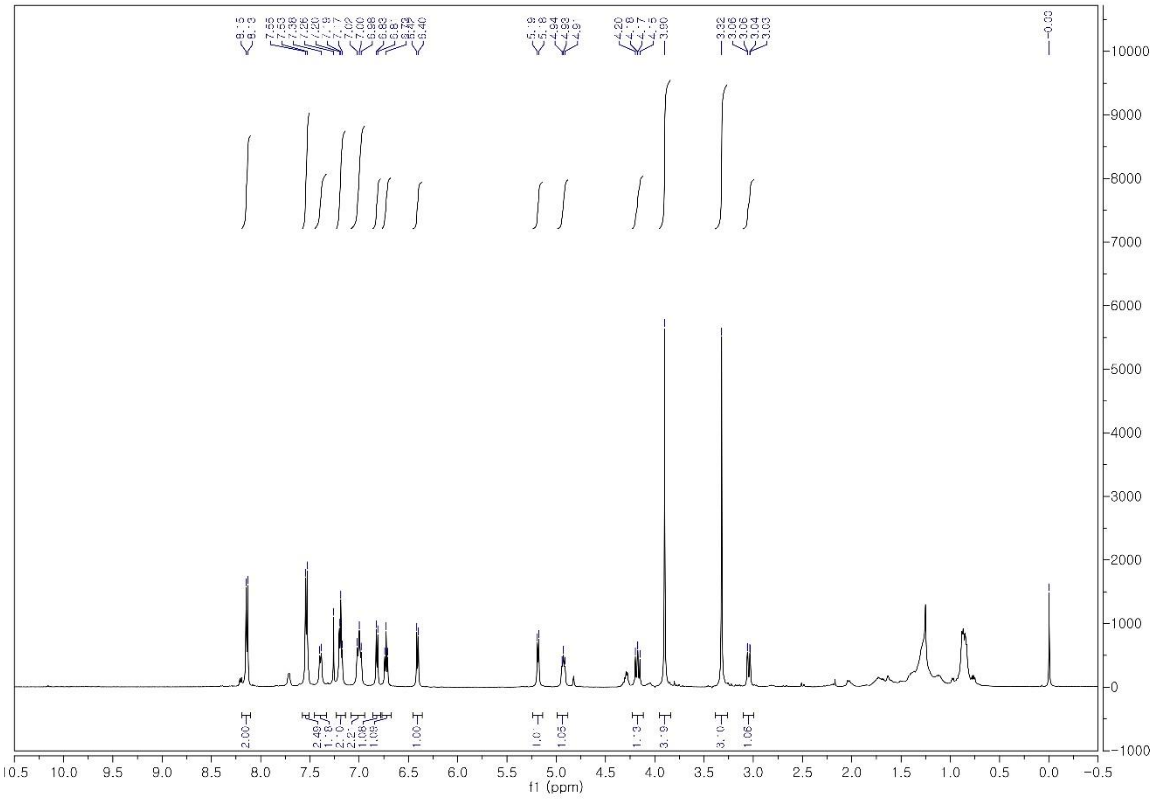

^{13}C NMR

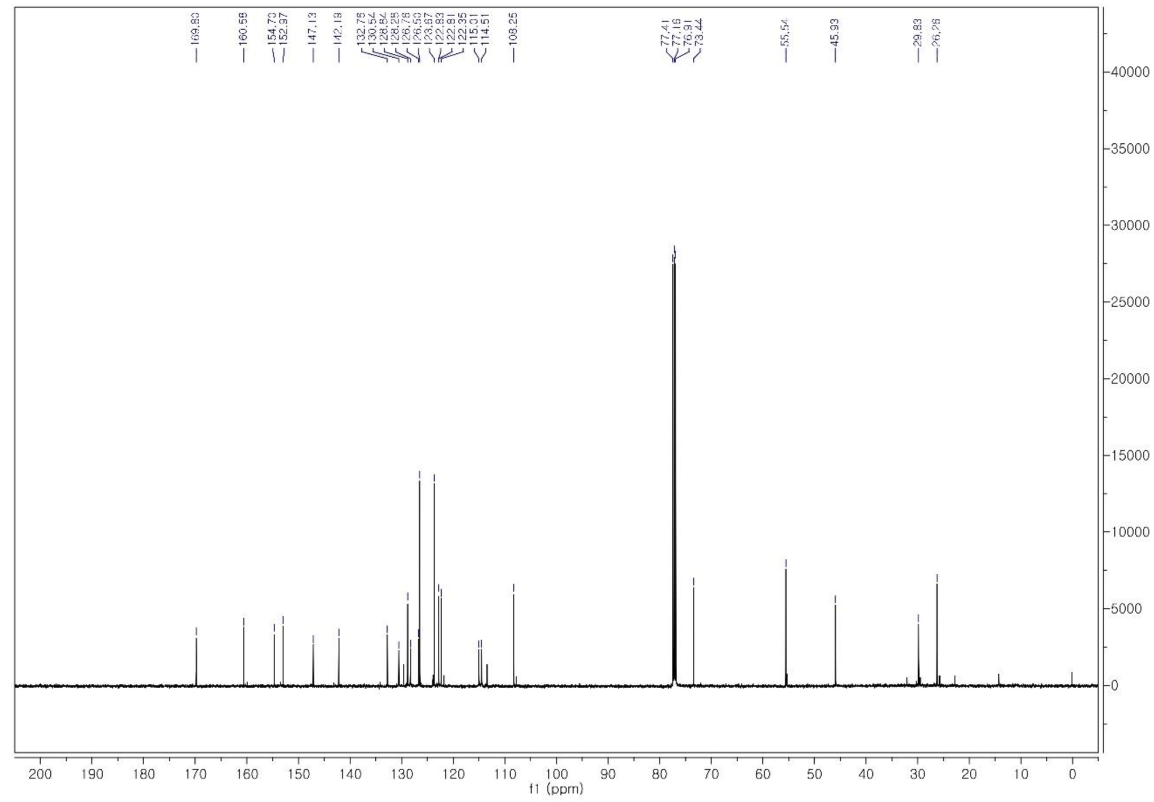

(*E*)-3-(3-Hydroxy-1,3-bis(4-methoxyphenyl)propylidene)-1-methylindolin-2-one (**8dd**)

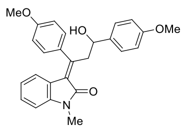

8dd

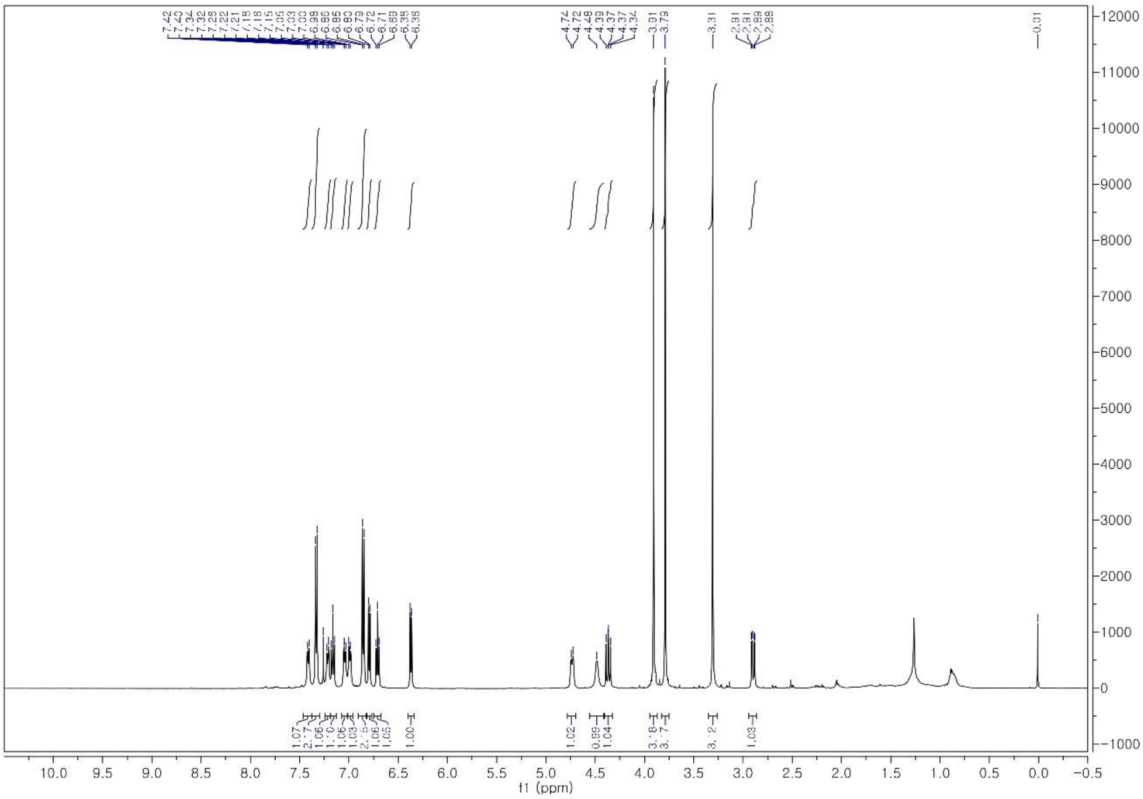

^{1}H NMR

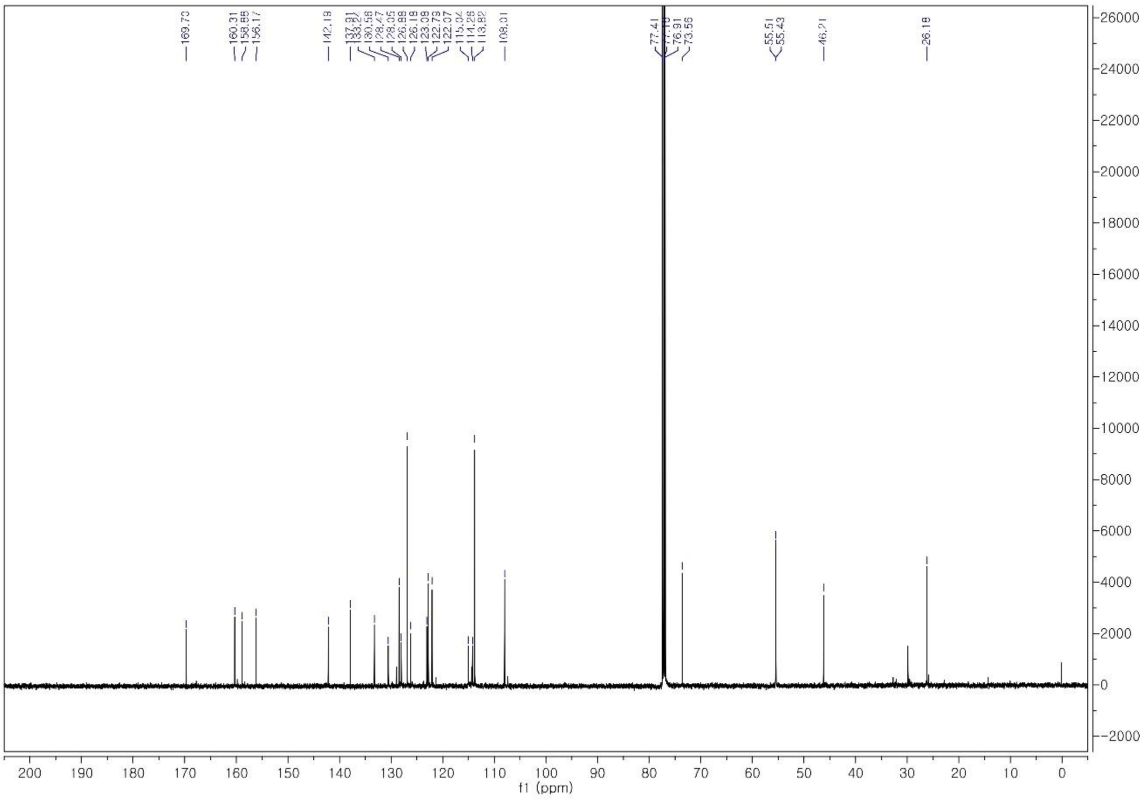

^{13}C NMR

(*E*)-1-Methyl-3-((*E*)-3-(4-nitrophenyl)-1-phenylallylidene)indolin-2-one (**3ac**)

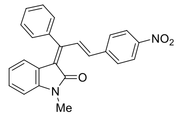

3ac

^{1}H NMR

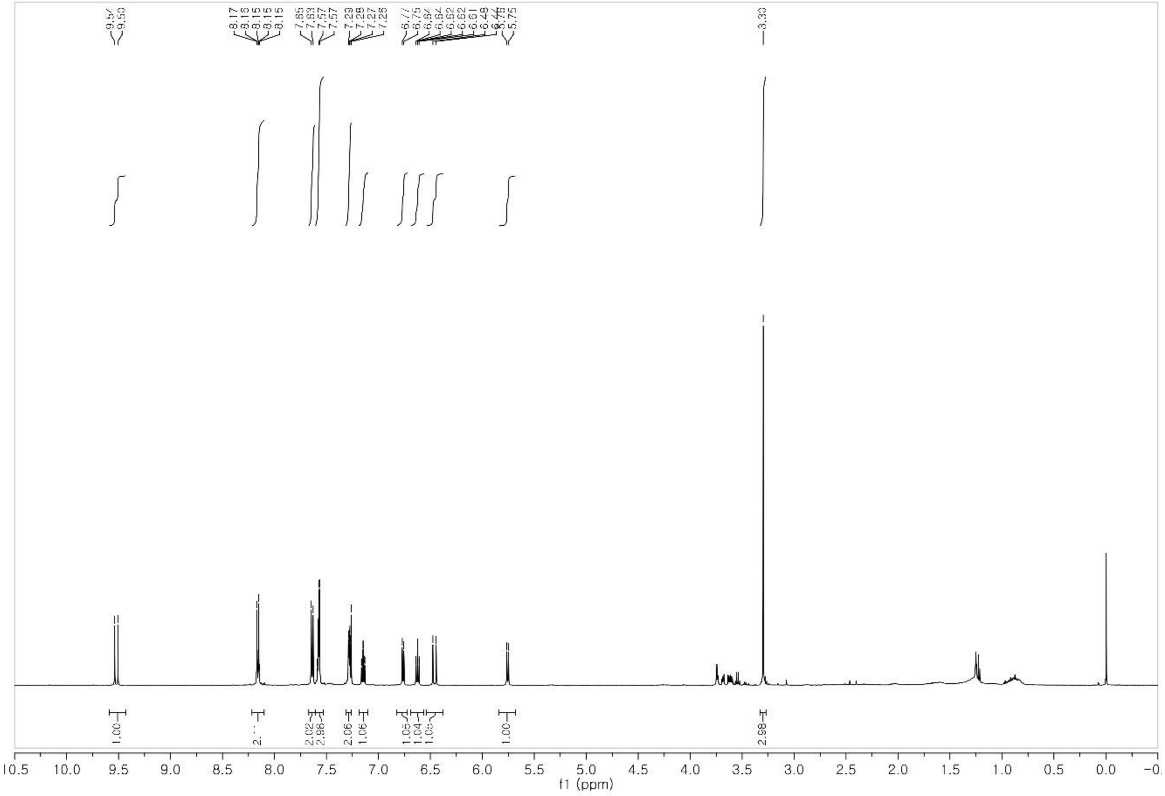

^{13}C NMR

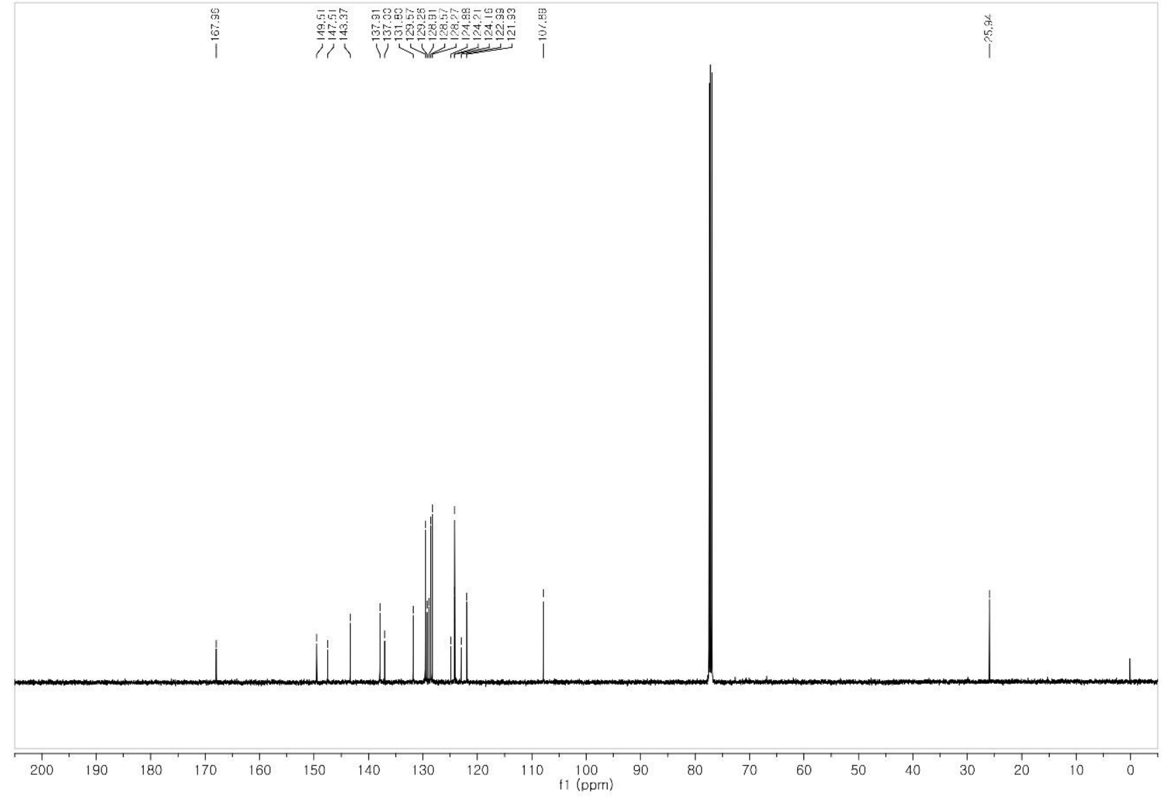

(*E*)-3-((*E*)-1-(4-Chlorophenyl)-3-(4-nitrophenyl)allylidene)-1-methylindolin-2-one (**3bc**)

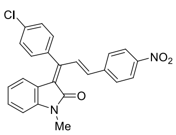

3bc

^{1}H NMR

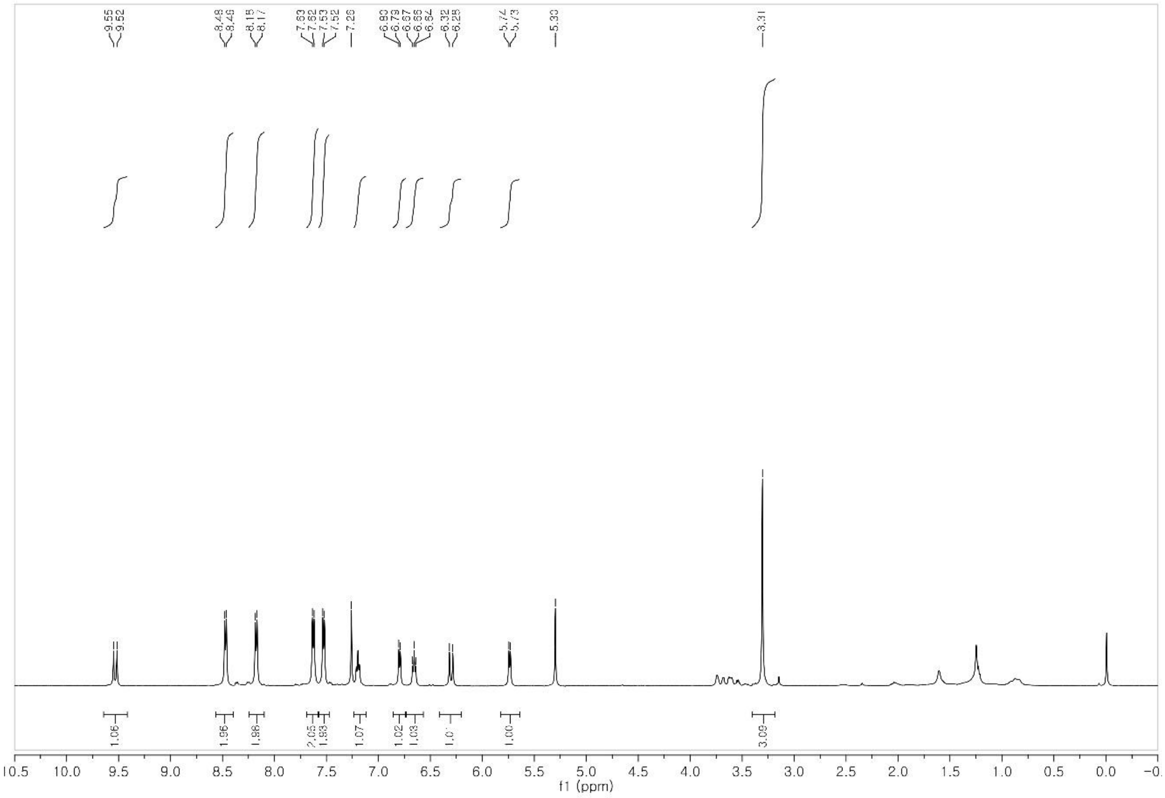

^{13}C NMR

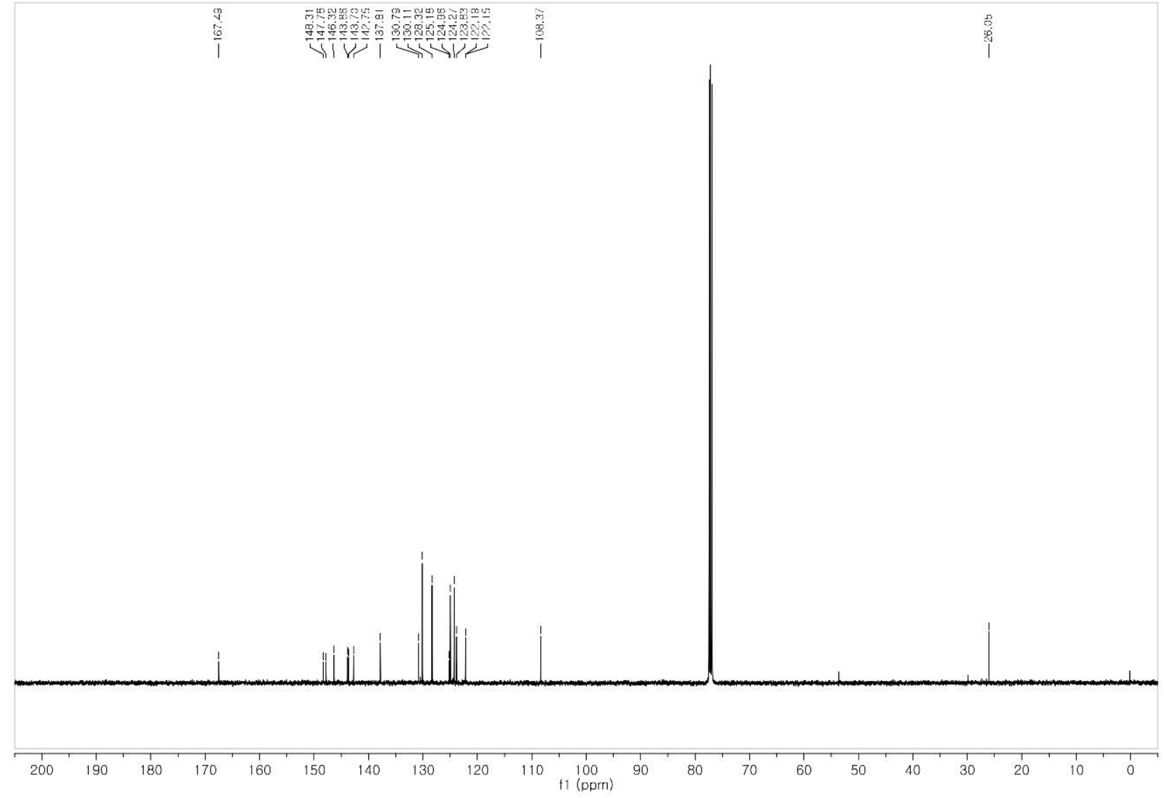

(*E*)-3-((*E*)-1,3-Bis(4-nitrophenyl)allylidene)-1-methylindolin-2-one (**3cc**)

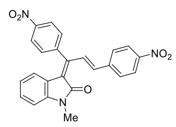

3cc

^{1}H NMR

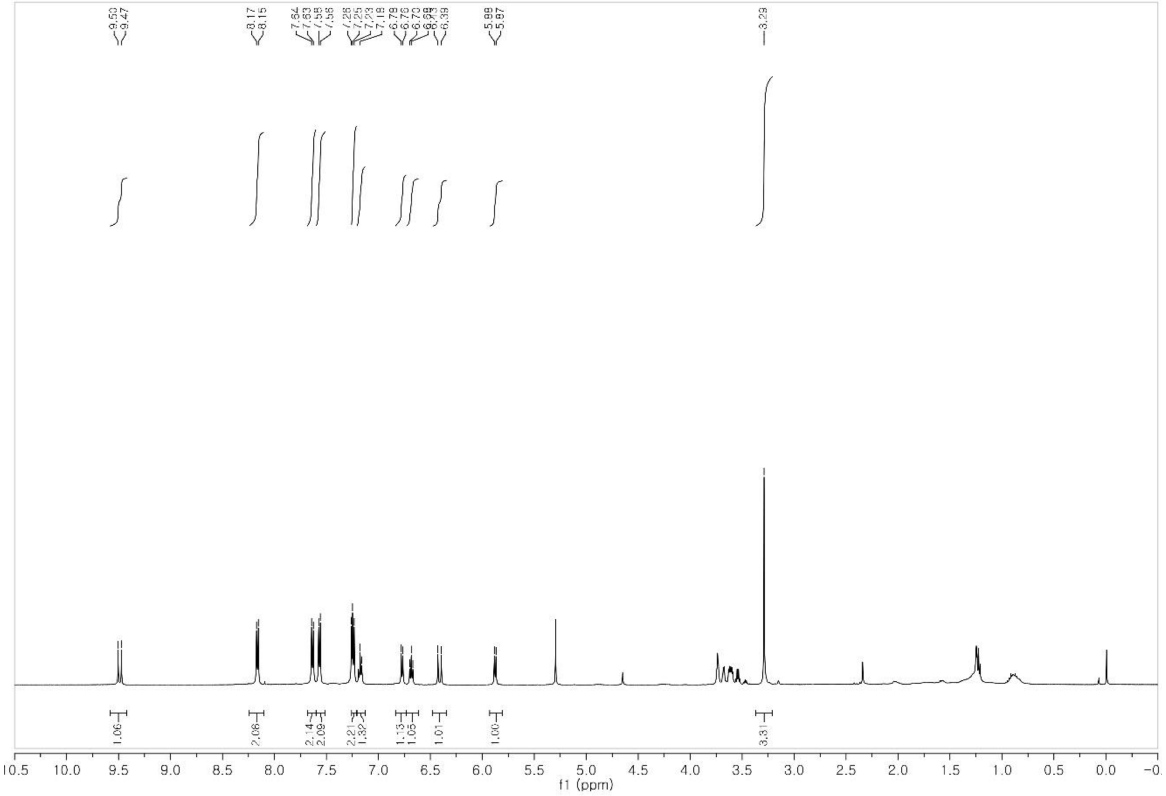

^{13}C NMR

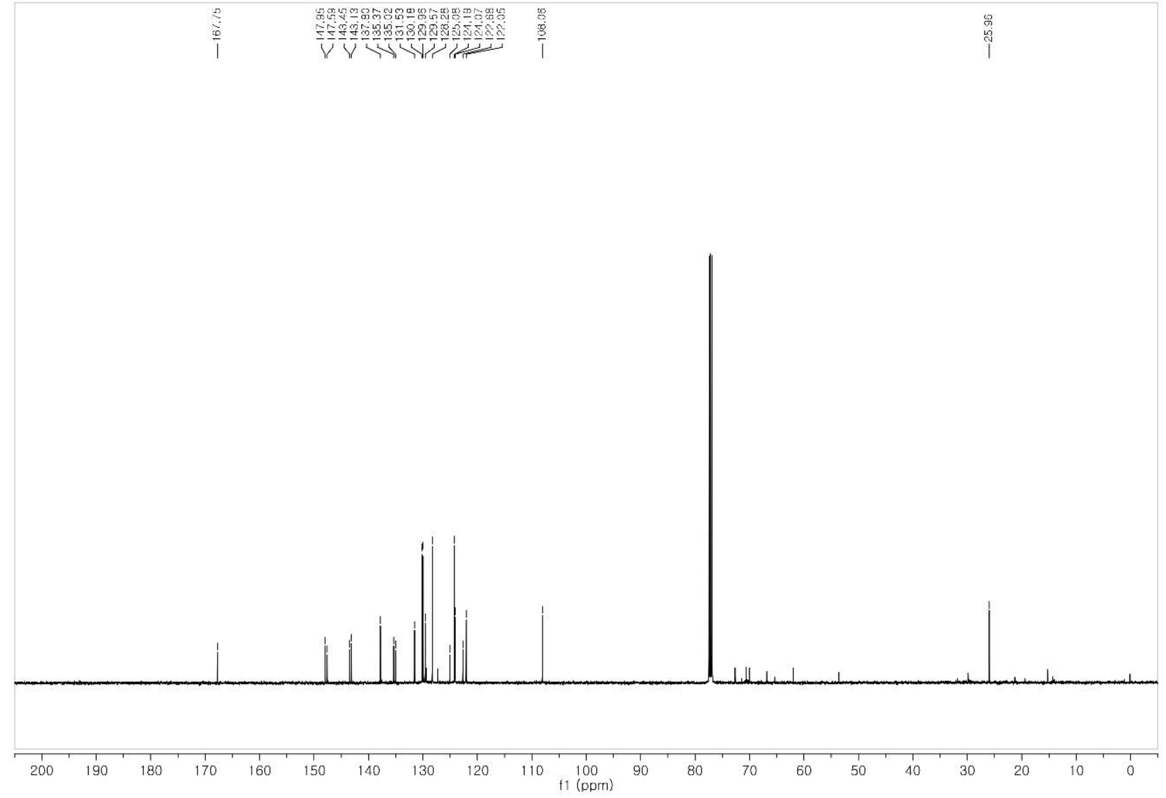

(*E*)-3-((*E*)-1-(4-Methoxyphenyl)-3-(4-nitrophenyl)allylidene)-1-methylindolin-2-one (**3dc**)

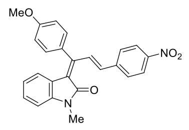

3dc

^{1}H NMR

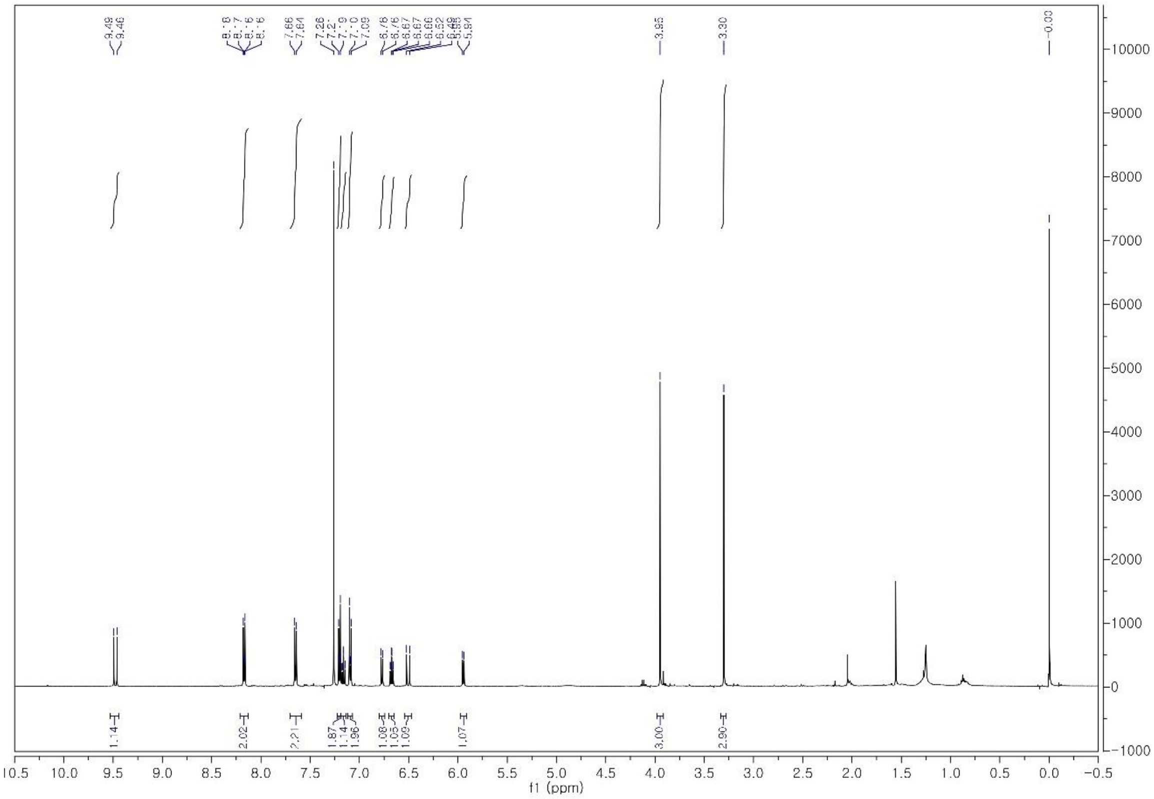

^{13}C NMR

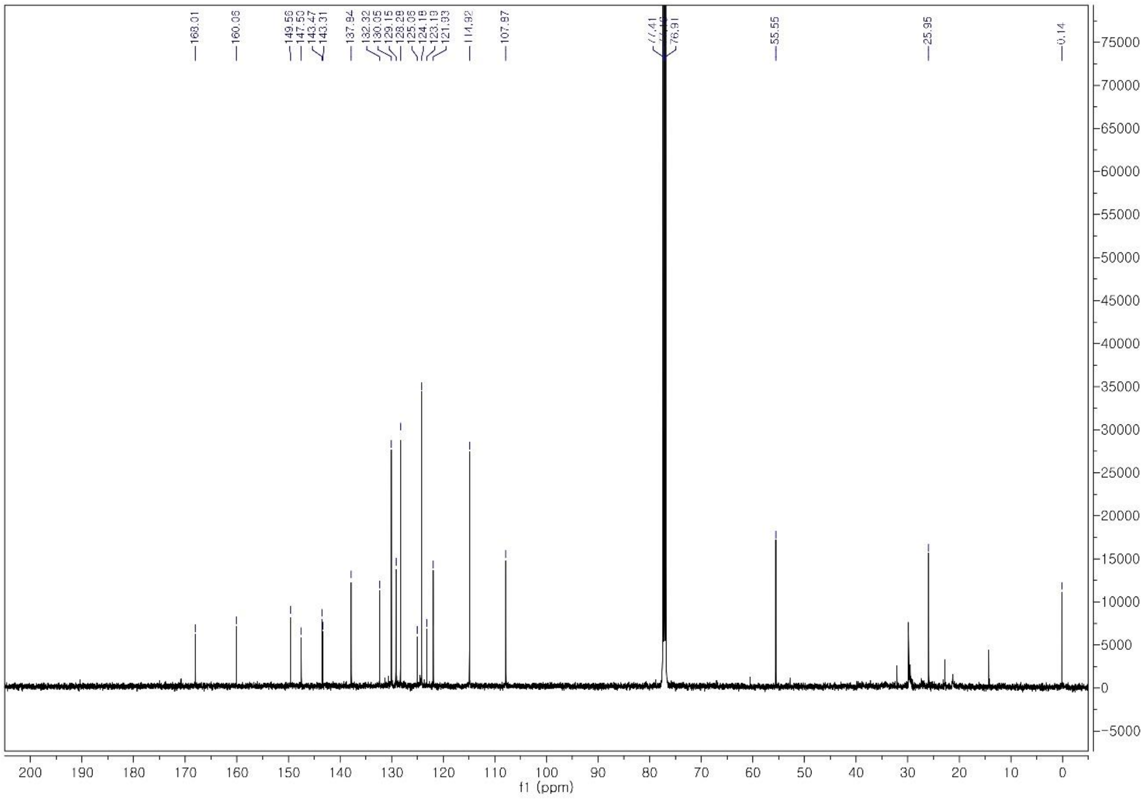

(*E*)-3-(()-1-(4-Iodophenyl)-3-phenylallylidene)-1-methylindolin-2-one (**3ea**)

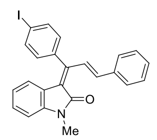

3ea

^{1}H NMR

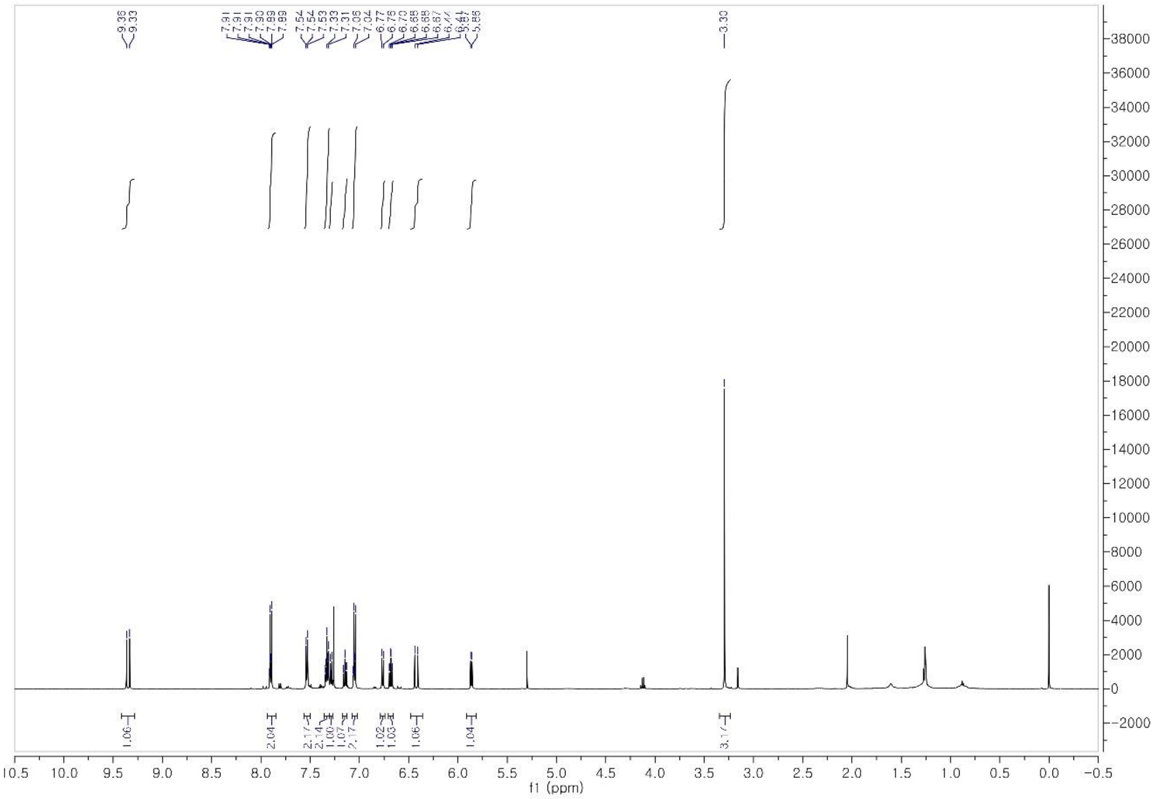

^{13}C NMR

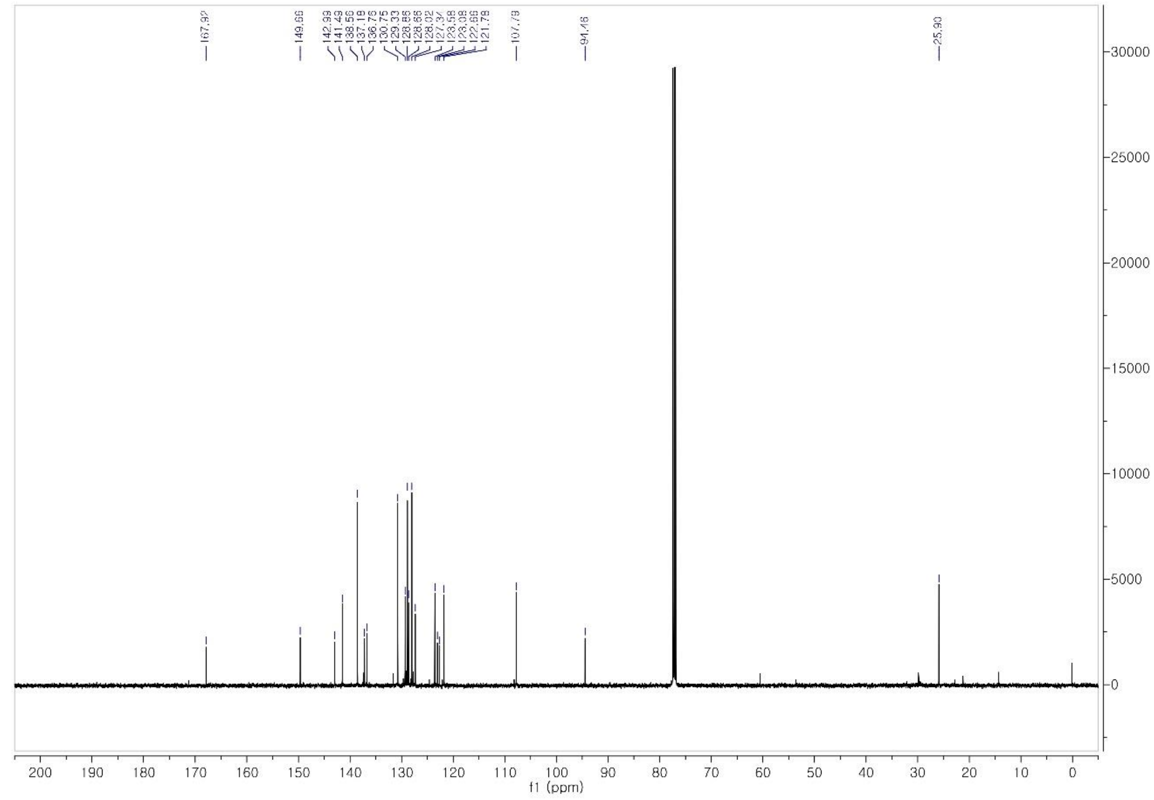

(*E*)-3-((*E*)-1-(Biphenyl-4-yl)-3-phenylallylidene)-1-methylindolin-2-one (**9**)

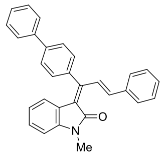

9

^{1}H NMR

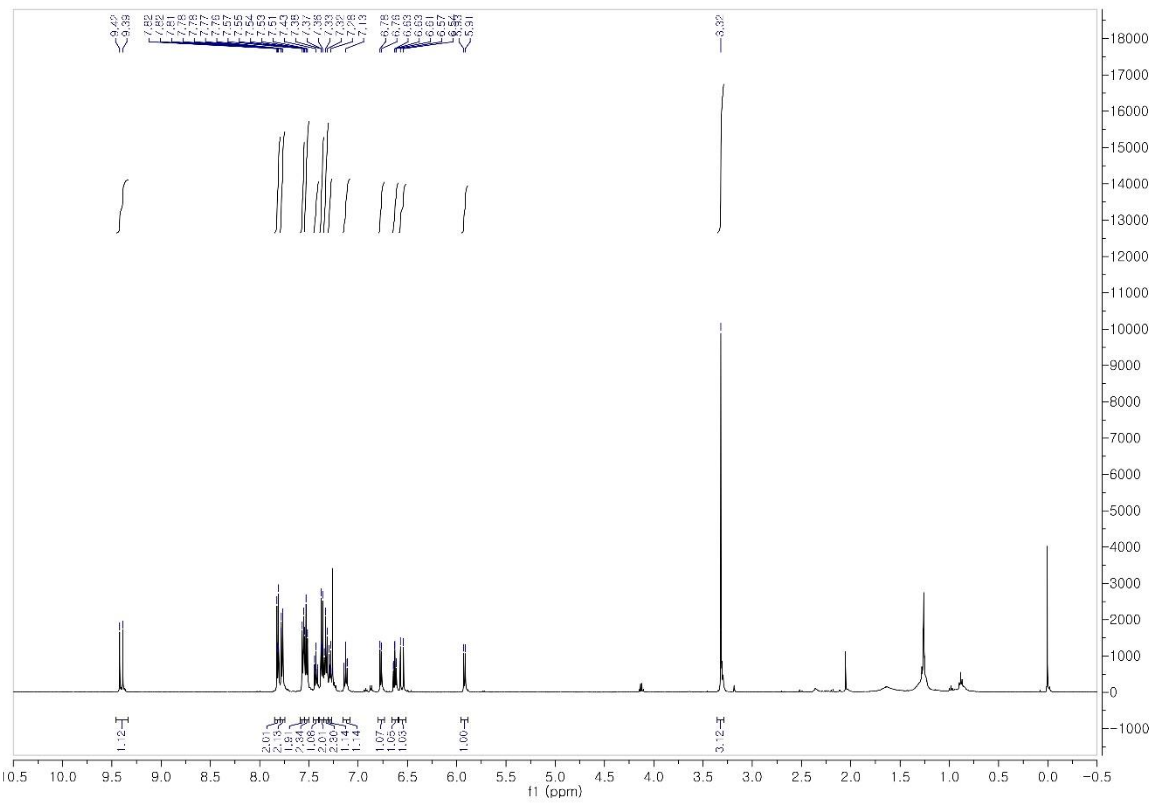

^{13}C NMR

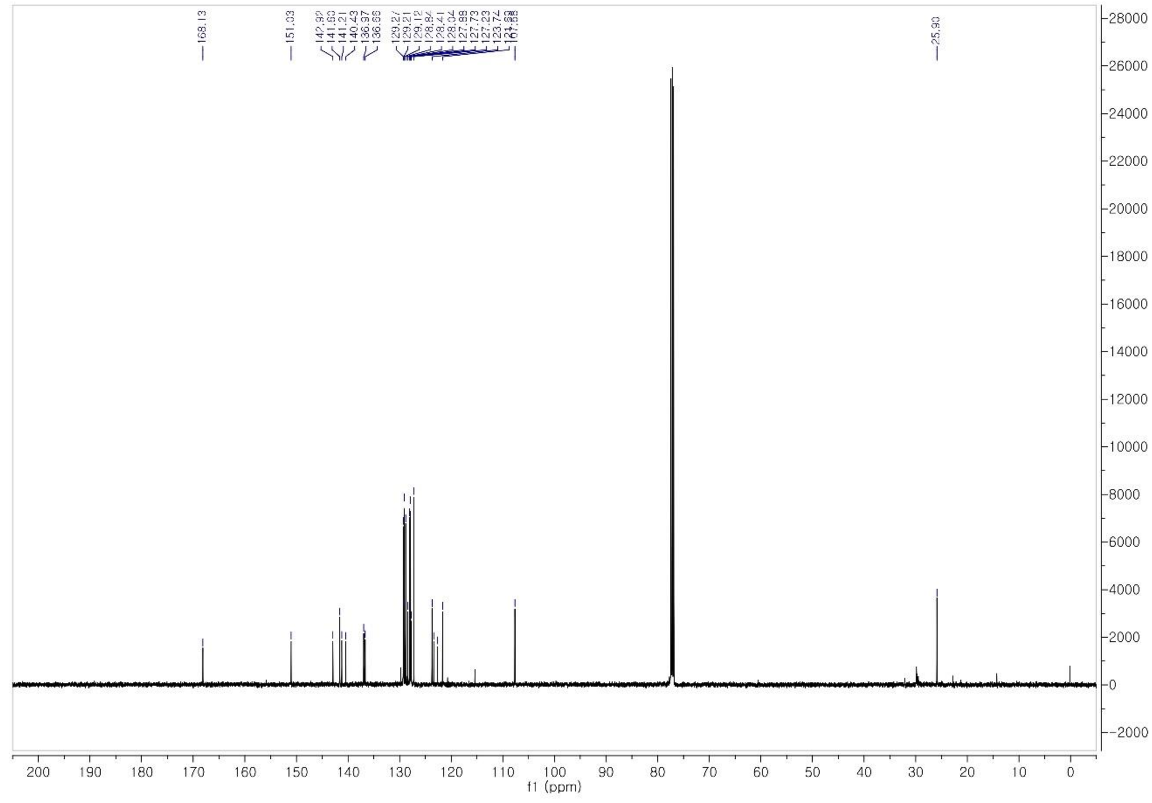

Supplement: Supplementary file 1 [file molecules-27-05304-s001.zip › Supplementary Materials.pdf]
